# Engineering and comparison of *cas12a*‐based genome editing systems in plants

**DOI:** 10.1111/tpj.70410

**Published:** 2025-09-02

**Authors:** Martin Bircheneder, Martin Parniske

**Affiliations:** ^1^ Genetics, Faculty of Biology LMU Munich Grosshaderner Str. 2‐4 D‐82152 Martinsried Germany

**Keywords:** Cas12a, Cas9, genome editing, Csy4 ribonuclease, quantitative knock‐out detection, *Nicotiana benthamiana*, *Lotus japonicus*, *Arabidopsis thaliana*, NLS, intron

## Abstract

While Cas9 and Cas12a are both RNA‐guided endonucleases used for genome editing, only Cas12a is able to process pre‐crRNA via its additional ribonuclease activity. This feature reduces the complexity of Cas12a versus Cas9‐based genome editing systems thus providing an attractive alternative for generating site‐specific mutations in plants. Here we aimed to improve the efficiency of the *cas12a*‐based generation of two double‐strand breaks flanking the open reading frame of a target gene, leading to its full deletion. To this end, we compared the relative impact of different components on *cas12a*‐based gene deletion efficiency in three different eudicotyledons, *Arabidopsis thaliana*, *Lotus japonicus*, and *Nicotiana benthamiana*. We detected the highest *cas12a*‐based editing efficiency with a combination of suitable promoters for crRNA and *cas12a* expression, a tandem terminator to control *cas12a* expression, a re‐coded *cas12a*, adapted to the codon usage of *Arabidopsis* and engineered to carry introns, and encoding a Cas12a flanked by a nuclear localization signal at both ends. Our work revealed the high potential for improving *cas12a*‐based genome editing systems for plant genetic research.

## Significance Statement

Genome editing in plants involves the production of transgenic plants. Efficient genome editing tools have the potential to reduce the number of plants required and thus the cost in space and labor. Here we developed a *cas12a‐*based genome editing system exhibiting high efficiency in all three plant species tested. The system appears suitable for the targeted deletion of genomic regions such as open reading frames or entire genes in plant genomes.

## INTRODUCTION

To determine functional relevance of a gene of interest in plants, deletion of the entire open reading frame (ORF) by two independent double‐strand breaks (DSBs) mediated by *cas*‐based genome editing is a particularly attractive strategy to generate definitive loss‐of‐function mutants (Jinek et al., 2012). This strategy greatly benefits from high editing efficiency, which is strongly influenced by the *cas* system used, which comprises the genes encoding the Cas endonucleases, such as Cas9 and Cas12a to cut specific target sites, and synthetic sequence arrangements facilitating the production of RNA species that match the structural requirement to bind Cas9 or Cas12a and guide them to the specific target site by a guide sequence complementary to target sequence complement (Bernabé‐Orts et al., 2019; Jinek et al., 2012; Zetsche et al., 2015; Zhang et al., 2021). Despite this, comparative analyses remain rarely published (Develtere et al., 2024; Schindele et al., 2023; Schindele & Puchta, 2020; Zhang et al., 2021), suggesting that attempts to improve *cas* systems for their specific target organism or editing function are not standard in the field of plants.

### Comparison of Cas12a with Cas9 endonuclease for plant genome editing

For reverse genetics approaches in plants, a frequently utilized genome editing strategy is the targeted induction of DSBs. While a commonly used endonuclease is Cas9 (Capdeville et al., 2021; Develtere et al., 2024), an alternative is Cas12a (Cpf1) (Bernabé‐Orts et al., 2019; Kim et al., 2017; Lee et al., 2019; Malzahn et al., 2019; Tang et al., 2017; Zetsche et al., 2015); Cas12a appears to be a particularly attractive alternative for plant genome editing because:

1. In contrast to *cas9* with a 5′‐NGG‐3′ protospacer‐adjacent motif (PAM) sequence, *cas12a* requires a T‐rich PAM sequence upstream of the protospacer (Yamano et al., 2016); increasing the number of potential target sites in plant genomes, which are typically AT‐rich.
2. Nuclease activity of Cas12a is mediated by a single RuvC domain, which is successively cleaving both strands, leading to PAM‐distal DSBs with staggered 5′ overhangs (Yamano et al., 2016; Zetsche et al., 2015).
3. Unlike Cas9, Cas12a exhibits ribonuclease activity to convert pre‐crRNA into mature crRNA by itself without requiring tracrRNA and RNase III for processing the mature crRNA. This results in a mature crRNA consisting of the 5′ handle of the direct repeat (DR) and a 20–23 bp spacer sequence binding to the target via complementary base pairing (Zetsche et al., 2015).
4. Key advantages of Cas12a over Cas9 are a demonstrated higher target specificity in plants (Tang et al., 2018) and less reported off‐target effects, which are indistinguishable from spontaneous mutations caused during plant development (Bandyopadhyay et al., 2020; Bernabé‐Orts et al., 2019). Considering these arguments, we decided to explore the potential to improve *cas12a*‐based editing efficiency for the generation of DSBs.

### A quantitative assay for full ORF deletion efficiency

**Figure 1:**
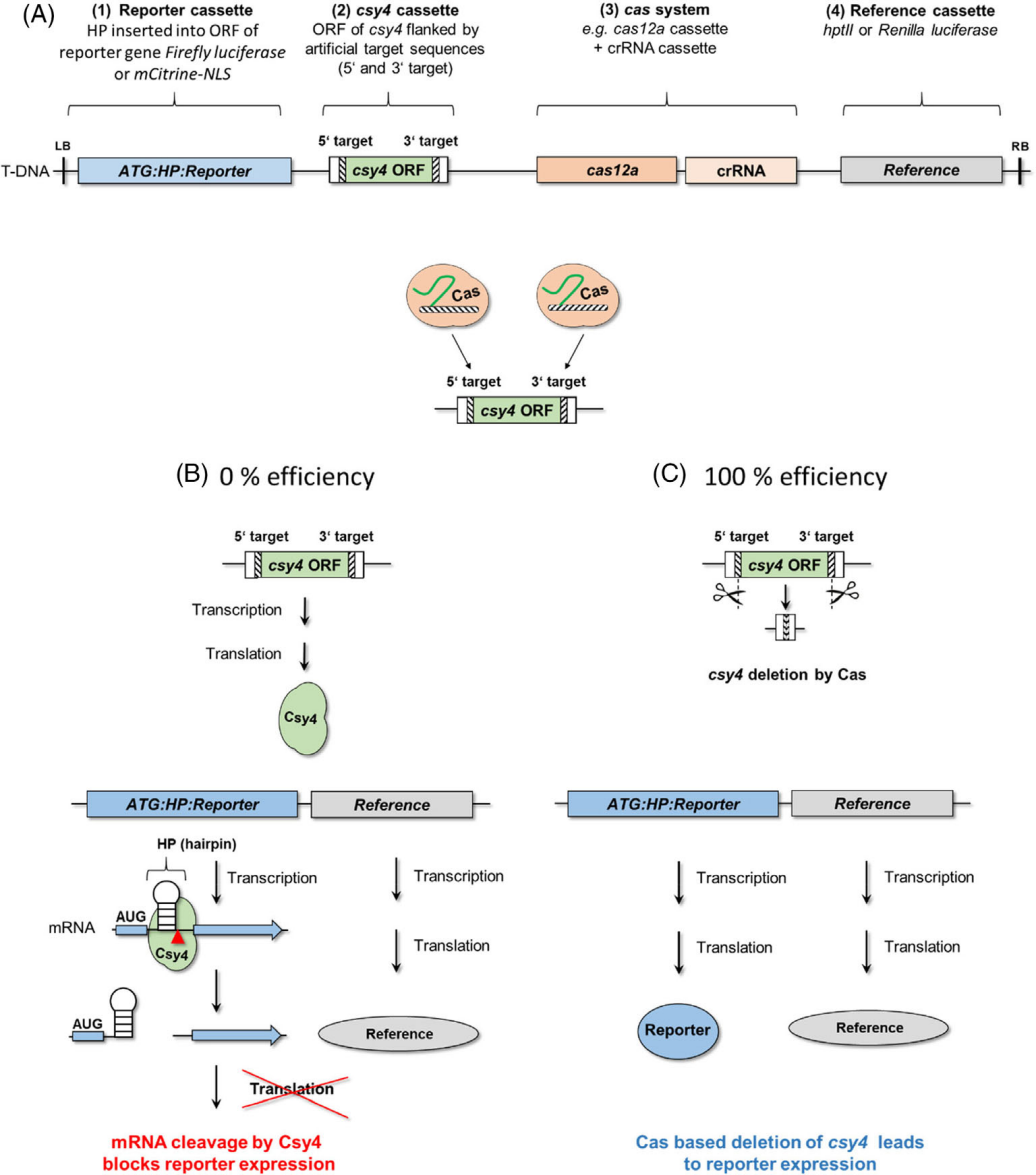
Principle of the quantitative editing efficiency assay. (A) The assay, previously described by Bircheneder et al. (2024), is based on four components which are all located on the same T‐DNA: Reporter cassette *ATG:HP:Reporter* under control of promoter *LjUbi_pro_*, *csy4* cassette targeting the reporter transcript, *cas* system for *csy4* deletion, and a reference gene for normalization or selection. The ORF of *csy4* is under control of promoter *ACT2_pro_* and is flanked by artificial target sequences (5′ and 3′ target). (B) In the absence of Cas‐mediated editing, the *csy4* ORF is maintained and the endoribonuclease Csy4 is expressed. Csy4 recognizes a 28‐nucleotide repeat hairpin (HP) inserted between the start codon (ATG) and the remaining ORF of a reporter gene (*ATG:HP:Reporter*). Csy4 cleaves the reporter mRNA at the inserted HP, thus preventing translation and therefore preventing reporter expression (Borchardt et al., 2015). (C) Cas‐mediated deletion of *csy4* (symbolised by scissors) stabilizes the *ATG:HP:Reporter* mRNA, enabling reporter expression. Thus, the reporter expression level can be taken as a proxy for the detection and quantification of Csy4 loss‐of‐function events.

**Figure 2:**
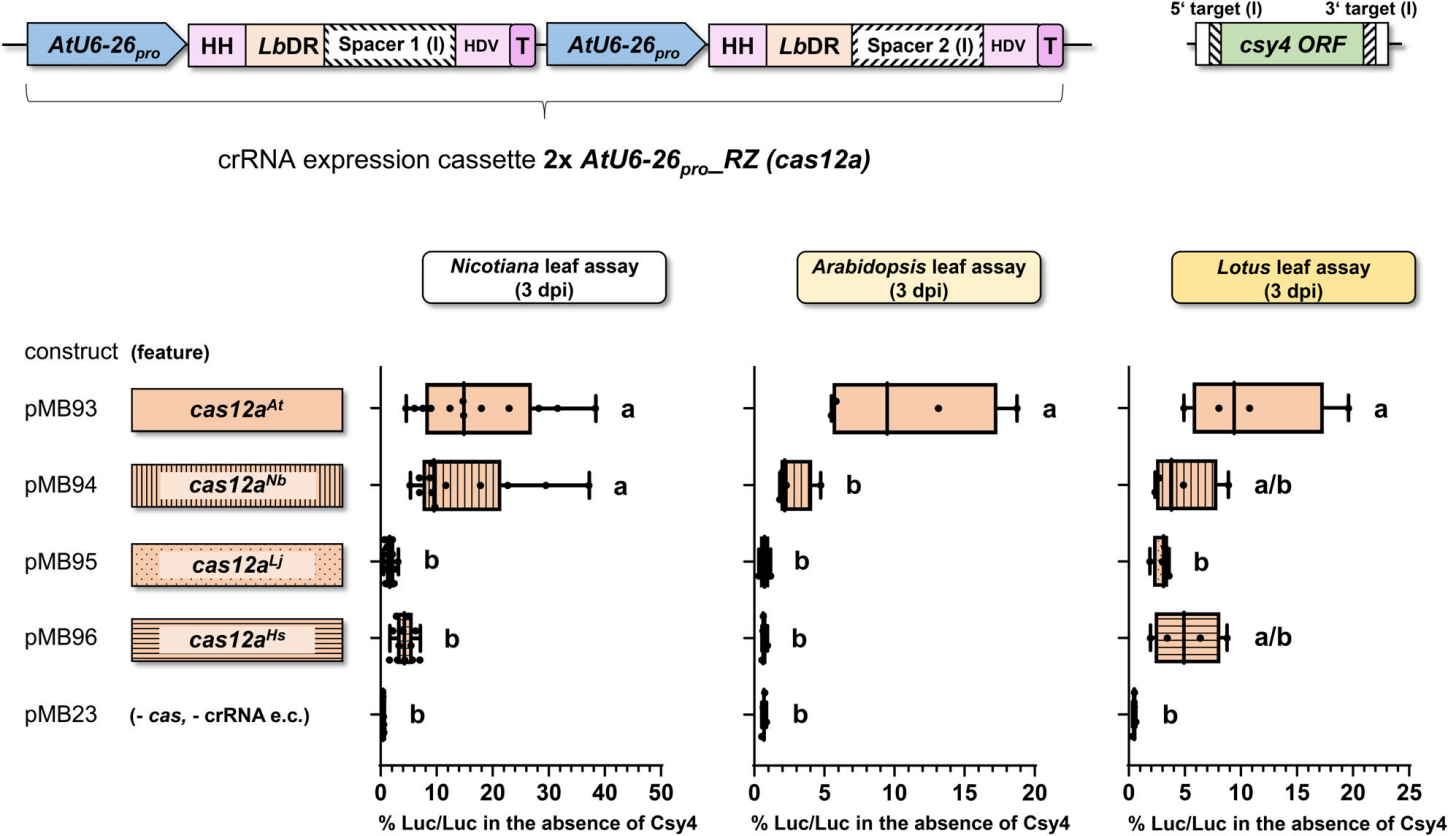
Influence of the codon usage in *cas12a* on editing efficiency. Efficiency of *cas12a* systems with *cas12a* re‐coded with adapted codon usage of *Arabidopsis thaliana* (*cas12a* ^*At*^), *Nicotiana benthamiana* (*cas12a* ^*Nb*^), *Lotus japonicus* (*cas12a* ^*Lj*^), and *Homo sapiens* (*cas12a* ^*Hs*^). Quantification of firefly luciferase activity in *N. benthamiana* leaf disc cells, *A. thaliana* leaf cells, and *L. japonicus* leaf cells, transformed with constructs including the *csy4* cassette with Cas targets version (I). Note, the re‐coded *cas12a* with adapted codon usage of *A. thaliana* led to the highest relative firefly luciferase activity in all three plant species. For details of the *cas* systems used see Figure S4. ORF, open reading frame; *cas12a* ^*At*^, expression cassette of *cas12a* gene with *A. thaliana* (*At*) codon usage; *cas12a* ^*Nb*^, *cas12a* with *N. benthamiana* (*Nb*) codon usage; *cas12a* ^*Lj*^, *cas12a* with *L. japonicus* (*Lj*) codon usage; *cas12a* ^*Hs*^, *cas12a* with human (*Hs*) codon usage; e.c., expression cassette; % Luc/Luc, firefly luciferase activity normalized to *Renilla* luciferase activity; one‐way ANOVA followed by Tukey test was performed for the whole data set; values with no significant difference to each other were grouped by letters (a, b). Values labeled with different letters are statistically significantly different. Values labeled with two letters are statistically not different to values with corresponding single letters. One‐way ANOVA: *P* < 0.0001.

**Figure 3:**
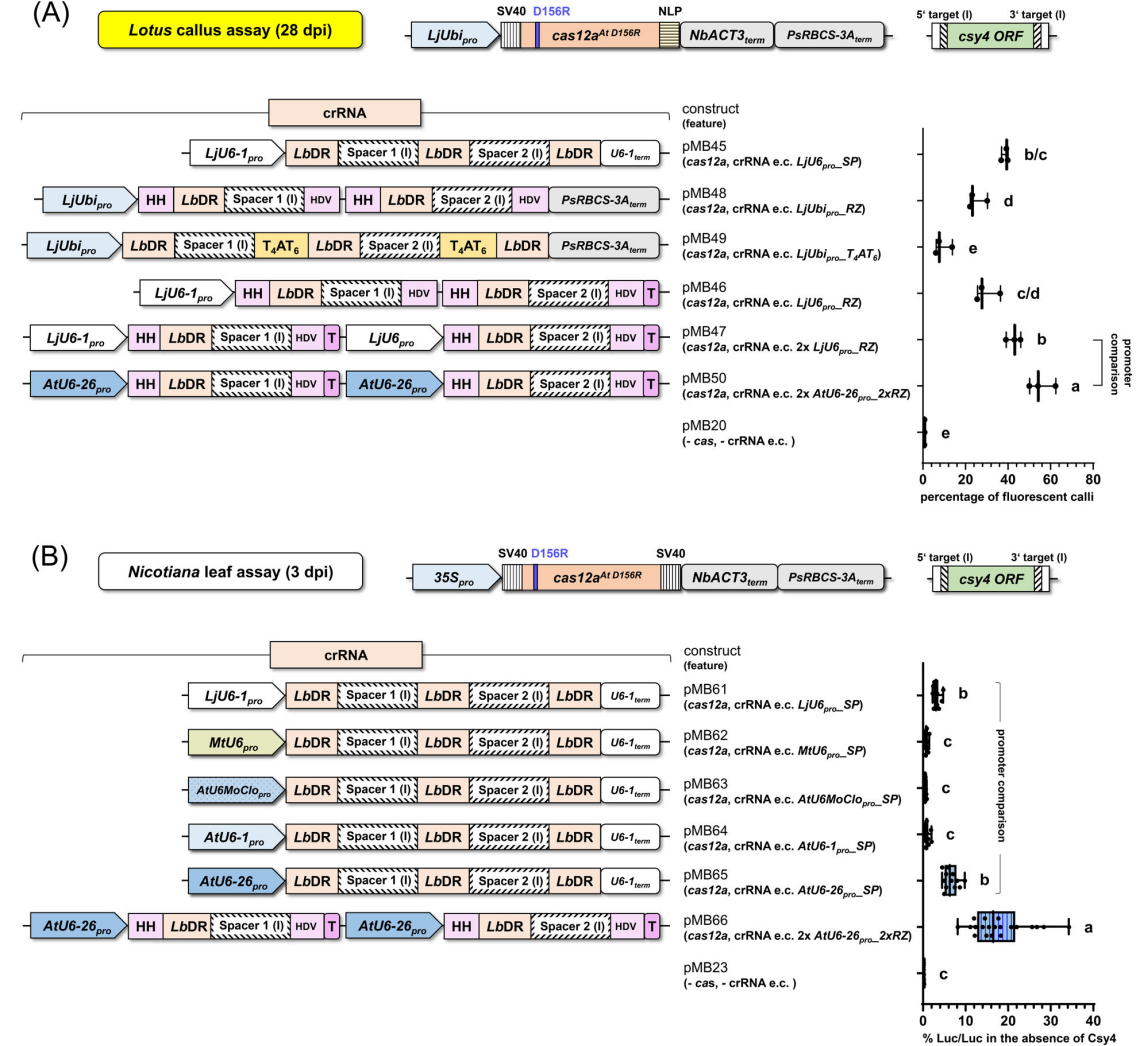
Influence of different crRNA expression cassettes on editing efficiency. Efficiency of different crRNA expression cassettes (e.c.) in two different assay systems. (A) and (B) top line: Assay system and *cas* system components used. Expression of *cas12a* was driven by *LjUbi*_*pro*_ and the Cas12a protein was fused to a SV40 nuclear localization signal (NLS) at the N‐terminus and for (A) an NLP NLS and (B) an SV40 NLS at the C‐terminus. All constructs used carried the indicated *csy4* target sequence versions (I). For the comparison of these cassettes with the target sequence versions (II) see Figure S1. (A) Ratio of living fluorescent to non‐fluorescent calli after transformation of *L. japonicus* hypocotyl cells. Note that the crRNA expression cassette in pMB50 led to the highest editing efficiency. (B) Quantification of firefly luciferase activity in *N. benthamiana* leaf cells transformed with the indicated constructs. The SP crRNA expression system was used throughout this comparison. Note that in the single promoter set‐up (“promoter analysis”) pMB65 featuring a single *AtU6‐26*_*pro*_ led to the strongest relative firefly activity. Importantly, pMB66 in which two promoters in “2x *AtU6‐26*_*pro*_ *_2xRZ (cas12a)*” were utilized, led to a significantly higher editing efficiency than any of the single promoter crRNA cassettes. For a detailed description of the constructs see Figure S6. *35S*_*pro*_, Cauliflower mosaic virus *35S* *RNA* gene promoter; *LjUbi*_*pro*_, *Lotus japonicus* *polyubiquitin* promoter; SV40, nuclear localization signal (NLS) of the Simian Virus 40; NLP, NLS from nucleoplasmin of *Xenopus laevis*; *cas12a* ^*At D156R*^, *A. thaliana* (*At*) codon‐adapted *cas12a* gene encoding the D156R replacement; *crRNA* expression cassettes (e.c.) with Spacer sequences (I) (Appendix S1–S8; Table S4); *Lb*DR, *Lachnospiraceae* bacterium (*Lb*) direct repeat; HH, hammerhead ribozyme; HDV, hepatitis delta virus ribozyme; *AtU6‐16*_*pro*_, *Arabidopsis thaliana (At)* RNA polymerase III promoter *U6‐1*; *AtU6‐26*_*pro*_, RNA polymerase III promoter *U6‐26*; *AtU6MoClo*_*pro*_, RNA polymerase III promoter *U6MoClo*; *MtU6*_*pro*_, *Medicago truncatula (Mt)* RNA polymerase III promoter *U6*; *LjU6‐1*_*pro*_, *Lotus japonicus (Lj)* RNA polymerase III promoter *U6‐1*; T, poly‐T; T_4_AT_6_, nucleotide sequence TTTTATTTTTT; *Ps*, *Pisum sativum*; *Nb*, *Nicotiana benthamiana*; % Luc/Luc, firefly luciferase activity normalized to *Renilla* luciferase activity; one‐way ANOVA followed by Tukey test was performed for the whole data set, and values with no significant difference to each other were grouped by letters (a–e). Values labeled with different letters are statistically significantly different. Values labeled with two letters are statistically not different to values with corresponding single letters. One‐way ANOVA: *P* < 0.0001.

**Figure 4:**
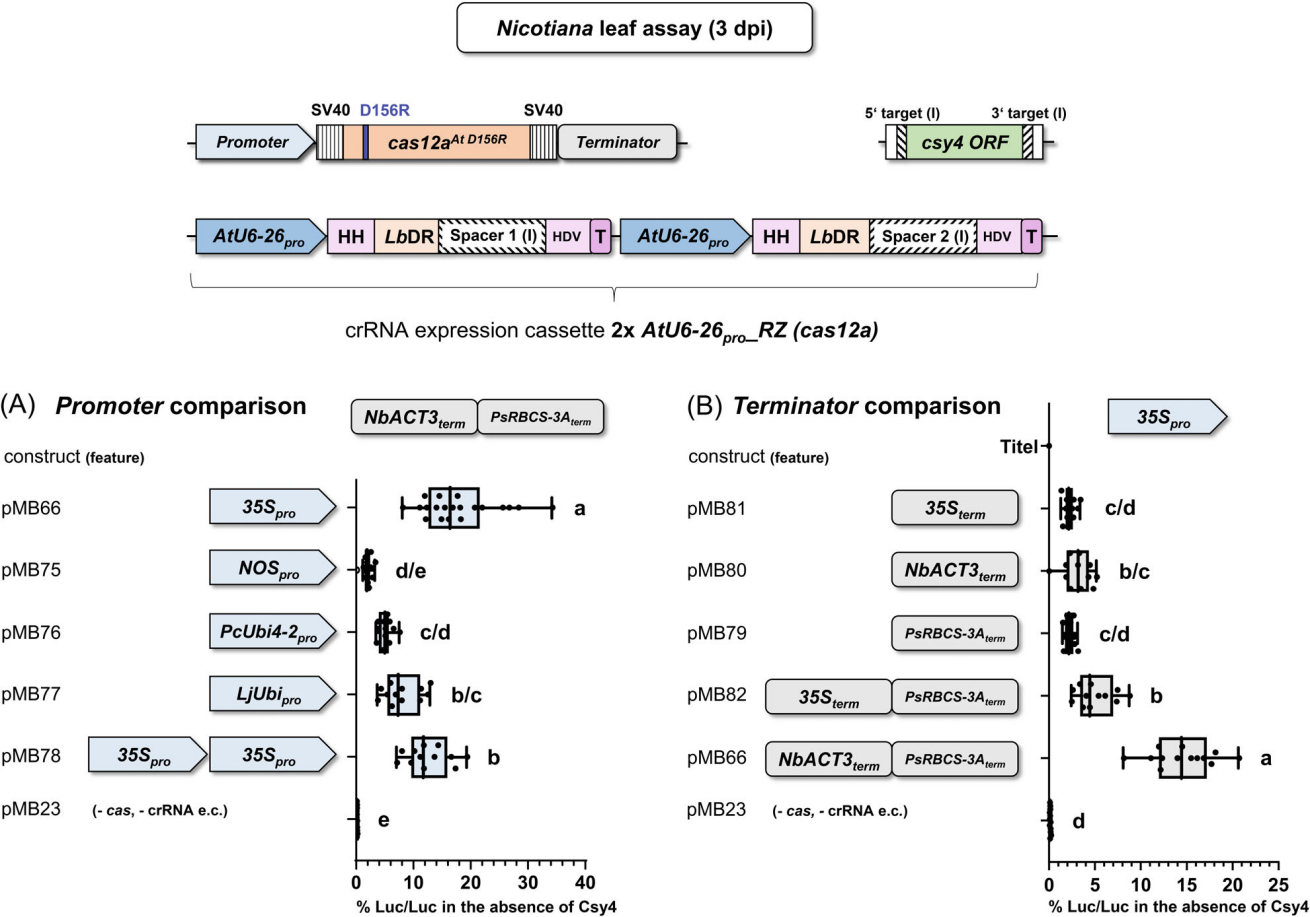
Influence of different promoters and terminators on editing efficiency. (A, B) Comparison of a series of promoter and terminator combinations controlling the expression of *cas12a* ^*At D156R*^ in the firefly luciferase assay in *N. benthamiana* leaves. Quantification of firefly luciferase activity in *N. benthamiana* leaf cells transformed with constructs including the *Csy4* cassette with Cas targets version (I). (A) Promoter comparison using *cas12a* ^*At D156R*^ flanked by the promoters as indicated and the tandem terminators *NbACT3*_*term*_ + *PsRBCS‐3A*_*term*_. Relative firefly luciferase activity was strongest in the presence of *35S*_*pro*_ (pMB66). (B) Terminator comparison using *cas12a* ^*At D156R*^ flanked by the promoter *35S*_*pro*_ and the terminator(s) as indicated. In combination with the *35Spro*, the tandem terminators *NbACT3*_*term*_ + *PsRBCS‐3A*_*term*_ led to the highest editing efficiency. See Figure S7 for additional details of the constructs used. *35S*_*pro*_, Cauliflower mosaic virus *35S RNA* gene promoter; *LjUbi*_*pro*_, *Lotus japonicus* *polyubiquitin* promoter; *NOS*_*pro*_, *Nopaline synthase* promoter; *PcUbi4‐2*_*pro*_ *; Petroselinum crispum Ubi4‐2* promoter*; NbACT3*_*term*_, *Nicotiana benthamiana (Nb) ACT3* terminator; *PsRBCS‐3A*_*term*_, *Pisum sativum (Ps) RBCS‐3A* terminator; SV40, nuclear localization sequence (NLS) of the Simian Virus 40; *cas12a* ^*At D156R*^, *A. thaliana* (*At*) codon‐usage‐adapted *cas12a* gene encoding the D156R replacement; e.c., expression cassette; % Luc/Luc, firefly luciferase activity normalized to *Renilla* luciferase activity; one‐way ANOVA followed by Tukey test was performed for the whole data set, and values with no significant difference to each other were grouped by letters (a–e). Values labeled with different letters are statistically significantly different. Values labeled with two letters are statistically not different to values with corresponding single letters. One‐way ANOVA: *P* < 0.0001.

**Figure 5:**
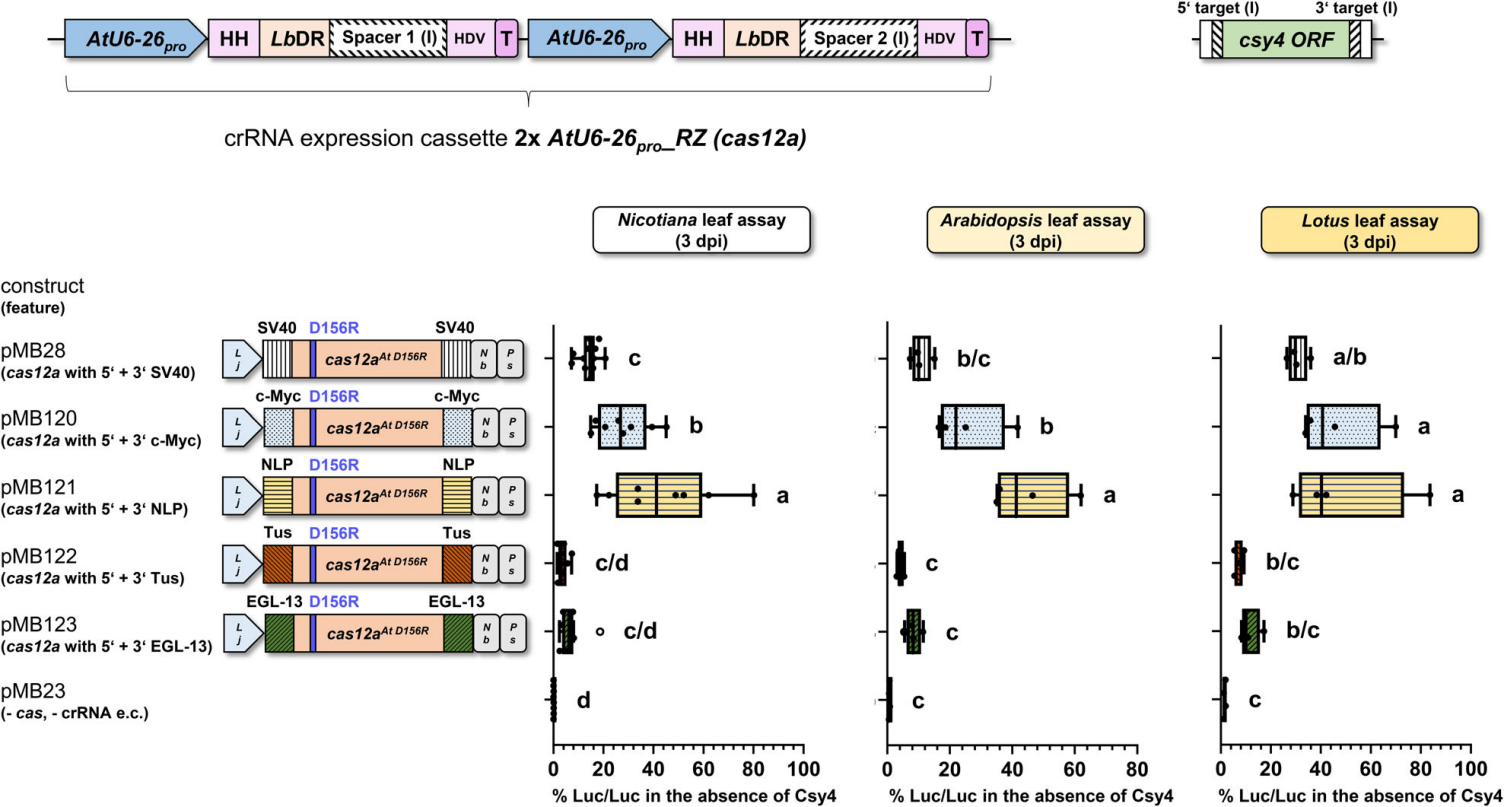
Influence of NLS variants on editing efficiency. Impact of different nuclear localization signals (NLSs) flanking Cas12a on the firefly luciferase activity. Quantification of firefly luciferase activity in *N. benthamiana*, *A. thaliana*, and *L. japonicus* leaf cells transformed with constructs including the *csy4* cassette with Cas targets version (I). Note that relative firefly luciferase activity was highest when the open reading frame of *cas12a* ^*At D156R*^ is fused to the NLSs of c‐Myc or NLP, at its 5′ and 3′ end, consistent with the nuclear localization mediated by these NLSs (Figure 7). All constructs contained the same crRNA expression cassette (*2x AtU6‐26*_*pro*_ *_2xRZ (cas12a)*) and *cas12a* expression was controlled by the same promoter (*LjUbi*_*pro*_) and terminator (*NbACT3*_*term*_ *+ PsRBCS‐3A*_*term*_) sequences (Figures S2, S5, and S8). A detailed description of the *cas12a* systems used can be found in Figure S8. We adapted all NLSs to *Lotus japonicus* codon usage; we used the *cas* system described above to compare SV40 NLS (Appendix S19) with NLP NLS (Appendix S21), c‐Myc NLS (Appendix S20), Tus NLS (Appendix S22), or EGL‐13 NLS (Appendix S23). *L* over *j* on light gray background pentagon, *Lotus japonicus* *polyubiquitin* promoter; *Nb*, *Nicotiana benthamiana ACT3* terminator; *Ps*, *Pisum sativum RBCS‐3A* terminator; *cas12a* ^*At D156R*^, *A. thaliana* (*At*) codon‐adapted *cas12a* gene encoding the D156R replacement; e.c., expression cassette; % Luc/Luc, firefly luciferase activity normalized to *Renilla* luciferase activity; one‐way ANOVA followed by Tukey test was performed for the whole data set; values with no significant difference to each other were grouped by letters (a–d). Values labeled with different letters are statistically significantly different. Values labeled with two letters are statistically not different to values with corresponding single letters. One‐way ANOVA: *P* < 0.0001.

**Figure 6:**
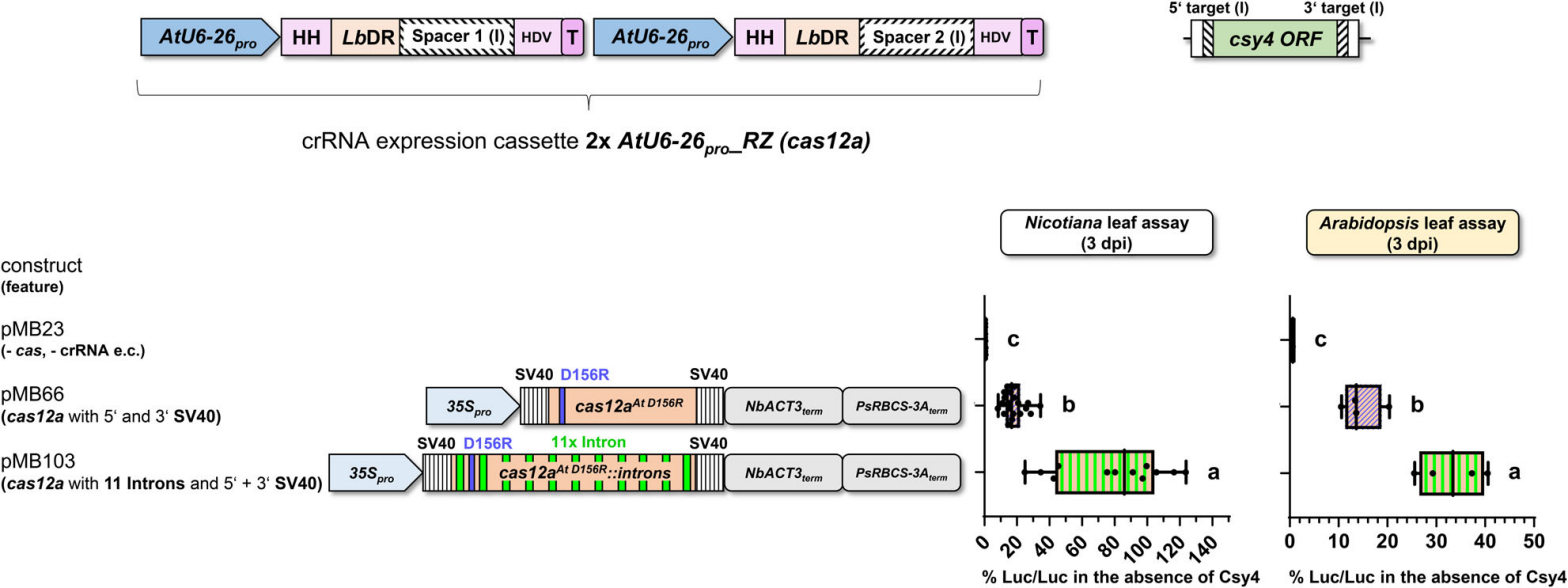
Influence of introns in *cas12a* on editing efficiency. Impact of intron presence in *cas12a* ^*At D156R*^ on genome editing efficiency determined by firefly luciferase activity. All constructs contained the same crRNA expression cassette (*2x AtU6‐26*_*pro*_ *_2xRZ* (*cas12a*)) and the same promoter (*35S*_*pro*_) and terminator (*NbACT3*_*term*_ *+ PsRBCS‐3A*_*term*_) sequences for *cas* expression (Figure 6; Figure S9). Both encoded Cas12a versions were fused to SV40 (pMB66 and pMB103) nuclear localization sequences at their N‐ and C‐termini. The *csy4* cassette with Cas targets version (I) was used as the editing target. Firefly luciferase activity was quantified in *N. benthamiana* or *A. thaliana* leaf cells. Relative firefly luciferase activity was highest with *cas12a* ^*At D156R*^ *::* *introns* and the encoded Cas12a^At D156R^ fused to the nuclear localization signal of SV40 at its N‐ and C‐terminus. A detailed description of the *cas12a* systems used can be found in Figure S9. *35S*_*pro*_, Cauliflower mosaic virus *35S RNA* gene promoter; SV40, NLS of the Simian Virus 40; NLP, NLS from nucleoplasmin of *Xenopus laevis; Ps*, *Pisum sativum*; *Nb, Nicotiana benthamiana; cas12a* ^*At D156R*^, *A. thaliana* (*At*) codon‐adapted *cas12a* gene encoding the D156R replacement*; cas12a* ^*At D156R*^ *::introns*, modified *cas12a* ^*At D156R*^ with 11 introns; the added introns are represented by green bars; e.c., expression cassette; % Luc/Luc, firefly luciferase activity normalized to *Renilla* luciferase activity; one‐way ANOVA followed by Tukey test was performed for the whole data set, and values with no significant difference to each other were grouped by letters (a–c). Values labeled with different letters are statistically significantly different. Values labeled with two letters are statistically not different to values with corresponding single letters. One‐way ANOVA: *P* < 0.0001.

For the quantitative comparison of the efficiency of *cas12a*‐based genome editing systems in plants, we used two recently published reporter switch‐on assay systems that both facilitate quantitative side‐by‐side comparisons (Figure 1A and Bircheneder et al., 2024). Both assays are based on the endonuclease gene *csy4* as target for Cas‐mediated deletion. To become a Cas‐deletion target, *csy4* ORF was artificially flanked by target sequences addressed by the guide RNAs of the Cas endonucleases under study (Appendix S24 and S25). Csy4 abolishes the translation of the reporter, which was artificially turned into a Csy4 cleavage substrate (Figure 1B). Therefore, the successful loss‐of‐function of *csy4* is detected via the consequential switch‐on activation of reporter gene expression, allowing indirect quantification of Cas‐mediated deletion events (Figure 1C). By using *csy4* as target for deletion based on Cas, these assays can be used for comparative studies on various *cas* system components, independent of, and thus minimizing the impact of, any endogenous target gene. Here we used two variants of this quantitative assay system, based on two different reporter genes: (1) *firefly luciferase*, with *Renilla luciferase* as constitutively expressed reference gene for normalization (hereafter referred to as “Luciferase assay”). This combination was used in experiments based on transient transformation of *Nicotiana* leaves, *Arabidopsis* leaves and *Lotus* leaves (Figures 2, 3, 4, 5, 6 and 8; Figures S1 and S2). Firefly luciferase expression is sensitive to Csy4 activity, the ORF encoding it is deleted by Cas editing activity (Figure 1). (2) *mCitrine* encoding a fluorescent protein as reporter gene in combination with *hygromycin phosphotransferase* as reference gene in an assay based on the stable transformation of *Lotus* calli (hereafter called “*Lotus* callus assay”) (Figure 3; Figure S1). By PCR analysis of the transformed plant tissue and Sanger sequencing of the PCR product it has been demonstrated that the loss‐of‐function effects observed are caused by full deletion (Figures S12 and S13; Bircheneder et al., 2024).

### Multiple components of *cas* systems influence editing efficiency

As expression‐enhancing effects could have a positive effect on editing efficiency, we tested and compared combinations of promoters and terminators (Ingelbrecht et al., 1989; Nagaya et al., 2010; Pérez‐González & Caro, 2018; Wang et al., 2015; Yang et al., 2009). Moreover, we tested the impact of introns, as in many eukaryotes, including plants, introns can increase the expression of transgenes (Shaul, 2017).

Cas‐induced DNA DSBs occur in the nucleus of eukaryotic cells, requiring Cas to be transported into the nucleus. As Cas proteins are of bacterial origin, they do not contain a nuclear localization signal (NLS). We therefore tested which NLSs and at what position attached to Cas12a affect the overall editing efficiency. We relied on a set of NLS variants which were investigated previously for their impact on the nuclear accumulation of NLS‐eGFP (Ray et al., 2015). Consequently, we evaluated the contribution of various components with regard to their influence on the genome editing efficiency of *Cas12a* systems.

## RESULTS

### Editing efficiency conferred by four re‐coded *cas12a* ORF versions in *N. benthamiana*, *A. thaliana*, and *L. japonicus*

The *cas12a* gene is of eubacterial origin, but the Cas12a protein is frequently expressed in plant species for genome editing purposes. To improve expression in eukaryotes, *cas12a* is typically re‐coded using eukaryotic codon usage frequency tables. However, eukaryotes also differ in their codon usage frequencies. We therefore investigated whether re‐coding of *cas12a* adopting codon usage frequencies from four different eukaryotes has an effect on the editing efficiency in different plant species. We compared four versions of the *cas12a* gene with a re‐coded ORF based on the codon usage of *Arabidopsis thaliana* (*At*), *Nicotiana benthamiana* (*Nb*), *Lotus japonicus* (*Lj*), and human (*Homo sapiens*, *Hs*). For a quantitative comparison, the luciferase assay was performed in transiently transformed leaves of *Nicotiana*, *Arabidopsis*, or *Lotus* (Figure 2). To increase the comparability, we used the same promoter and terminator sequences for *cas* expression, as well as the same crRNA expression cassette (Figure S4). In all three transiently transformed plant species, the *At* re‐coded version performed best, with at least 50% higher efficiency than the next best version (Figure 2).

### Editing efficiency of three different crRNA expression systems

For full deletion of a gene of interest, Cas12a has to mediate two DSBs; therefore, requiring at least two crRNAs for two different target sequences. Three major systems for processing crRNA have been described (Bin Moon et al., 2018; Gao et al., 2018; Li et al., 2018; Tang et al., 2017; Wang et al., 2017, 2018; Zetsche et al., 2017; Zhang et al., 2021). First, a self‐processing (SP) crRNA expression cassette controlled by a RNA polymerase III promoter (Figure S3A) (Zetsche et al., 2017). Second, the T_4_AT_6_ crRNA expression cassette (Figure S3B), including an artificial T_4_AT_6_ overhang, is described to result in an increased editing efficiency (Bin Moon et al., 2018). The third crRNA processing system expresses a gRNA flanked by 2 distinct ribozymes (2xRZ) (Figure S3C), mediating precise intramolecular RNA cleavage (Ferré‐D'Amaré & Scott, 2010; Gao et al., 2018; Gao & Zhao, 2014; Tang et al., 2017). We investigated the influence of these three different crRNA expression systems and the impact of different RNA polymerase III promoters on the editing efficiency of a *cas12a* system (Figure 3A; Figures S1 and S6). We chose *cas12a* with *A. thaliana* codon usage carrying a D156R mutation, which is known to be temperature‐tolerant (*cas12a* ^*At D156R*^) (Schindele & Puchta, 2020). Although we did not observe a significant difference in editing efficiency (Figure S5), previous work has shown that this version can reduce the potential influence of the external factor temperature on editing efficiency in stable transgenic plant lines (Schindele & Puchta, 2020).

In the *Lotus* callus assay, the 2xRZ system 2x *AtU6‐26*_*pro*_ *_2xRZ* (pMB50) led to a significantly higher editing efficiency (ratio of living fluorescent to non‐fluorescent calli; Figure 3A), revealing the potential of employing ribozymes for the preparation of the tailor‐made guide RNA.

### The impact of promoters driving crRNA production

To further investigate the impact of different RNA polymerase III promoters on editing efficiency, we employed the luciferase assay in *Nicotiana* leaves (Figure 3B; Figure S6). By using the SP system, only *AtU6‐26*_*pro*_ (pMB65; *AtU6‐26*_*pro*_ *_SP*) led to a notable increase in editing efficiency. Furthermore, the activity could be nearly tripled by switching from this SP system with *AtU6‐26*_*pro*_ to the 2xRZ system and using a double promoter system (pMB66; 2x *AtU6‐26*_*pro*_ *_2xRZ*) (Figure 3B). Taken together, the highest editing efficiency could be achieved by using the *A. thaliana* RNA polymerase III promoter *U6‐26* in a 2x *AtU6‐26*_*pro*_ *_2xRZ* system (Figure 3A,B).

### The influence of different Cas12a target sequence pairs

To evaluate whether the Cas12a target sequences have an additional influence on the editing efficiency, we compared two *csy4* cassettes with different flanking target sequence pairs (I) or (II) (Figure 3A; Figure S1; Appendix S24 and S25). No statistically relevant difference could be detected (Figure S1) suggesting that the impact of the target sequences was below detection level in this particular set‐up.

### The influence of different promoters and terminators controlling *cas12a* expression

To investigate the influence of the expression level of the *cas12a* gene on editing efficiency, we compared different promoters and terminators using the Luciferase assay in *Nicotiana* leaves. For this, we used Cas12a^At D156R^ fused at both ends with a monopartite SV40 nuclear localization sequence of SV40 protein from simian virus (Kalderon et al., 1984). To increase the comparability, we used the same crRNA expression cassette (*2x AtU6‐26*_*pro*_ *_2xRZ (cas12a)*) for all constructs (Figure S7); in the case of promoter comparison, the same tandem terminator (*NbACT3*_*term*_ + *PsRBCS‐3A*_*term*_) for *cas* expression (Figure 4A) and the same promoter sequence (*35S*_*pro*_) for *cas* expression in the case of the terminator comparison (Figure 4B).

We compared the editing efficiency of *cas* systems controlled by promoters *35S*_*pro*_, *NOS*_*pro*_, *PcUbi4‐2*_*pro*_, *LjUbi*_*pro*_, and *2x 35S*_*pro*_. The significantly highest activity was received with *35S*_*pro*_, with approximately 50% more compared with the next best performing promoter, *2x 35S*_*pro*_ (Figure 4A).

A diverse set of terminators was utilized previously for *cas12a* expression, including *35S*_*term*_ (Bernabé‐Orts et al., 2019; Xu et al., 2017) and *PsRBCS‐3A*_*term*_ (Schindele & Puchta, 2020), for which we compared their effect on *cas12a*‐based editing efficiency. Moreover, we included the terminator *NbACT3*_*term*_ into the comparison. The activity observed for all these three systems was quite low. *PsRBCS‐3A*_*term*_ and *NbACT3*_*term*_ were not statistically different from *35S*_*term*_ (Figure 4B). However, when the single terminators were replaced by either of two alternative tandem terminator versions, the activity could be increased (Figure 4B). The by far strongest activity (fivefold higher than the *35S*_*term*_) was obtained by using the tandem arrangement *NbACT3*_*term*_ *+ PsRBCS‐3A*_*term*_ (pMB66). These results indicate that using a tandem terminator is more favorable for *cas* system improvement than a single terminator. This would align with prior studies showing that using a tandem terminator leads to significantly higher transgene expression, underscoring the critical role of transcription termination in gene regulation (Luo & Chen, 2007; Yamamoto et al., 2018). In summary, we conclude that the highest editing efficiency was achieved by expressing *cas12a* under the control of the *35S*_*pro*_ along with the tandem terminator *NbACT3*_*term*_ + *PsRBCS*‐*3A*_*term*_.

### Two nuclear localization signals of SV40 flanking Cas12a are better than a single one

For editing of eukaryotic genomes, bacterial Cas endonucleases need to be transported to the nucleus. Therefore, NLSs fused to a Cas endonuclease have an impact on the transport of the systems into the nucleus and thus on the editing efficiency of the system (Grützner et al., 2021). To determine whether the position and number of NLS fusions to Cas12a have a quantitative impact on editing efficiency, we constructed *cas12a* ^*At D156R*^ with no NLS fusion, with the monopartite NLS of SV40 fused only at the 5′ end, only at the 3′ end, or at both ends. By using SV40 NLSs flanking Cas12a at both termini (pMB28) we observed three‐ to sixfold higher activity than for a single SV40 NLS at either the N‐ or C‐terminus or no NLS (Figure S2), indicating that editing efficiency is highest when the Cas12a is flanked by NLSs on both ends. This is consistent with previous studies in which the highest editing efficiency in *Arabidopsis* (Develtere et al., 2024; Grützner et al., 2021) and delivery to the nuclei of mammalian cells (Cong et al., 2013) were obtained when Cas9 was flanked by NLSs at both ends.

### Nuclear localization signals: Highest editing efficiency achieved by flanking Cas12a with NLP NLS or c‐Myc NLS

We investigated the influence of different NLS variants flanking Cas12a on the editing efficiency of a *Cas12a* system. We compared the monopartite NLS of SV40 of the simian virus (Kalderon et al., 1984), the monopartite NLS of the human transcription factor c‐Myc (Dang & Lee, 1988) and the NLS of the replication terminator protein Tus of *Escherichia coli*, which despite being a bacterial protein was shown to function as an NLS in non‐bacterial systems (Kaczmarczyk et al., 2010). Moreover, the bipartite NLS of NLP, a nucleoplasmin of *Xenopus laevis* (Dingwall et al., 1988) and the putative bipartite NLS of the EGL‐13 protein of *Caenorhabditis elegans* (Lyssenko et al., 2007) were included in the comparison. NLP NLS and SV40 NLS were already used previously to localize Cas12a to the nucleus (Schindele & Puchta, 2020; Wang et al., 2017). For this quantitative analysis, the luciferase assay was utilized in leaves of *Nicotiana*, *Arabidopsis*, and *Lotus* (Figure 5). Usage of 5′ + 3′ NLP NLSs exhibits a significant increase in activity compared with 5′ + 3′ SV40 NLSs and 5′ + 3′ c‐Myc NLSs in *Nicotiana* and *Arabidopsis*, and a slight, non‐significant trend toward higher activity in *Lotus* (Figure 5). Taken together, nuclear localization signals of c‐Myc or NLP as nuclear localization signals flanking Cas12a at both ends can be chosen for improving Cas12a systems in *Nicotiana*, *Arabidopsis*, and *Lotus*. Our data are in line with Ray et al. (2015), demonstrating that the NLS of NLP and the NLS from c‐Myc led to the highest uptake of reporter‐cargo green fluorescent protein (GFP) in the nucleus of human cells.

### Editing efficiency was greatly enhanced by inserting introns into the *cas12a* transcribed region

We generated the intronized variant *cas12a* ^*At D156R*^ *::introns* by integrating 11 introns (i) from *Arabidopsis* genes in the coding region of *cas12a* ^*At D156R*^ (Figure 6; Appendix S17). The intron sequences were based on *cas9* ^*Zm*^ *::introns* (*Zea mays* codon‐adapted *cas9* gene from *Streptococcus pyogenes*) generated by Grützner et al. (2021) (Appendix S17 and S18). The introns were evenly spaced throughout the coding sequence (CDS) of *cas12a* ^*At D156R*^, with an average distance of 200–400 nucleotides. This version led to a drastic increase in editing efficiency compared with the intron‐free *cas12a* ^*At D156R*^ both in *Nicotiana* and *Arabidopsis* leaves when Cas12a was fused with SV40 NLSs on the 5′ and 3′ end (Figure 6). This aligns with recent studies on *cas12a* intron variants, where 8 or 10 introns were introduced into the *cas12a* ^*At D156R*^ sequence (Lawrenson et al., 2022; Schindele et al., 2023). These findings are generally consistent with those reported for intronized variants of *cas9* (Grützner et al., 2021).

### NLP NLS flanking the intron version of *cas12a* greatly increases the accumulation of Cas12a protein in the nucleus of *Nicotiana* cells

**Figure 7:**
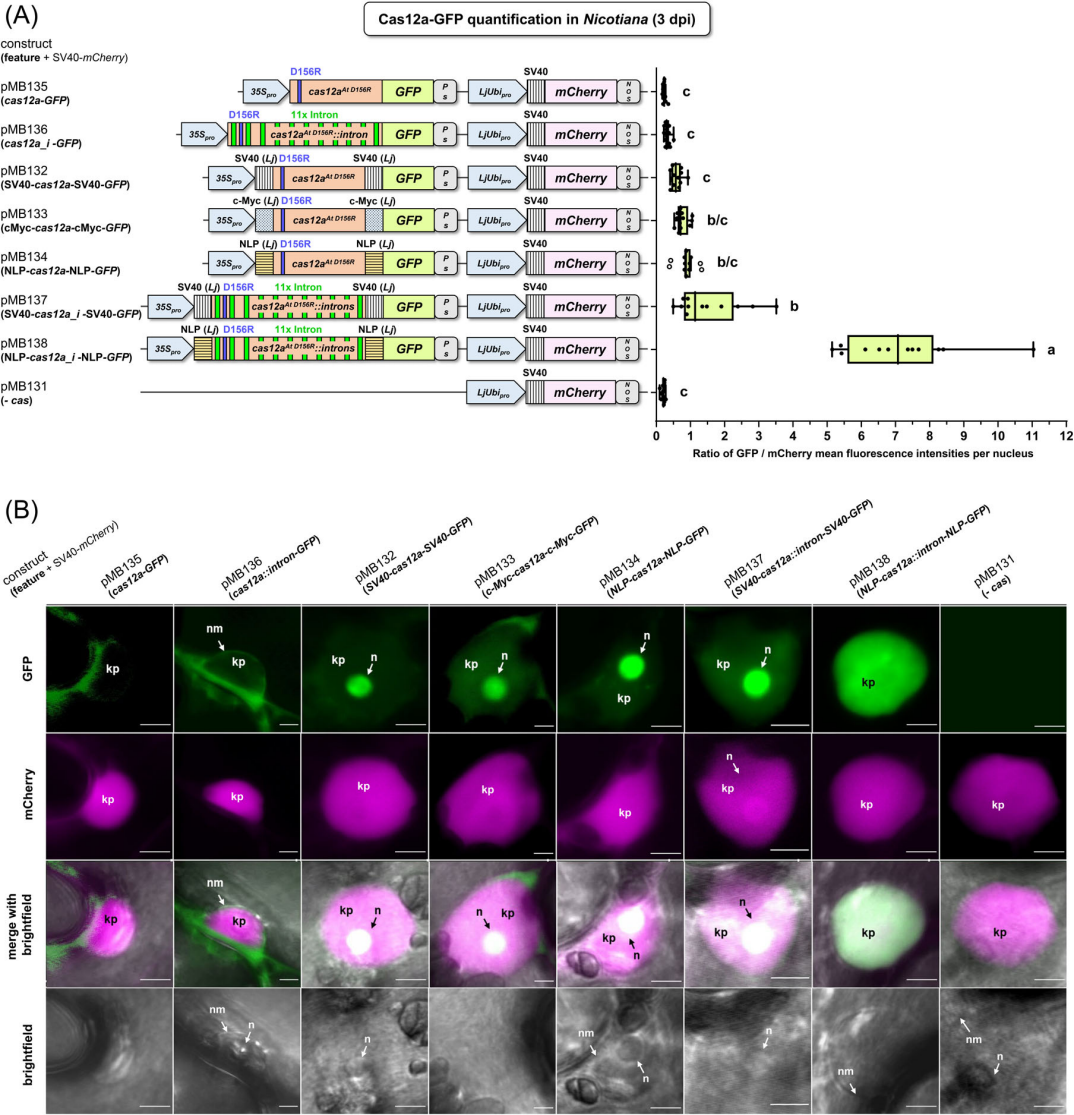
Quantitative evaluation of nuclear localization of different versions of Cas12a‐GFP fusion protein. (A, B) GFP fluorescence intensity in nuclei of *N. benthamiana* leaf cells transformed with constructs including *cas12a‐GFP* fusions normalized to mCherry fluorescence intensity. Beside Cas12a‐GFP, all analyzed constructs contained SV40‐mCherry under control of *LjUbi*_*pro*_ and *NOS*_*term*_ on the same T‐DNA for normalization of GFP fluorescence. Three days after inoculation with *A. tumefaciens*, randomly selected fluorescent nuclei of leaf epidermal cells were imaged. Per construct, 12 randomly selected nuclei were analyzed. On T‐DNAs of used constructs, *cas12a* ^*At D156R*^ (pMB132, pMB133, pMB134, and pMB135) and the intronized version *cas12a* ^*At D156R*^ *::introns* (pMB136, pMB137, and pMB138) are flanked with no or different nuclear localization sequences (NLSs). (B) Confocal laser scanning microscopy images of exemplary nuclei of *N. benthamiana* leaf cells 3 days post‐inoculation (dpi). GFP: Cas12a‐GFP variant indicated on top, mCherry: mCherry‐NLS as internal reference for nuclear localization and quantification. Note, Cas12a‐GFP, when detectable in the nucleus, accumulates mainly in a nuclear structure which could possibly be the nucleolus (n). Note that NLP‐Cas12a::i‐NLP‐GFP exhibited a very pronounced GFP fluorescence (a), which was in some cells (but not all) distributed over the entire karyoplasm, so that the potential nucleolus was no longer recognizable as an accumulation site. We adapted all NLSs to *Lotus japonicus* codon usage as indicated by (*Lj*); *35S*_*pro*_, Cauliflower mosaic virus *35S RNA* gene promoter; *LjUbi*_*pro*_, *Lotus japonicus* *polyubiquitin* promoter; *cas12a* ^*At D156R*^, *A. thaliana* (*At*) codon‐adapted *cas12a* gene encoding the D156R replacement; *cas12a* ^*At D156R*^ *::introns*, modified *cas12a* ^*At D156R*^ with 11 introns (i), the added introns are represented by green bars; Ps, *Pisum sativum RBCS‐3A* terminator; *NOS*, *NOS*_*term*_; *GFP*, gene of the green fluorescent protein; expression of *cas12a* is controlled by *35S*_*pro*_ and terminator *PsRBCS‐3A*_*term*_; expression of *mCherry* is controlled by *LjUbi*_*pro*_ and terminator *NOS*_*term*_; n, structure within nucleus, likely nucleolus; kp, karyoplasm; nm, nuclear membrane; scale bars represent 5 μm; one‐way ANOVA followed by Tukey test was performed for the whole data set, and values with no significant difference to each other were grouped by letters (a–c). Values labeled with different letters are statistically significantly different. Values labeled with two letters are statistically not different to values with corresponding single letters. One‐way ANOVA: *P* < 0.0001.

To investigate the influence of NLSs and introns on the nuclear accumulation of Cas12a proteins, we fused a series of Cas12a^At D156R^ variants flanked by different NLS versions (or as control flanked by none) with the green fluorescent protein (GFP) at the C‐terminus (Figure 7). Upon synthesis of these Cas12a^At D156R^‐GFP fusions in *N. benthamiana* leaf cells, fluorescent nuclei were analyzed by confocal laser scanning microscopy (CLSM) (Figure 7). By using the intron‐free *cas12a* version without NLS (pMB135), no intranuclear GFP fluorescence could be detected, similar to the negative control lacking *cas12a‐GFP* (pMB131). A weak, perhaps intranuclear, GFP signal could be detected by using the intron version *cas12a* ^*At D156R*^ *::introns* (pMB136), although fluorescence bleed‐through from the surrounding cytoplasm could not be excluded.

When the intron‐free *cas12a* version was flanked by sequences of SV40 NLSs, c‐Myc NLSs, or NLP NLSs, a slight increase in GFP fluorescence intensity could be observed, confirming the predicted role of NLSs for Cas12a uptake into the nucleus. When the intron‐free *cas12a* version was replaced by the intronized version *cas12a* ^*At D156R*^ *::introns*, signal intensity could be further increased. The most intensive effect was achieved by flanking Cas12a^At D156R^::introns at both the N‐ and the C‐terminus with NLP (pMB138), with a sevenfold fluorescence intensity increase compared with the intron‐free version with NLP (pMB134). When the localization of Cas12a‐GFP was examined in *Nicotiana* leaf cells, all versions of Cas12a with NLSs were observed in the karyoplasm, mainly accumulating in an intranuclear round structure that possibly represents the nucleolus (Figure 7B) indicating that the analysed fusion was primarily subjected to nucleolus‐ and not to nucleus‐localization. When using the intronized version of *cas12a* flanked by sequences of NLP NLSs (pMB138), a very high GFP fluorescence was measured (Figure 7A). Besides the accumulation in the potential nucleolus mentioned above (observed in 10 out of 12 nuclei), in approximately 16% of the analyzed nuclei (2 out of 12) the GFP fluorescence was additionally distributed over the entire karyoplasm with high intensity, so that the potential nucleolus was no longer recognizable as an accumulation site (Figure 7B). This signal distribution pattern was not observed in any other construct analyzed within this study. Taken together, we could confirm that NLSs flanking Cas12a at the N‐ and C‐terminus can mediate nuclear uptake of Cas12a and that the nucleolus appears to be the primary accumulation site for Cas12a:GFP fusions. In particular, NLP was key for achieving high Cas12a uptake. This effect could be further enhanced by using constructs with intronized *cas12a* variants.

### *cas12a::introns* can compete with *cas9::introns* in terms of editing efficiency

**Figure 8:**
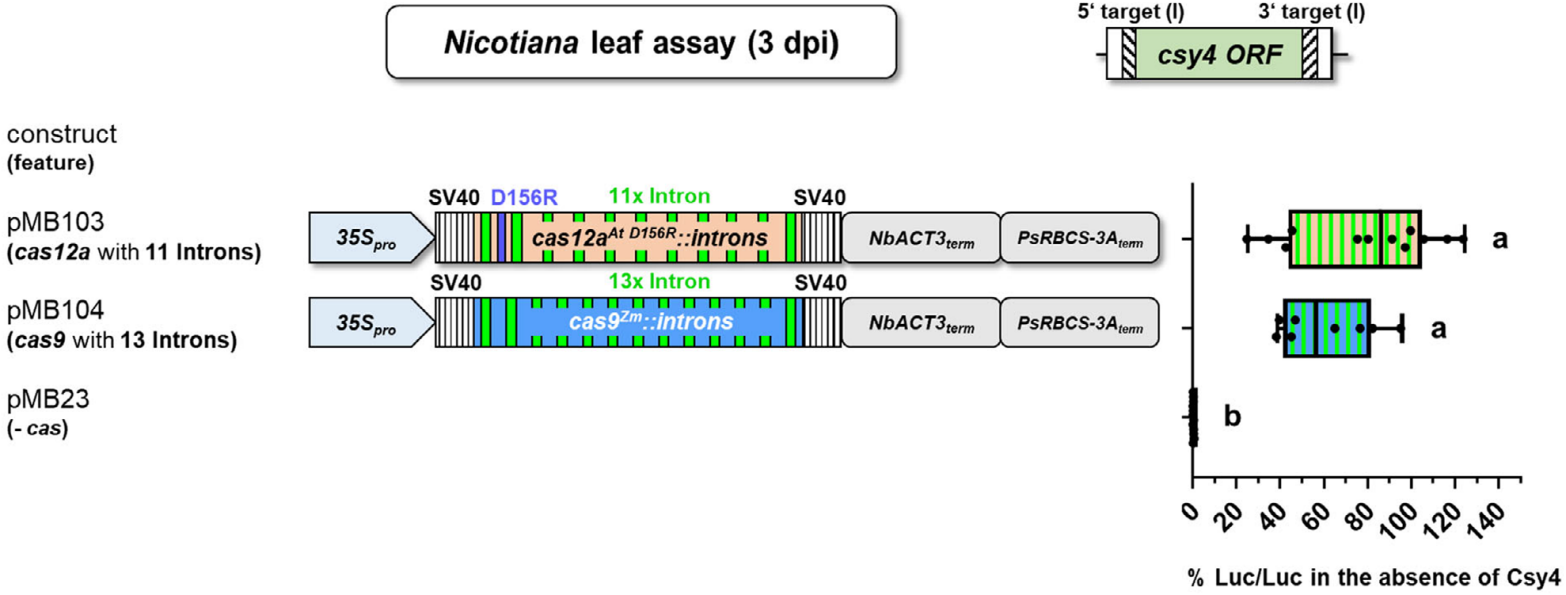
Comparison of a *cas12a* and a *cas9* system. Efficiency of a *cas12a* and a comparable *cas9* system in the firefly luciferase assay. Quantification of firefly luciferase activity in *N. benthamiana* leaf disc cells transformed with constructs including the *csy4* cassette with Cas targets version (I). % Luc/Luc, firefly luciferase activity normalized to *Renilla* luciferase activity. Significance of luciferase activity from pMB104 was tested against pMB103. Note, the *cas12a* system led to a slightly but not significantly higher firefly luciferase activity than the *cas9* system. This difference may be bigger, as some of the pMB103 data points was already above the 100% mark. A detailed description of the *cas* systems used can be found in Figure S10. *35Spro*, Cauliflower mosaic virus *35S RNA* gene promoter; SV40, nuclear localization signal of the Simian Virus 40; *Ps*, *Pisum sativum*; *Nb*, *Nicotiana benthamiana; cas12a* ^*At D156R*^ *::introns*, *A. thaliana* (*At*) codon‐adapted *cas12a* gene encoding the D156R replacement and 11 introns; *cas9* ^*Zm*^ *::introns*, *Zea mays* codon‐adapted *cas9* gene from *Streptococcus pyogenes* (*Sp*) with added 13 introns (i); the added introns are represented by green bars, *e*xpression of the *cas* versions used is controlled by the promoter *35S*_*pro*_ and the tandem terminators *NbACT3*_*term*_ + *PsRBCS‐3A*_*term*_. One‐way ANOVA followed by Tukey test was performed for the whole data set; values with no significant difference to each other were grouped by letters (a, b). Values labeled with different letters are statistically significantly different. Values labeled with two letters are statistically not different to values with corresponding single letters. One‐way ANOVA: *P* < 0.0001.

We compared the performance of intronized *cas12a* ^*At D156R*^ *::introns* with a published *cas9* intron variant (*cas9* ^*Zm*^ *::introns*) (Grützner et al., 2021). To increase the comparability, we used the same promoter and terminator sequences for *cas* expression and both Cas proteins were fused at both ends with a SV40 NLS (Figure 8; Figure S10). Furthermore, we could use the identical target cassette, as the artificial 5′ and 3′ target sequences flanking the ORF of *csy4* both contained a *cas9* PAM as well as a *cas12a* PAM (Bircheneder et al., 2024). The expression of the “*2x AtU6‐26*_*pro*_ *_2xRZ (cas12a)*” crRNA expression cassette (Figure S10) and the *cas9* sgRNA expression cassette (Figure S10; Appendix S9) was driven by the *A. thaliana* RNA polymerase III promoter *U6‐26*. However, two differences between the compared systems should be highlighted: (1) *cas12a* ^*At D156R*^ *::introns* was re‐coded based on *Arabidopsis* codon usage, while *cas9* ^*Zm*^ *::introns*, employs *Zea mays* codon usage. (2) The sequences of *cas9* ^*Zm*^ *::introns* and *cas12a* ^*At D156R*^ *::introns* contain 13 and 11 introns, respectively. We observed no significant difference in editing efficiency between these two *cas* systems (Figure 8).

## DISCUSSION

### Multiple variables enable improvement of genome editing tools in plants

To improve the editing efficiency of *cas12a*‐based systems, we compared *cas12a* re‐coded versions, the impact of promoters and terminators controlling *cas12a* expression, and the addition of introns into the *cas12a* ORF. Importantly, we also investigated the influence of modifications of the crRNA expression cassette. Our results revealed significant effects across the features analyzed. The strongest differences were obtained by codon usage (with a difference of approximately sevenfold between strongest and weakest performer; Figure 2), by the *cas12a* promoter (difference of around eightfold between strongest *35S*_*pro*_ and weakest *NOS*_*pro*_; Figure 4A), by the terminator (difference of around fivefold between strongest tandem terminator *NbACT3:PsRBC‐3A*_*term*_ and the weakest *35S*_*term*_; Figure 4B), and by Cas12a fusions with eukaryotic NLS (difference of around 10‐fold between weakest NLS of Tus from *E. coli* and strongest NLS of NLP; Figure 5). Perhaps, the strongest increase was observed upon the addition of 11 introns into *cas12a*, leading to an approximately fivefold difference between otherwise identical constructs (pMB66 vs. pMB103; Figure 6). This effect is likely due to enhanced gene expression (Shaul, 2017). Additionally, the presence of introns may help to mitigate transgene silencing (Christie et al., 2011). Our data thus reveal a high potential for improving *cas12a*‐based editing efficiency by adjustments to all of these relevant features. However, as we could only test a small number of variants for each of the features, even better‐performing constructs are possible. So far, our construct designs have been used by three different laboratories to edit at least 14 *Lotus* genes, resulting in the successful production of corresponding *L. japonicus* mutant lines. Among these, we generated a line carrying a deletion of the entire coding region plus introns of the *Lotus japonicus NIN* (*Nodule Inception*) gene. NIN is a transcription factor essential for root nodule symbiosis (Schauser et al., 1999). While the genetic and phenotypic characterization of these lines is outside the scope of this work, their successful generation demonstrates that our *cas12a* systems are in principle applicable for the production of genome‐edited plants.

### Impact of the crRNA expression cassette

To maximize the editing efficiency of the *cas12a* system, it is crucial to ensure adequate expression of both *cas* genes and crRNA. Research on *cas9* systems has demonstrated that maintaining an optimal ratio between Cas9 proteins and sgRNAs is vital for efficient genome editing (Dahlman et al., 2015). Higher Cas9 levels have been associated with diminished specificity and lower efficiency at target sites (Fu et al., 2013). An excess of Cas proteins without sufficient sgRNA can result in non‐productive or off‐target interactions, thereby diminishing editing efficiency (Dahlman et al., 2015). Overexpression of *cas9* has been associated with cellular toxicity, largely due to increased DNA breaks and non‐specific interactions. The resulting stress response can activate repair mechanisms that interfere with the intended genetic modifications (Enache et al., 2020).

The original SP crRNA expression system described by Zetsche et al. (2015) for use with Cas12 has undergone extensive modifications in the scientific community (Gao et al., 2018; Tang et al., 2017; Wang et al., 2017; Zhang et al., 2021). These alterations primarily aim to allow the simultaneous expression of multiple crRNAs and enhance overall editing efficiency.

We observed that the promoters regulating the crRNA expression cassette and their arrangement are key factors. Editing efficiency in the ribozyme system 2xRZ was improved when each crRNA was controlled by individual promoters, rather than a single promoter driving both crRNAs (Figure 3A). However, using the RNA polymerase II promoter *LjUbi*_*pro*_ instead of the RNA polymerase III promoter *LjU6*_*pro*_ led to no significant difference in editing efficiency (Figure 3A). This contrasts with previous findings, where a RNA polymerase II promoter, *ZmUbi*_*pro*_, regulating a ribozyme system achieved the highest editing efficiency (Zhang et al., 2021). However, this variation may be attributed to the use of promoters of different plant systems. Additionally, in contrast to our work, Zhang et al. (2021) also incorporated a poly‐T sequence before the terminator of all compared constructs. The importance of promoter choice for editing efficiency was also underlined by our finding that significant improvement of the editing efficiency could be achieved by replacing different RNA polymerase III promoters with the RNA Polymerase III *AtU6‐26*_*pro*_ (Figure 3A,B). The 2xRZ system was observed to be the best performing system when using the *Lotus* callus assay (Figure 3A) and the luciferase assay in *N. benthamiana* (Figure 3B).

We exclusively employed constitutively active promoters. Since it has been shown that germline‐specific promoters can increase the editing efficiency for Cas9‐based stable knockouts, it would be interesting to include germline‐specific promoters in attempts to increase Cas12a‐based genome editing efficiency even further  (Ursache et al., 2021; Wang et al., 2015).

### NLS dependent Cas12a accumulation in the nucleus

As genome editing is happening in the nucleus, we examined the nuclear accumulation of Cas12a‐GFP variants (Figure 7). We identified a correlation between the localization of Cas12a flanked by different NLSs in the nucleus and the editing efficiency of the associated *cas* system (Figures 5 and 7).

However, the condensed fluorescence in an intranuclear sub‐compartment (Figure 7B), potentially the nucleolus, needs attention, as it potentially removes a large percentage of the Cas12a from the location of the genomic DNA, the nucleoplasm. What drives this local accumulation remains to be determined. The intrinsic affinity of Cas12a to RNA might play a role, as ribosome assembly including ribosomal RNA is concentrated in the nucleolus. If this sequestration of Cas12a into a subcompartment of the nucleus is indeed a general phenomenon, it should be taken into account when aiming for balanced expression levels between Cas12a and the crRNAs in the nucleoplasm.

## EXPERIMENTAL PROCEDURES

### Constructs and *cas12a* variants

A detailed description of constructs is provided in Table S2. Constructs were generated by the Golden Gate cloning system according to Binder et al. (2014) and per the procedure described in Bircheneder et al. (2024). The Cas12a protein sequence from *Lachnospiraceae bacterium* ND2006 (Zetsche et al., 2015) was used as a template for independent codon usage adaptations. A plasmid containing *cas12a* ^*At D156R*^ sequence (Schindele & Puchta, 2020) was provided by H. Puchta, Botanical Institute, Karlsruhe Institute of Technology, Karlsruhe, Germany. *cas12a* ^*At*^ was generated from *cas12a* ^*At D156R*^ by removing the D156R mutation by PCR‐based cloning (for primers see Table S3). The *cas9* ^*Zm*^ *::introns* sequence (Grützner et al., 2021) was provided by S. Marillonnet and the *cas12a* ^*Nb*^ sequence was provided by T. Schreiber, both Leibniz Institute of Plant Biochemistry, Halle (Saale), Germany. The *cas12a* ^*Hs*^ sequence was derived from plasmid pY016 (pcDNA3.1‐hLbCpf1) (Addgene plasmid #69988, Zetsche et al., 2015). To generate *cas12a* ^*Lj*^, the *cas12a* gene sequence was adapted to the codon usage specific to *Lotus japonicus* based on the Cas12a amino acid sequence using the Eurofins MWG software. The sequence *cas12a* ^*At D156R*^ *::introns* was designed following a similar approach to a recently described protocol creating *cas9* ^*Zm*^ *::introns* (Grützner et al., 2021). A total of 11 introns (Appendix S17 and S18) were integrated into the sequence *cas12a* ^*At D156R*^ (for primers see Table S3). For optimizing the splicing efficiency, the NetGene2 intron splice site prediction tool (https://services.healthtech.dtu.dk/services/NetGene2‐2.42/) (Hebsgaard et al., 1996) was used to determine the order of the intron sequences used. Level III plasmids can be ordered from the European Plasmid Repository.

### crRNA expression cassette assembly

To introduce spacer sequences (Table S4) into the crRNA and sgRNA expression cassettes (for annotated sequences see Appendix S1, S2, S3, S4, S5, S6, S7, S8, and S9), it was proceeded as described in Bircheneder et al. (2024). For the corresponding general oligonucleotide template, see Appendix S10, S11, S12, S13, S14, and S15. For constructs used, see Table S2.

### Generation of transgenic *Lotus japonicus* calli

For performance of the assay with fluorescent protein reporter, transgenic *Lotus japonicus* ecotype Gifu (wild‐type, accession B‐129) (Handberg & Stougaard, 1992) was used. The *Agrobacterium tumefaciens*‐mediated transformation of *L. japonicus* cells was based on a published procedure (Tirichine et al., 2005) with modifications as described in Bircheneder et al. (2024).

### Cultivation and transient transformation of *N. benthamiana*, *A. thaliana*, and *L. japonicus* leaf cells

Cultivation of *N. benthamiana* and infiltration of leaves with *A. tumefaciens* AGL1 was performed as previously described (Cerri et al., 2012), but with modifications described in Bircheneder et al. (2024).

For direct comparison of the same *cas* system in different plants (Figures 2, 5, and 6) a uniform transformation protocol described by Zhang et al. (2020) was applied and tested (Figure S11) with minor modifications for *Lotus japonicus* ecotype Gifu (wild‐type, accession B‐129) (Handberg & Stougaard, 1992) and *A. thaliana* ecotype Columbia‐0 (Col‐0). *Arabidopsis* plants were grown in a greenhouse for 14 days under a 16‐h photoperiod. Preparation and cultivation of *Lotus japonicus* were performed as previously described (Gong et al., 2022) with the modification of transferring the seedlings after 5 days in the phytochamber to B5 medium with vitamins and sucrose (20 g/L) (Stiller et al., 1997), solidified with 0.8% Bacto™ agar (Becton Dickinson and Co.), followed by 7 days under the same light conditions.

For transient transformation of plant leaf cells, *A. tumefaciens* strain AGL1 (Lazo et al., 1991) was transformed with desired plasmids. Plant leaf cells were infiltrated with *A. tumefaciens* as previously described (Zhang et al., 2020). The same infiltration method was also applied for *L. japonicus* with minor modifications. Infiltration buffer contained Silwet L‐77 in different concentrations (*A. thaliana*: 0.005%; *L. japonicus*: 0.001%). For *A. thaliana*, the two largest leaves and for *L. japonicus* one leaf (comprising three leaflets each) per plant were chosen, and the infiltration solution was infiltrated into the underside of the plant leaves with a 1 mL plastic syringe. After infiltration, the leaves were dried in the light for 1 h and kept in the dark for 24 h at 22°C. Subsequently, the *A. thaliana* plants were moved to the greenhouse under a 16 h/8 h light/dark regime for 48 h, and the *L. japonicus* plants were transferred to square plates containing B5 medium with vitamins, solidified with 0.8% Bacto™ agar (Becton Dickinson and Co.). The plates were sealed with parafilm and placed in a climate chamber (MLR‐352H‐PE, Panasonic) at 22°C under a 16 h/8 h light/dark regime (50 μmol m^−2^ sec^−1^) for 48 h.

### Quantification of luciferase activity

For the quantification of luciferase reporter gene expression and normalization against *Renilla luciferase* reference gene in *Nicotiana* leaves, the Dual‐Luciferase® Reporter Assay System (Promega, E1910, www.promega.de) was applied with modifications described in Bircheneder et al. (2024).

For the quantification of luciferase reporter gene expression in *A. thaliana*, four leaves of different plants were pooled (approximately 150 mg of tissue) and in the case of *L. japonicus*, eight leaves (comprising three leaflets each) of different plants were pooled (approximately 150 mg of tissue). In total, four samples of pooled leaves of *L. japonicus* and *A. thaliana* were analyzed in at least two independent experiments. The plant material was immediately frozen in liquid nitrogen after harvesting and kept frozen. The frozen samples were ground using a mortar and pestle.

Firefly luciferase activity in the complete absence of Csy4 (‐ *csy4*) represents the maximum value that can be achieved by deletion of *csy4* mediated by Cas. Therefore, the median of the firefly luciferase activity normalized against reference gene *Renilla luciferase* in leaf cells transformed with control lacking a *cas* system and *csy4* is set as 100% (pMB24; ‐ *cas*, ‐ *csy4*).

### Quantification of fluorescence in the *Lotus* callus assay

Quantification of fluorescence in the *Lotus* callus assay and selection against *hygromycine phosphotransferase II* was performed as described by Bircheneder et al. (2024).

### Confirmation of full deletions of *csy4* ORF by Cas

To confirm full deletion of the ORF of *csy4* (Δ*csy4*) by PCR, primer pair pro_FW/term_Rev (Table S3), flanking the 5′ and 3′ target sequences within the *csy4* expression cassette (Appendix S24 and S25) was used with genomic DNA as template, as described in Bircheneder et al. (2024). Successful deletion was detected by the presence of bands equivalent in size to Δ*csy4* bands (Figure S12). Constructs harboring *csy4* but lacking a *cas* system (‐ *cas*; pMB20, pMB21, and pMB23) served as controls (Figure S12). Constructs pMB20 (with target sequence versions (I)) and pMB21 (with target sequence versions (II)) contain *hptII* reference gene and *mCitrine* reporter gene for *Lotus* callus assay. Construct pMB23 contains *Renilla luciferase* reference gene and *Firefly luciferase* reporter gene for *Nicotiana* leaf assay. To confirm that the bands derived from the loss of the ORF of *csy4*, they were cut out of the gel, purified from agarose and buffer, and ligated into Golden Gate backbone BB3. The plasmid was amplified in *E. coli*, and the insert was sequenced by Sanger sequencing using the primer pro_FW (Table S3; Figure S13).

### Microscopy

For the quantification of Cas12a‐GFP in the nucleus of *Nicotiana* leaf cells, confocal laser scanning microscopy (CLSM) was performed with an upright Leica TCS SP5 confocal laser scanning microscope. Randomly selected fluorescent nuclei of *N. benthamiana* leaf epidermal cells were imaged with an HCX IRAPO L25x/0.95 water objective. For image acquisition, the resolution was set to 1024 × 1024 pixels at the speed of 400 Hz and the frame average to 4. Section thickness was 1.47 μm. Pinhole was 1 PAU. Using the argon laser at 20% power, GFP was excited with the 488 nm laser line and detected at 500–530 nm, and mCherry was excited with a diode‐pumped solid‐state (DPSS) laser at 561 nm and detected at 580–620 nm at 20% power. For multicolor imaging, the frame sequential scan mode was used. The region of interest for image analysis was fully located within the nucleus, including the structure probably representing the nucleolus. Images were processed, and the mean fluorescence level within a single ROI was determined with ImageJ (Schindelin et al., 2012; Schneider et al., 2012).

### Data visualization and statistical analysis

Statistical analysis of data were performed using GraphPad Prism version 9.5.1 for Windows (GraphPad Software, San Diego, California USA, www.graphpad.com). The same software was used for data visualization. All data were visualized in a Tukey box plot. Bold black line, median; box, interquartile range (IQR); whiskers, lowest/highest data point within 1.5 times the IQR.

## AUTHOR CONTRIBUTIONS

Plasmid design and construction, quantitative performance tests in plants as well as all other experimental work described were performed by Martin Bircheneder. Martin Parniske conceived the research project and edited the manuscript; which was written by Martin Bircheneder.

## CONFLICT OF INTEREST

There are no financial conflicts of interest to disclose.

## Supporting information


**Figure S1.** Influence of different crRNA expression cassettes on the editing efficiency of a *cas12a* system.
**Figure S2.** Influence of NLS presence flanking Cas12a on the editing efficiency of a *cas12a* system.
**Figure S3.** Major systems for crRNA processing in multiplex genome editing.
**Figure S4.** Cas systems used for the comparison of re‐coded *cas12a* versions (related to Figure 2).
**Figure S5.** *cas* systems used to evaluate the influence of the D156R mutation in *cas12a* ^*At D156R*^ (related to Figure 2).
**Figure S6.** *cas* systems used to evaluate different crRNA expression cassettes (related to Figure 3).
**Figure S7.** *cas* systems used to evaluate different promoters and terminators (related to Figure 4).
**Figure S8.** *cas* systems used to evaluate NLS variants (related to Figure 5).
**Figure S9.** *cas* systems used to evaluate influence of intron presence in *cas12a* (related to Figure 6).
**Figure S10.** *cas12a*‐ and *cas9* system used for side‐by‐side comparison (related to Figure 8).
**Figure S11.** Application and testing of the transient transformation method.
**Figure S12.** *cas* systems used in this study are active in plant cells (I).
**Figure S13.** *cas* systems used in this study are active in plant cells (II).
**Figure S14.** *cas* systems used in this study were not active in *Agrobacterium*.
**Table S1.** Abbreviations used for tables and appendixes in supplement.
**Table S2.** Golden Gate LII and LIII plasmids.
**Table S3.** Primers, gene strands and oligonucleotides used in this study.
**Table S4.** Spacer sequences used in this study.
**Appendix S1.** crRNA expression cassette *LjUbi*_*pro*_ *_RZ (cas12a)*.
**Appendix S2.** crRNA expression cassette *LjUbi*_*pro*_ *_ T*_*4*_ *AT*_*6*_ *(cas12a)*.
**Appendix S3.** crRNA expression cassette *LjU6*_*pro*_ *_RZ (cas12a)*.
**Appendix S4.** crRNA expression cassette *2x LjU6*_*pro*_ *_2xRZ (cas12a)*.
**Appendix S5.** crRNA expression cassette *MtU6*_*pro*_ *_SP (cas12a)*.
**Appendix S6.** crRNA expression cassette *AtU6MoClo*_*pro*_ *_SP (cas12a)*.
**Appendix S7.** crRNA expression cassette *AtU6‐1*_*pro*_ *_SP (cas12a)*.
**Appendix S8.** crRNA expression cassette *AtU6‐26*_*pro*_ *_SP (cas12a)*.
**Appendix S9.** sgRNA expression cassette *2x AtU6‐26*_*pro*_ *(cas9)*.
**Appendix S10.** Oligonucleotide with Spacer 1 and Spacer 2 templates for cloning into the crRNA expression cassettes *LjU6*_*pro*_ *_SP (cas12a)*, *MtU6*_*pro*_ *_SP (cas12a)*, *AtU6MoClo*_*pro*_ *_SP (cas12a)*, *AtU6‐1*_*pro*_ *_SP (cas12a)* and *AtU6‐26*_*pro*_ *_SP (cas12a)*.
**Appendix S11.** Oligonucleotide with Spacer 1 template for cloning into the crRNA expression cassettes *LjUbi*_*pro*_ *_RZ (cas12a)*, *LjU6*_*pro*_ *_RZ (cas12a)*, *2x LjU6*_*pro*_ *_2xRZ (cas12a)* and *2x AtU6‐26*_*pro*_ *_2xRZ (cas12a)*.
**Appendix S12.** Oligonucleotide with Spacer 2 template for cloning into the crRNA expression cassettes *LjUbi*_*pro*_ *_RZ (cas12a)*, *LjU6*_*pro*_ *_RZ (cas12a)*, *2x LjU6_2xRZ (cas12a)* and *2x AtU6‐26*_*pro*_ *_2xRZ (cas12a)*.
**Appendix S13.** Oligonucleotide with Spacer 1 and Spacer 2 templates for cloning into the crRNA expression cassette *LjUbi*_*pro*_ *_T*_*4*_ *AT*_*6*_ *(cas12a)*.
**Appendix S14.** Oligonucleotide with Spacer 1 template for cloning into the sgRNA expression cassette *2x AtU6‐26*_*pro*_ *(cas9)*.
**Appendix S15.** Oligonucleotide with Spacer 2 template for cloning into the sgRNA expression cassette *2x AtU6‐26*_*pro*_ *(cas9)*.
**Appendix S16.** Coding sequence of *cas12a* ^*Lj*^.
**Appendix S17.** Open reading frame of *cas12a* ^*At D156R*^ *::introns*.
**Appendix S18.** Reference of the intron sequences in *cas12a* ^*At D156R*^ *::introns*.
**Appendix S19.** Sequence of the *Lotus japonicus* codon‐adapted SV40 NLS region.
**Appendix S20.** Sequence of the *Lotus japonicus* codon‐adapted c‐Myc NLS region.
**Appendix S21.** Sequence of the *Lotus japonicus* codon‐adapted NLP NLS region.
**Appendix S22.** Sequence of the *Lotus japonicus* codon‐adapted Tus NLS region.
**Appendix S23.** Sequence of the *Lotus japonicus* codon‐adapted EGL‐13 NLS region.
**Appendix S24.** ORF of *csy4* with flanking 5′ and 3′ targets in *csy4* expression cassette version (I).
**Appendix S25.** ORF of c*sy4* with flanking 5′ and 3′ targets in *csy4* expression cassette version (II).

**Data S1.** Sequences of plasmids. RAR archive of annotated maps of plasmids (Genbank format).

## Data Availability

The data that supports the findings of this study are available in the supplementary material of this article. Level III plasmids used in this work have been deposited at the European Plasmid Repository.
