# Supplementary material for: Engineering and comparison of cas12a‐based genome editing systems in plants
Source: Plant J. 2025 Sep 2;123(5):e70410. doi: 10.1111/tpj.70410 (PMC12404786; doi:10.1111/tpj.70410)
Supplement: Supplementary file 1 — Figure S1. Influence of different crRNA expression cassettes on the editing efficiency of a cas12a system. Figure S2. Influence of NLS presence flanking Cas12a on the editing efficiency of a cas12a system. Figure S3. Major systems for crRNA processing in multiplex genome editing. Figure S4. Cas systems used for the comparison of re‐coded cas12a versions (related to Figure 2). Figure S5. cas systems used to evaluate the influence of the D156R mutation in cas12a At D156R (related to Figure 2). Figure S6. cas systems used to evaluate different crRNA expression cassettes (related to Figure 3). Figure S7. cas systems used to evaluate different promoters and terminators (related to Figure 4). Figure S8. cas systems used to evaluate NLS variants (related to Figure 5). Figure S9. cas systems used to evaluate influence of intron presence in cas12a (related to Figure 6). Figure S10. cas12a‐ and cas9 system used for side‐by‐side comparison (related to Figure 8). Figure S11. Application and testing of the transient transformation method. Figure S12. cas systems used in this study are active in plant cells (I). Figure S13. cas systems used in this study are active in plant cells (II). Figure S14. cas systems used in this study were not active in Agrobacterium. Table S1. Abbreviations used for tables and appendixes in supplement. Table S2. Golden Gate LII and LIII plasmids. Table S3. Primers, gene strands and oligonucleotides used in this study. Table S4. Spacer sequences used in this study. Appendix S1. crRNA expression cassette LjUbi pro _RZ (cas12a). Appendix S2. crRNA expression cassette LjUbi pro _ T 4 AT 6 (cas12a). Appendix S3. crRNA expression cassette LjU6 pro _RZ (cas12a). Appendix S4. crRNA expression cassette 2x LjU6 pro _2xRZ (cas12a). Appendix S5. crRNA expression cassette MtU6 pro _SP (cas12a). Appendix S6. crRNA expression cassette AtU6MoClo pro _SP (cas12a). Appendix S7. crRNA expression cassette AtU6‐1 pro _SP (cas12a). Appendix S8. crRNA expression cassette AtU [file TPJ-123-0-s001.docx]

**Supporting information Bircheneder et al. (2025)**

The following supporting data is available for this article:

**Figure S1** Influence of different crRNA expression cassettes on the editing efficiency of a *cas12a* system.

**Figure S2** Influence of NLS presence flanking Cas12a on the editing efficiency of a *cas12a* system.

**Figure S3** Major systems for crRNA processing in multiplex genome editing

**Figure S4** *cas* systems used for the comparison of recoded *cas12a* versions (related to Figure 2).

**Figure S5** *cas* systems used to evaluate the influence of the D156R mutation in *cas12a^At D156R^* (related to Figure 2).

**Figure S6** *cas* systems used to evaluate different crRNA expression cassettes (related to Figure 3).

**Figure S7** *cas* systems used to evaluate different promoters and terminators (related to Figure 4).

**Figure S8** *cas* systems used to evaluate NLS variants (related to Figure 5).

**Figure S9** *cas* systems used to evaluate influence of intron presence in *cas12a* (related to Figure 6).

**Figure S10** *cas12a* and *cas9* system used for side-by-side comparison (related to Figure 8).

**Figure S11** Successful transient transformation of leaves and leaflets of three plant species.

**Figure S12** *cas* systems used in this study are active in plant cells (I).

**Figure S13** *cas* systems used in this study are active in plant cells (II).

**Figure S14** *cas* systems used in this study were not active in *Agrobacterium*.

**Table S1** Abbreviations used for tables and appendices in supplement.

**Table S2** Golden Gate LII and LIII plasmids.

**Table S3** Primers, gene strands and oligonucleotides used in this study.

**Table S4** Spacer sequences used in this study.

**Data S1** Sequences of plasmids. RAR archive of annotated maps of plasmids (Genbank format).

**Appendix S1** crRNA expression cassette *LjUbi_pro__RZ (cas12a).*

**Appendix S2** crRNA expression cassette *LjUbi_pro__ T_4_AT_6_ (cas12a).*

**Appendix S3** crRNA expression cassette *LjU6_pro__RZ (cas12a).*

**Appendix S4** crRNA expression cassette *2x LjU6_pro__2xRZ (cas12a).*

**Appendix S5** crRNA expression cassette *MtU6_pro__SP (cas12a)*.

**Appendix S6** crRNA expression cassette *AtU6MoClo_pro__SP (cas12a).*

**Appendix S7** crRNA expression cassette *AtU6-1_pro__SP (cas12a).*

**Appendix S8** crRNA expression cassette *AtU6-26_pro__SP (cas12a).*

**Appendix S9** sgRNA expression cassette *2x AtU6-26_pro_ (cas9).*

**Appendix S10** Oligonucleotide with Spacer 1 and Spacer 2 templates for cloning into the crRNA expression cassettes *LjU6_pro__SP (cas12a)*, *MtU6_pro__SP (cas12a)*, *AtU6MoClo_pro__SP (cas12a)*, *AtU6-1_pro__SP (cas12a)* and *AtU6-26_pro__SP (cas12a)*.

**Appendix S11** Oligonucleotide with Spacer 1 template for cloning into the crRNA expression cassettes *LjUbi_pro__RZ (cas12a)*, *LjU6_pro__RZ (cas12a)*, *2x LjU6_pro__2xRZ (cas12a)* and *2x AtU6-26_pro__2xRZ (cas12a).*

**Appendix S12** Oligonucleotide with Spacer 2 template for cloning into the crRNA expression cassettes *LjUbi_pro__RZ (cas12a)*, *LjU6_pro__RZ (cas12a)*, *2x LjU6_2xRZ (cas12a)* and *2x AtU6-26_pro__2xRZ (cas12a).*

**Appendix S13** Oligonucleotide with Spacer 1 and Spacer 2 templates for cloning into the crRNA expression cassette *LjUbi_pro__T_4_AT_6_ (cas12a).*

**Appendix S14** Oligonucleotide with Spacer 1 template for cloning into the sgRNA expression cassette *2x AtU6-26_pro_ (cas9).*

**Appendix S15** Oligonucleotide with Spacer 2 template for cloning into the sgRNA expression cassette *2x AtU6-26_pro_ (cas9).*

**Appendix S16** Coding sequence of *cas12a^Lj^*.

**Appendix S17** Open reading frame of *cas12a^At D156R^::introns*.

**Appendix S18** Reference of the intron sequences in *cas12a^At D156R^::introns.*

**Appendix S19** Sequence of the *Lotus japonicus* codon adapted SV40 NLS region.

**Appendix S20** Sequence of the *Lotus japonicus* codon adapted c-Myc NLS region.

**Appendix S21** Sequence of the *Lotus japonicus* codon adapted NLP NLS region.

**Appendix S22** Sequence of the *Lotus japonicus* codon adapted Tus NLS region.

**Appendix S23** Sequence of the *Lotus japonicus* codon adapted EGL-13 NLS region.

**Appendix S24** ORF of *csy4* with flanking 5’ and 3’ targets in *csy4* expression cassette version (I).

**Appendix S25 ORF of c*sy4* with flanking 5’ and 3’ targets in *csy4* expression cassette version (II).**

**
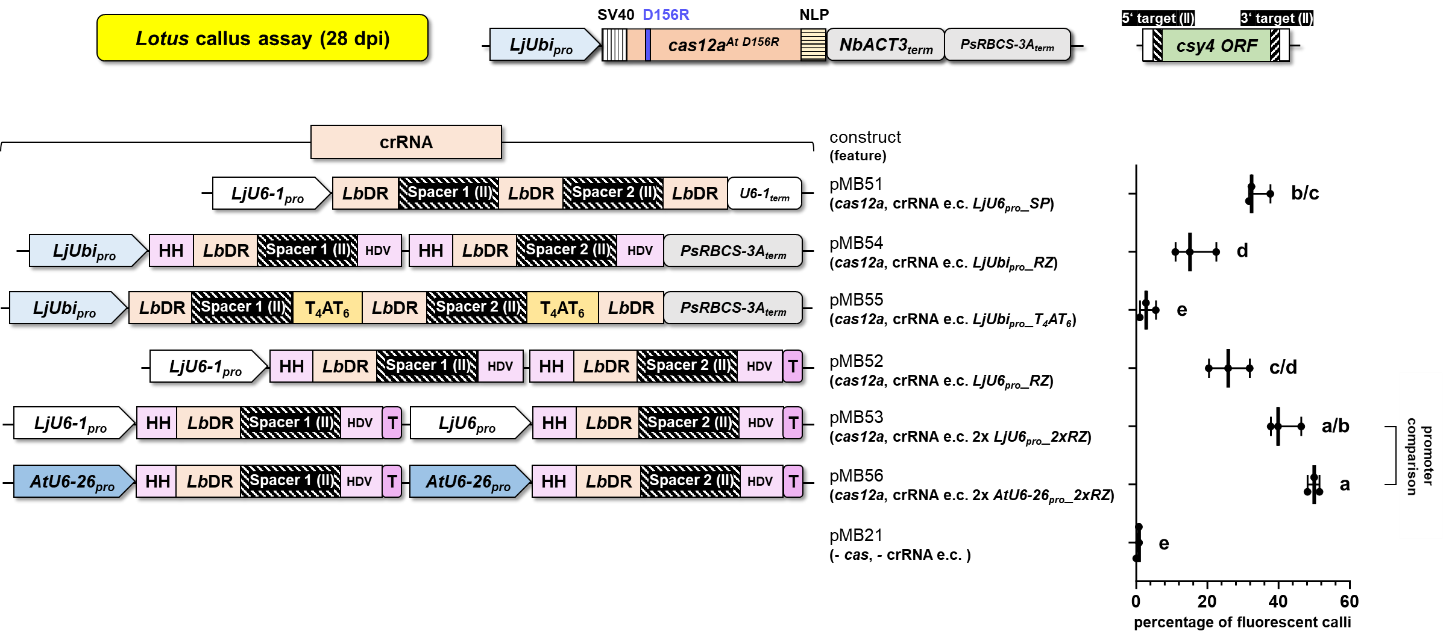
**

**Figure S1 Influence of different crRNA expression cassettes on the editing efficiency of a *cas12a* system.**

Influence of different crRNA expression cassettes (e.c.) in *Lotus callus* assay. Ratio of living fluorescent to non-fluorescent calli after transformation of *L. japonicus* hypocotyl cells with corresponding constructs carrying the indicated Csy4 target sequence version (II) (see Bircheneder et al., 2024). Note, the *cas* system with the crRNA expression cassette “*2x AtU6-26pro_2xRZ (cas12a)*” led to the highest editing efficiency of this specific *cas* system. The different crRNA expression cassettes are designed to produce two crRNAs with Spacer 1 (II) and Spacer 2 (II) sequences (see Table S3). Note that Spacer 1 (II) and Spacer 2 (II) are designed to address the target version (II), flanking the *csy4* ORF (Bircheneder et al., 2024). Cas endonuclease *cas12a^At D156R^* ORF is fused to the nuclear localization sequence (NLS) SV40 and NLP at its 5’ and 3’ end. A detailed description of the crRNA expression cassettes used can be found in Appendix S1 – S8, Table S3 and Bircheneder et al. (2024).

*35Spro*, Cauliflower mosaic virus 35S RNA gene promoter; *LjUbi_pro_*, *Lotus japonicus* polyubiquitin promoter; SV40, nuclear localization signal of the Simian Virus 40; NLP, nuclear localization signal from nucleoplasmin of *Xenopus laevis*; *cas12a^At D156R^*, *A. thaliana* (At) codon adapted *cas12a* gene encoding the D156R replacement; crRNA expression cassettes (e.c.) with Spacer sequences (II) (Appendix S1 – S4, Table S3 and Bircheneder et al., 2024); *Lb*DR, *Lachnospiraceae bacterium* (*Lb*) direct repeat; HH, hammerhead ribozyme; HDV, hepatitis delta virus ribozyme; *AtU6-26_pro_*, *Arabidopsis thaliana (At)* RNA polymerase III promoter U6-26; *LjU6-1_pro_*, *Lotus japonicus (Lj)* RNA polymerase III promoter U6-1; T, poly-T; T_4_AT_6_, nucleotide sequence TTTTATTTTTT; *Ps*, *Pisum sativum*; *Nb, Nicotiana benthamiana*. Expression of *cas12a^At D156R^* is controlled by the promoter *LjUbi_pro_* and the tandem terminators *NbACT3_term_* + *PsRBCS-3A_term_*. One way ANOVA followed by Tukey test was performed for the whole data set and values with no significant difference to each other were grouped by letters (a - e). Values labelled with different letters are statistically significantly different. Values labelled with two letters are statistically not different to values with corresponding single letters. One way ANOVA: P<0.0001.

**
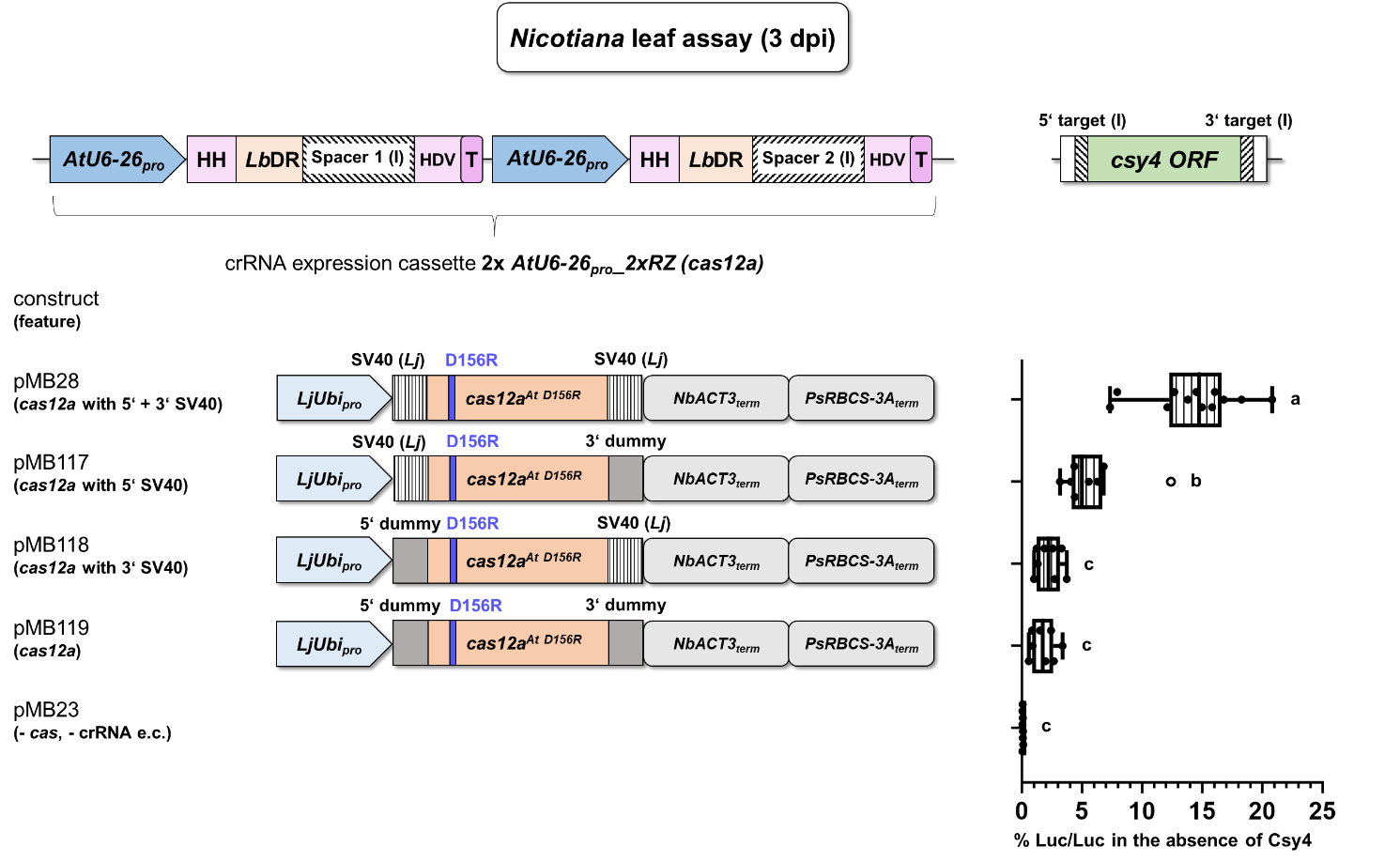
**

**Figure S2 Influence of NLS presence flanking *cas12a* on the editing efficiency of a *cas12a* system.**

Efficiency of nuclear localization signal (NLS) presence flanking Cas12a in Firefly luciferase assay. Quantification of Firefly luciferase activity in *N. benthamiana* leaf disc cells transformed with corresponding constructs including the *csy4* cassette with Cas targets version (I) (Bircheneder et al., 2024), normalized to *Renilla* luciferase activity. Note, Firefly luciferase activity could be restored best by the presence of both the 5’ and 3’ SV40, indicating a significantly higher editing efficiency of this specific *cas12a* system. The *cas12a* systems consist of a *cas12a* and a crRNA expression cassette. The crRNA expression cassette “2x *AtU6-26_pro__2xRZ (cas12a)*” is designed to produce two crRNAs with Spacer 1 (I) and Spacer 2 (I) sequences (see Table S3). Note that Spacer 1 (I) and Spacer 2 (I) are designed to address the target version (I), flanking the *csy4* ORF (Bircheneder et al., 2024). Cas endonuclease *cas12a^At D156R^* ORFs are fused to nuclear localization sequence (NLS) SV40 at their 5’ and 3’ end as indicated. A detailed description of the crRNA expression cassette “*2x AtU6-26pro_2xRZ (cas12a)*” can be found in Table S3 and Bircheneder et al. (2024).

*LjUbi_pro_*, *Lotus japonicus* polyubiquitin promoter; SV40 (*Lj*), nuclear localization signal of the Simian Virus 40 with codon usage of *Lotus japonicus*; *Pisum sativum*; *Nb, Nicotiana benthamiana;* *Lb*DR, *Lachnospiraceae bacterium* (*Lb*) direct repeat; HH, hammerhead ribozyme; HDV, hepatitis delta virus ribozyme; *AtU6-26_pro_*, *Arabidopsis thaliana (At)* RNA polymerase III promoter U6-26; T, poly-T; *cas12a^At D156R^*, *A. thaliana* (At) codon adapted *cas12a* gene encoding the D156R replacement; e.c., expression cassette; % Luc/Luc, Firefly luciferase activity normalized to *Renilla* luciferase activity. The control without Cas and without Csy4 (pMB24; - *cas*, - *csy4*) was used as 100% reference. Expression of *cas12a^At D156R^* is controlled by the promoter *LjUbi_pro_* and the tandem terminators *NbACT3_term_* + *PsRBCS-3A_term_*. One way ANOVA followed by Tukey test was performed for the whole data set and values with no significant difference to each other were grouped by letters (a - c). Values labelled with different letters are statistically significantly different. Values labelled with two letters are statistically not different to values with corresponding single letters. One way ANOVA: P<0.0001.


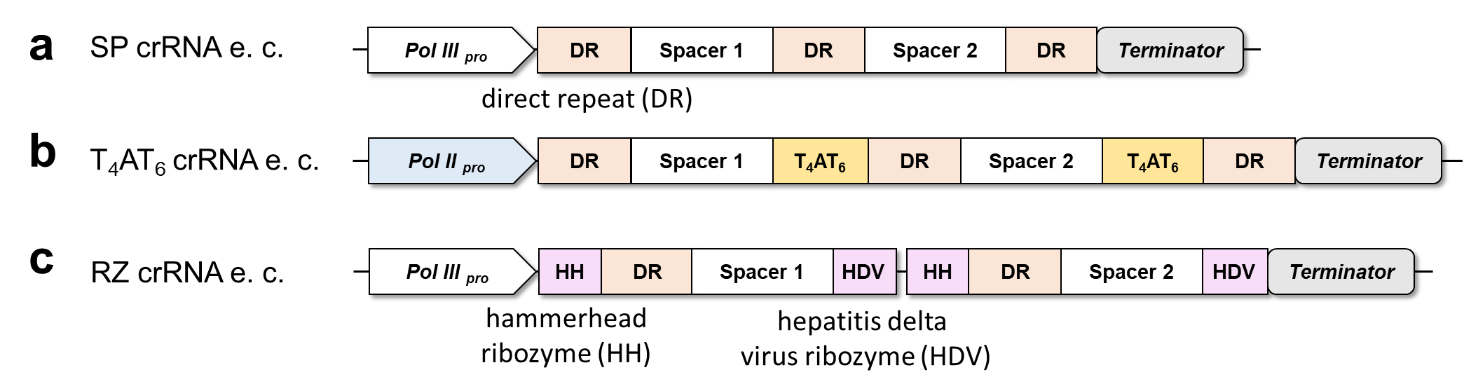


**Figure S3 Major systems for crRNA processing in a *cas12a* system in multiplex genome editing.**

(**a**) Self-processing (SP) crRNA expression cassette (e.c.) controlled by a polymerase III promoter. Spacer sequences are separated by direct repeats (DR) each and the expressed precursor CRISPR RNA (pre-crRNA) is processed by the Cas12a itself (Zetsche et al., 2017). (**b**) T_4_AT_6_ crRNA expression cassette driven by a polymerase II promoter. Here an artificial T_4_AT_6_ overhang is added to the 3'-end of the target sequence, which creates an U_4_AU_4_ overhang in the crRNA (Bin Moon et al., 2018). (**c**) Double ribozyme (RZ) crRNA expression cassette. The DR-spacer sequence is flanked by the hammerhead ribozyme (HH) and the hepatitis delta virus ribozyme (HDV) that can mediate precise intramolecular RNA cleavage and therefore precisely processes the pre-crRNAs (Ferré-D'Amaré and Scott, 2010; Gao and Zhao, 2014; Tang et al., 2017; Gao et al., 2018). The RZ crRNA expression cassette can be driven by polymerase II and polymerase III promoters (Tang et al., 2017; Zhang et al., 2021).


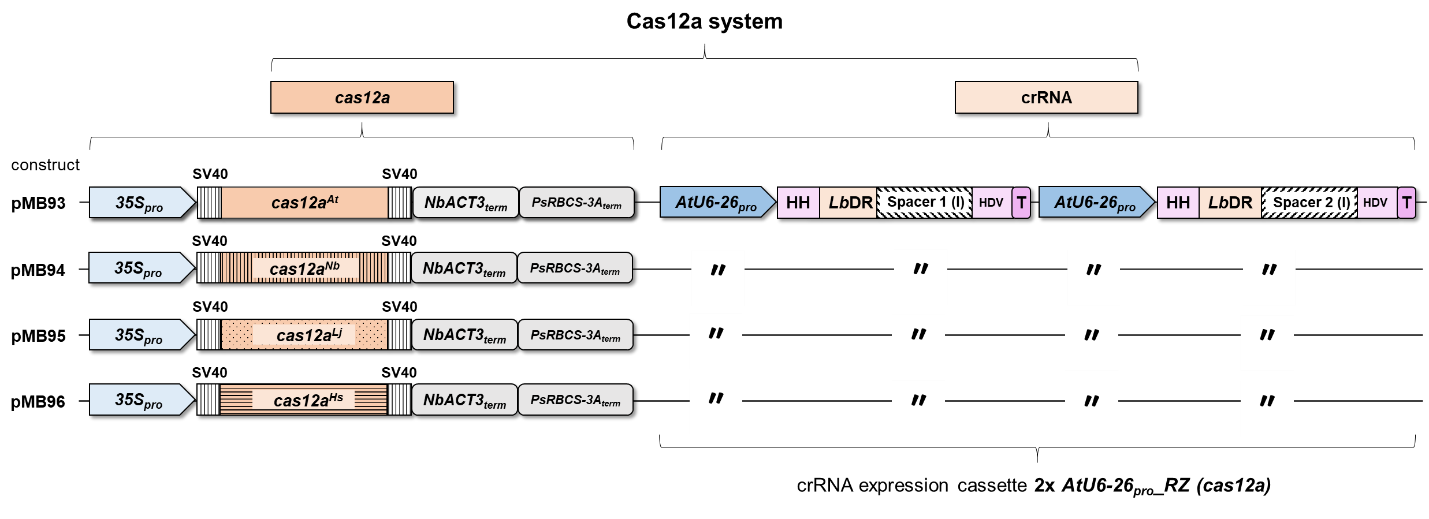


**Figure S4 *cas* systems used for the comparison of recoded *cas12a* versions (related to Figure 2).**

The *cas12a* systems consist of a *Cas12a* and a crRNA expression cassette. The crRNA expression cassette “2x *AtU6-26_pro__2xRZ (cas12a)*” is designed to produce two crRNAs with Spacer 1 (I) and Spacer 2 (I) sequences (see Table S3). Note that Spacer 1 (I) and Spacer 2 (I) are designed to address the target version (I), flanking the *csy4* ORF (Bircheneder et al., 2024). Cas endonuclease ORFs are fused to the nuclear localization sequences (NLS) SV40 at their 5’ and 3’ end as indicated. A detailed description of the crRNA expression cassette “*2x AtU6-26pro_2xRZ (cas12a)*” can be found in Table S3 and Bircheneder et al. (2024).

*35S_pro_*, Cauliflower mosaic virus 35S RNA gene promoter; SV40, NLS of the Simian Virus 40; *Lb*DR, *Lachnospiraceae bacterium* (*Lb*) direct repeat; HH, hammerhead ribozyme; HDV, hepatitis delta virus ribozyme; *AtU6-26_pro_*, *Arabidopsis thaliana (At)* RNA polymerase III promoter U6-26; T, poly-T; *Ps*, *Pisum sativum*; *cas12a^At^*, *Arabidopsis thaliana (At)* codon adapted *cas12a* gene; *cas12a^Nb^*, *Nicotiana benthamiana (Nb)* codon adapted *cas12a* gene*; cas12a^Lj^,* *Lotus japonicus (Lj)* codon adapted *cas12a* gene*; cas12a^Hs^*, *homo sapiens* (h) codon adapted *cas12a* gene; expression of the *cas12a* versions used is controlled by the promoter *35S_pro_* and the tandem terminators *NbACT3_term_* + *PsRBCS-3A_term_*.

**
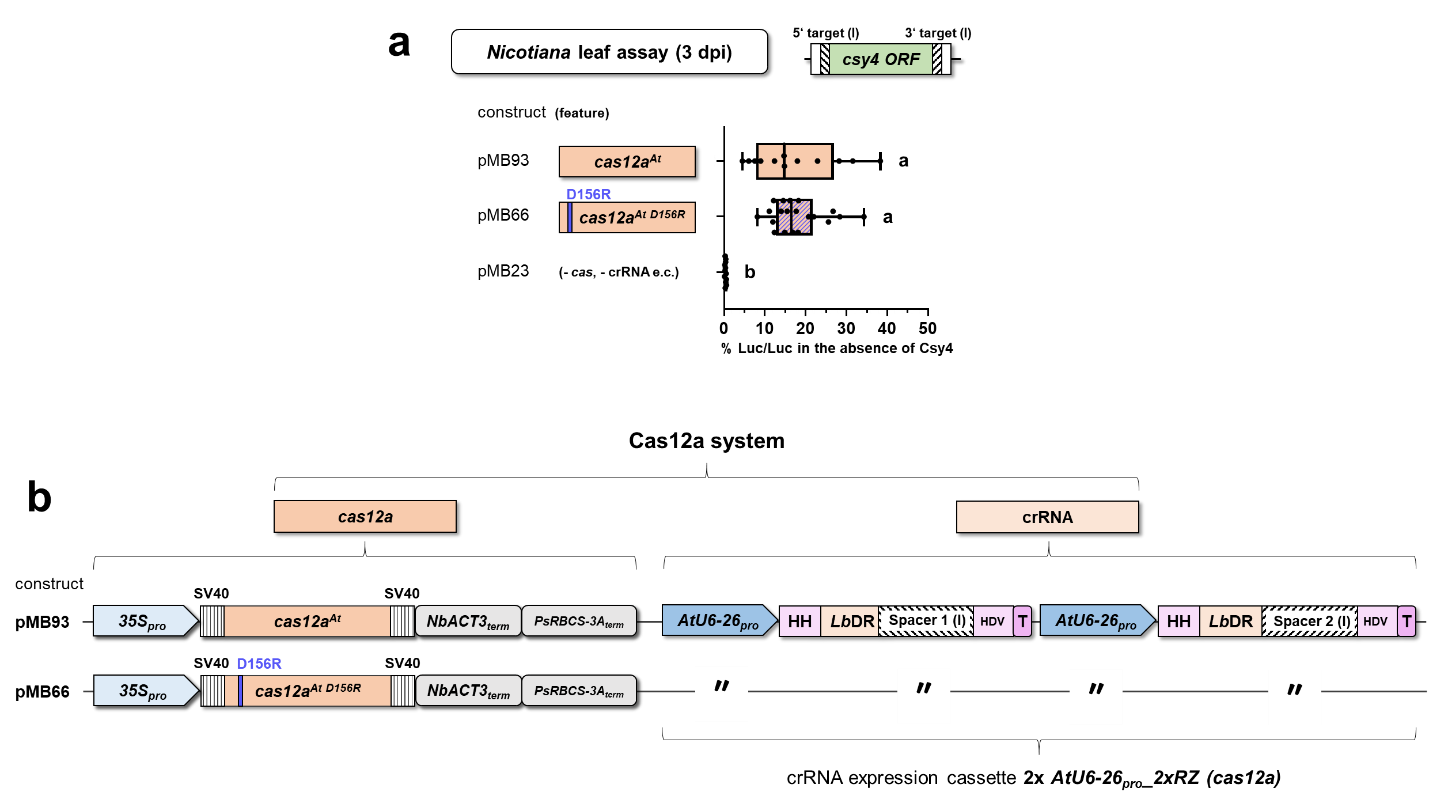
**

**Figure S5 *cas* systems used to evaluate the influence of the D156R mutation in *cas12a^At^*.**

(**a**) Efficiency of D156R presence in *cas12a* in Firefly luciferase assay. On T-DNAs of used constructs, *cas12a^At^* incorporated the D156R mutation (pMB66) or not (pMB93). D156R leads to a temperature tolerant version of Cas12a (Schindele and Puchta, 2020). Quantification of Firefly luciferase activity in *N. benthamiana* leaf disc cells transformed with corresponding constructs including the *csy4* cassette with Cas targets version (I) (Bircheneder et al., 2024), normalized to *Renilla* luciferase activity. Note, the *cas12a* system harboring the D156R mutation did not lead to a significantly higher Firefly luciferase activity than the compared *cas12a* system without D156R mutation within this system.

(**b**) A detailed description of the *cas12a* systems used in (**a**). The *cas12a* systems consist of a *cas12a* and a crRNA expression cassette. The crRNA expression cassette “2x *AtU6-26_pro__2xRZ (cas12a)*” is designed to produce two crRNAs with Spacer 1 (I) and Spacer 2 (I) sequences (see Table S3). Note that Spacer 1 (I) and Spacer 2 (I) are designed to address the target version (I), flanking the *csy4* ORF (Bircheneder et al., 2024). Cas endonuclease ORFs are fused to the nuclear localization sequence (NLS) SV40 at their 5’ and 3’ end as indicated. A detailed description of the crRNA expression cassette “*2x AtU6-26pro_2xRZ (cas12a)*” can be found in Table S3 and Bircheneder et al. (2024).ORF, open reading frame; *cas12a^At^*, expression cassette of *Arabidopsis thaliana* (*At*) codon adapted *cas12a* gene; *cas12a^At D156R^*, *A. thaliana* (At) codon adapted *cas12a* gene encoding the D156R replacement; e.c., expression cassette; % Luc/Luc, Firefly luciferase activity normalized to *Renilla* luciferase activity; *35S_pro_*, Cauliflower mosaic virus 35S RNA gene promoter; SV40, NLS of the Simian Virus 40; *Lb*DR, *Lachnospiraceae bacterium* (*Lb*) direct repeat; HH, hammerhead ribozyme; HDV, hepatitis delta virus ribozyme; *AtU6-26_pro_*, *Arabidopsis thaliana (At)* RNA polymerase III promoter U6-26; T, poly-T; *Ps*, *Pisum sativum*; *Nb, Nicotiana benthamiana*; *e*xpression of the *cas12a* versions used is controlled by the promoter *35S_pro_* and the tandem terminators *NbACT3_term_* + *PsRBCS-3A_term_*; one way ANOVA followed by Tukey test was performed for the whole data set and values with no significant difference to each other were grouped by letters (a - c). Values labelled with different letters are statistically significantly different. Values labelled with two letters are statistically not different to values with corresponding single letters. One way ANOVA: P<0.0001.

**
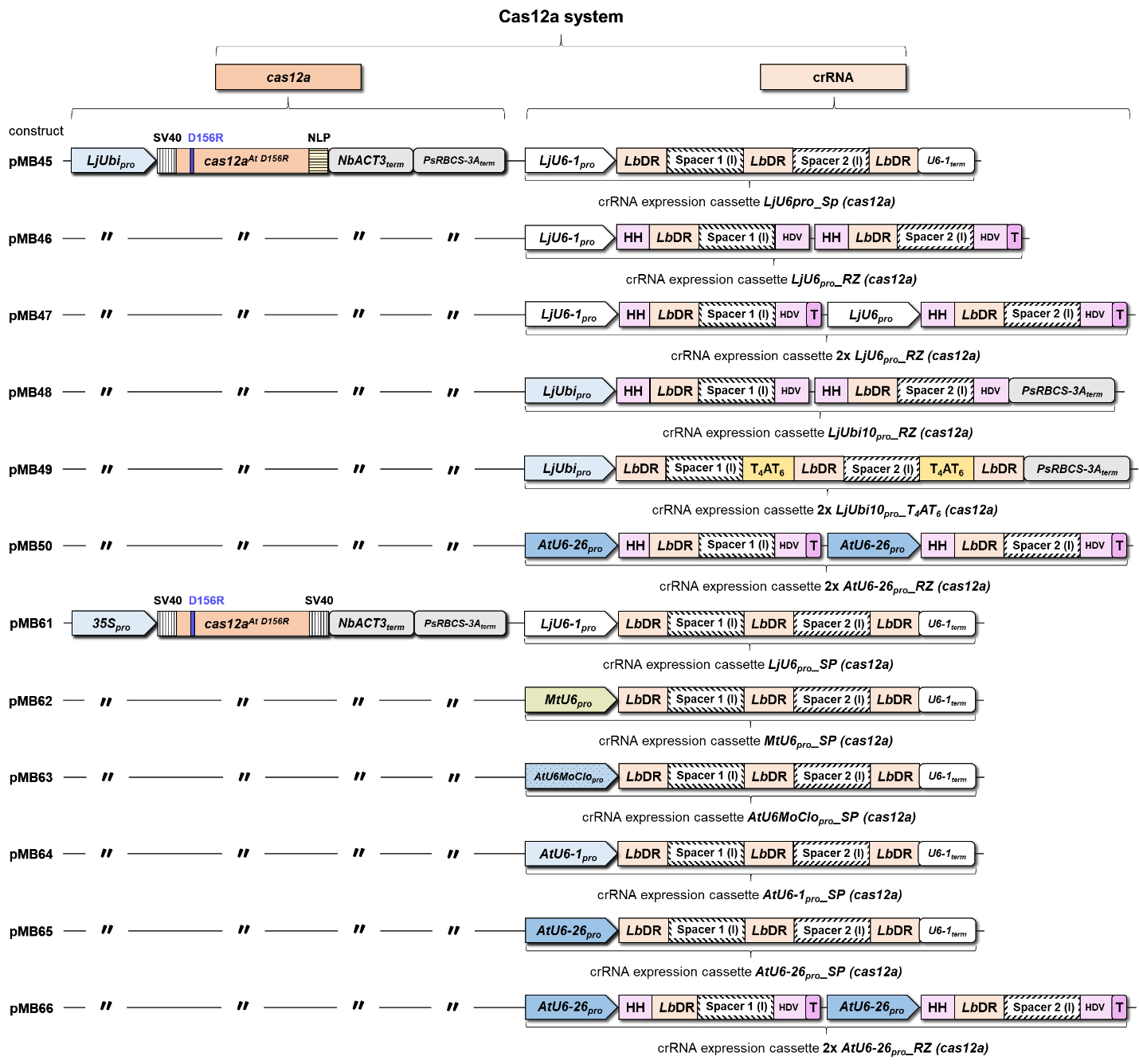
**

**Figure S6 *cas* systems used to evaluate different crRNA expression cassettes (related to Figure 3).**

The *cas12a* systems consist of a *cas12a* and a crRNA expression cassette. The different crRNA expression cassettes are designed to produce two crRNAs with Spacer 1 (I) and Spacer 2 (I) sequences (see Table S3). Note that Spacer 1 (I) and Spacer 2 (I) are designed to address the target version (I), flanking the *csy4* ORF (Bircheneder et al., 2024). Cas endonuclease *cas12a^At D156R^* ORFs are fused to the nuclear localization sequence (NLS) SV40 and NLP at its 5’ and 3’ end as indicated. A detailed description of the crRNA expression cassettes used can be found in Appendix S1 – S8, Table S3 and Bircheneder et al. (2024).

*35S_pro_*, Cauliflower mosaic virus 35S RNA gene promoter; *LjUbi_pro_*, *Lotus japonicus* polyubiquitin promoter; SV40, NLS of the Simian Virus 40; NLP, NLS from nucleoplasmin of *Xenopus laevis*; *cas12a^At D156R^*, *A. thaliana* (At) codon adapted *cas12a* gene encoding the D156R replacement; *Lb*DR, *Lachnospiraceae bacterium* (*Lb*) direct repeat; HH, hammerhead ribozyme; HDV, hepatitis delta virus ribozyme; *AtU6-16_pro_*, *Arabidopsis thaliana (At)* RNA polymerase III promoter U6-1; *AtU6-26_pro_*, *Arabidopsis thaliana (At)* RNA polymerase III promoter U6-26; *AtU6MoClo_pro_*, *Arabidopsis thaliana (At)* RNA polymerase III promoter U6MoClo; *MtU6_pro_*, *Medicago truncatula (Mt)* RNA polymerase III promoter U6; *LjU6-1_pro_*, *Lotus japonicus (Lj)* RNA polymerase III promoter U6-1; T, poly-T; T_4_AT_6_, nucleotide sequence TTTTATTTTTT; *Ps*, *Pisum sativum*; *Nb, Nicotiana benthamiana*; expression of *cas12a^At D156R^* is controlled by the promoters *35S_pro_* or *LjUbi_pro_* and the tandem terminators *NbACT3_term_* + *PsRBCS-3A_term_*.

**
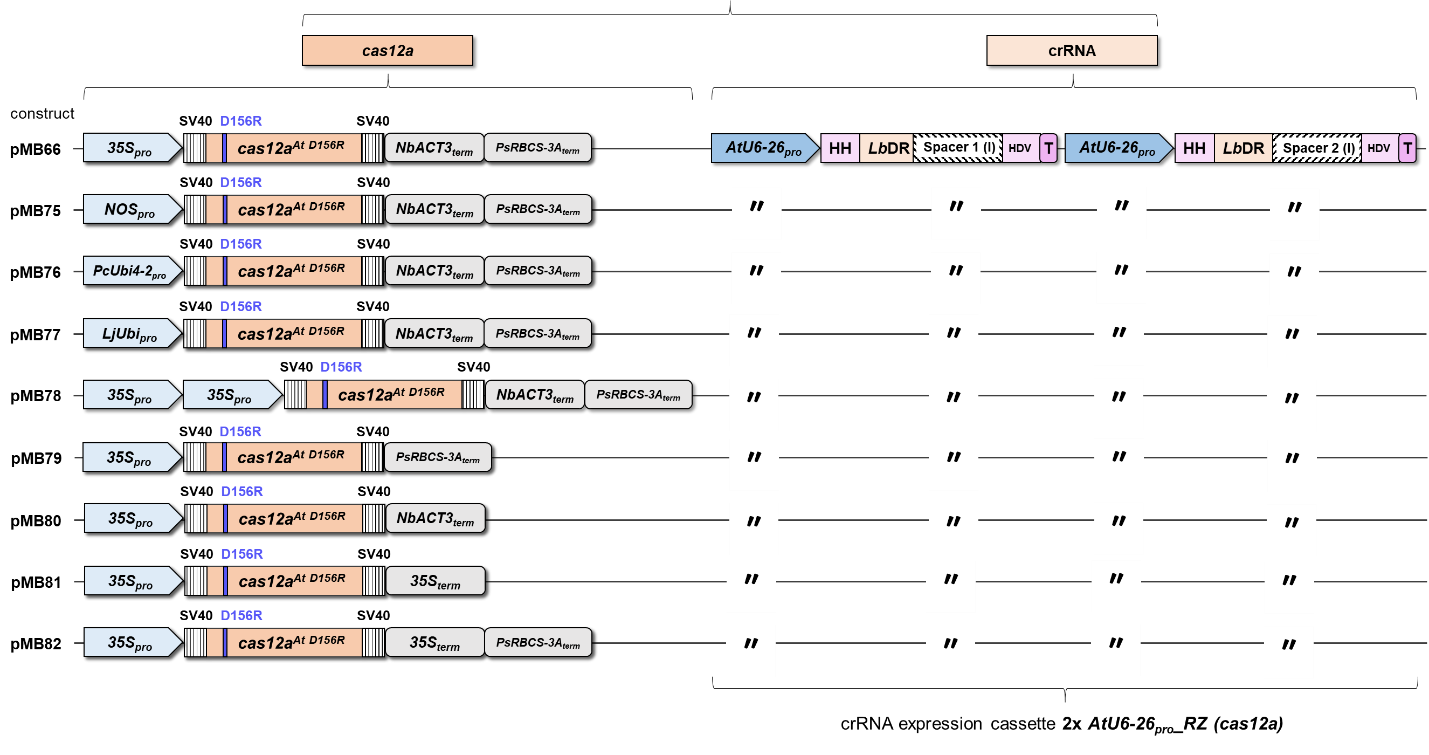
**

**Figure S7 *cas* systems used to evaluate different promoters and terminators (related to Figure 4).**

The *cas12a* systems consist of a *cas12a* and a crRNA expression cassette. The crRNA expression cassette “2x *AtU6-26_pro__2xRZ (cas12a)*” is designed to produce two crRNAs with Spacer 1 (I) and Spacer 2 (I) sequences (see Table S3). Note that Spacer 1 (I) and Spacer 2 (I) are designed to address the target version (I), flanking the *csy4* ORF (Bircheneder et al., 2024). Cas endonuclease *cas12a^At D156R^* ORF is fused to the nuclear localization sequence (NLS) SV40 at its 5’ and 3’ end. A detailed description of the crRNA expression cassette “*2x AtU6-26pro_2xRZ (cas12a)*” can be found in Table S3 and Bircheneder et al. (2024). Expression of *cas12a^At D156R^* is controlled by the promoters and the terminators as indicated here and in detail in Table S1.

*35S_pro_*, Cauliflower mosaic virus 35S RNA gene promoter; *LjUbi_pro_*, *Lotus japonicus* polyubiquitin promoter; *NOS_pro_,* Nopaline synthase promoter; *PcUbi4-2_pro_; Petroselinum crispum Ubi4-2* promoter*; NbACT3_term_, Nicotiana benthamiana (Nb) ACT3* terminator; *PsRBCS-3A_term_*, *Pisum sativum* *(Ps)* *RBCS-3A* terminator; SV40, NLS of the Simian Virus 40; *Lb*DR, *Lachnospiraceae bacterium* (*Lb*) direct repeat; HH, hammerhead ribozyme; HDV, hepatitis delta virus ribozyme; *AtU6-26_pro_*, *Arabidopsis thaliana (At)* RNA polymerase III promoter U6-26; T, poly-T; *cas12a^At D156R^*, *A. thaliana* (At) codon adapted *cas12a* gene encoding the D156R replacement;.

**
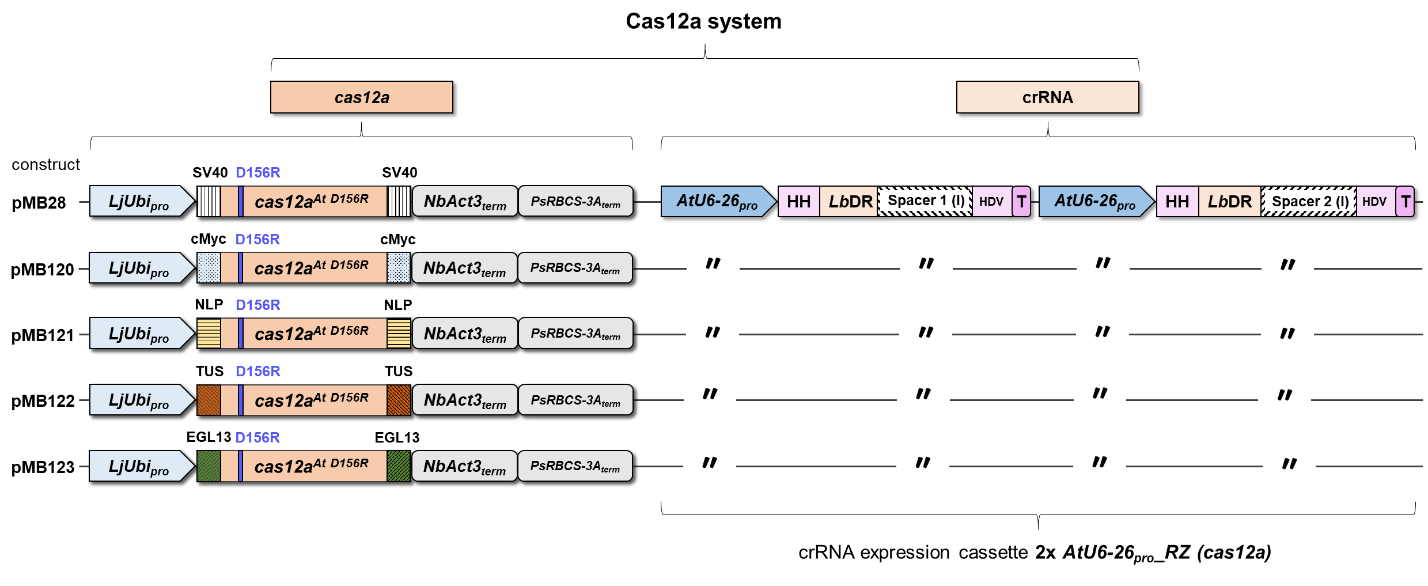
**

**Figure S8 *cas* systems used to evaluate NLS variants (related to Figure 5).**

The *cas12a* systems consist of a *cas12a* and a crRNA expression cassette. The crRNA expression cassette “2x *AtU6-26_pro__2xRZ (cas12a)*” is designed to produce two crRNAs with Spacer 1 (I) and Spacer 2 (I) sequences (see Table S3). Note that Spacer 1 (I) and Spacer 2 (I) are designed to address the target version (I), flanking the *csy4* ORF (Bircheneder et al., 2024). Cas endonuclease *cas12a^At D156R^* ORFs are fused to nuclear localization sequence (NLS) at their 5’ and 3’ end as indicated. A detailed description of the crRNA expression cassette “*2x AtU6-26pro_2xRZ (cas12a)*” can be found in Table S3 and Bircheneder et al. (2024).

*LjUbi_pro_*, *Lotus japonicus* polyubiquitin promoter; SV40 (*Lj*), NLS of the Simian Virus 40 with codon usage of *Lotus japonicus*; NLP (*Lj*), NLS from nucleoplasmin of *Xenopus laevis* with codon usage of *Lotus japonicus*; c-Myc (*Lj*), NLS from the human c-Myc protein with codon usage of *Lotus japonicus*; Tus (*Lj*), NLS from with codon usage of *Lotus japonicus*; EGL-13 (*Lj*), NLS from the *Caenorhabditis elegans* transcription factor EGL-13 with codon usage of *Lotus japonicus*; *Pisum sativum*; *Nb, Nicotiana benthamiana;* *Lb*DR, *Lachnospiraceae bacterium* (*Lb*) direct repeat; HH, hammerhead ribozyme; HDV, hepatitis delta virus ribozyme; *AtU6-26_pro_*, *Arabidopsis thaliana (At)* RNA polymerase III promoter U6-26; T, poly-T; *cas12a^At D156R^*, *A. thaliana* (At) codon adapted *cas12a* gene encoding the D156R replacement; expression of *cas12a^At D156R^* is controlled by the promoter *LjUbi_pro_* and the tandem terminators *NbACT3_term_* + *PsRBCS-3A_term_*.

**
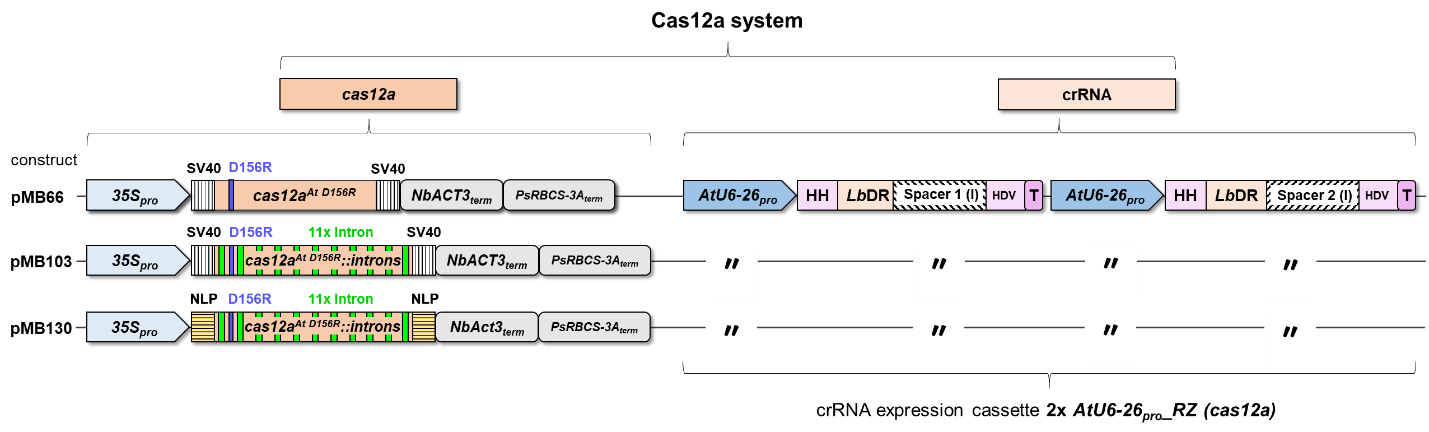
**

**Figure S9 *cas* systems used to evaluate influence of intron presence in *cas12a* (related to Figure 6).**

The *cas12a* systems consist of a *cas12a* and a crRNA expression cassette. The crRNA expression cassette “2x *AtU6-26_pro__2xRZ (cas12a)*” is designed to produce two crRNAs with Spacer 1 (I) and Spacer 2 (I) sequences (see Table S3). Note that Spacer 1 (I) and Spacer 2 (I) are designed to address the target version (I), flanking the *csy4* ORF (Bircheneder et al., 2024). Cas endonuclease ORFs are fused to the nuclear localization sequence (NLS) SV40 and NLP at their 5’ and 3’ end as indicated. A detailed description of the crRNA expression cassette “*2x AtU6-26pro_2xRZ (cas12a)*” can be found in Table S3 and Bircheneder et al. (2024).

*35S_pro_*, Cauliflower mosaic virus 35S RNA gene promoter; SV40, NLS of the Simian Virus 40; NLP, NLS from nucleoplasmin of *Xenopus laevis*; *Lb*DR, *Lachnospiraceae bacterium* (*Lb*) direct repeat; HH, hammerhead ribozyme; HDV, hepatitis delta virus ribozyme; *AtU6-26_pro_*, *Arabidopsis thaliana (At)* RNA polymerase III promoter U6-26; T, poly-T; *Ps*, *Pisum sativum*; *Nb, Nicotiana benthamiana; cas12a^At D156R^*, *A. thaliana* (At) codon adapted *cas12a* gene encoding the D156R replacement; *cas12a^At D156R^::introns*, *cas12a^At D156R^* gene carrying 11 introns (*i*), the added introns are represented by green bars; *e*xpression of the *cas12a* versions used is controlled by the promoter *35S_pro_* and the tandem terminators *NbACT3_term_* + *PsRBCS-3A_term_.*

**
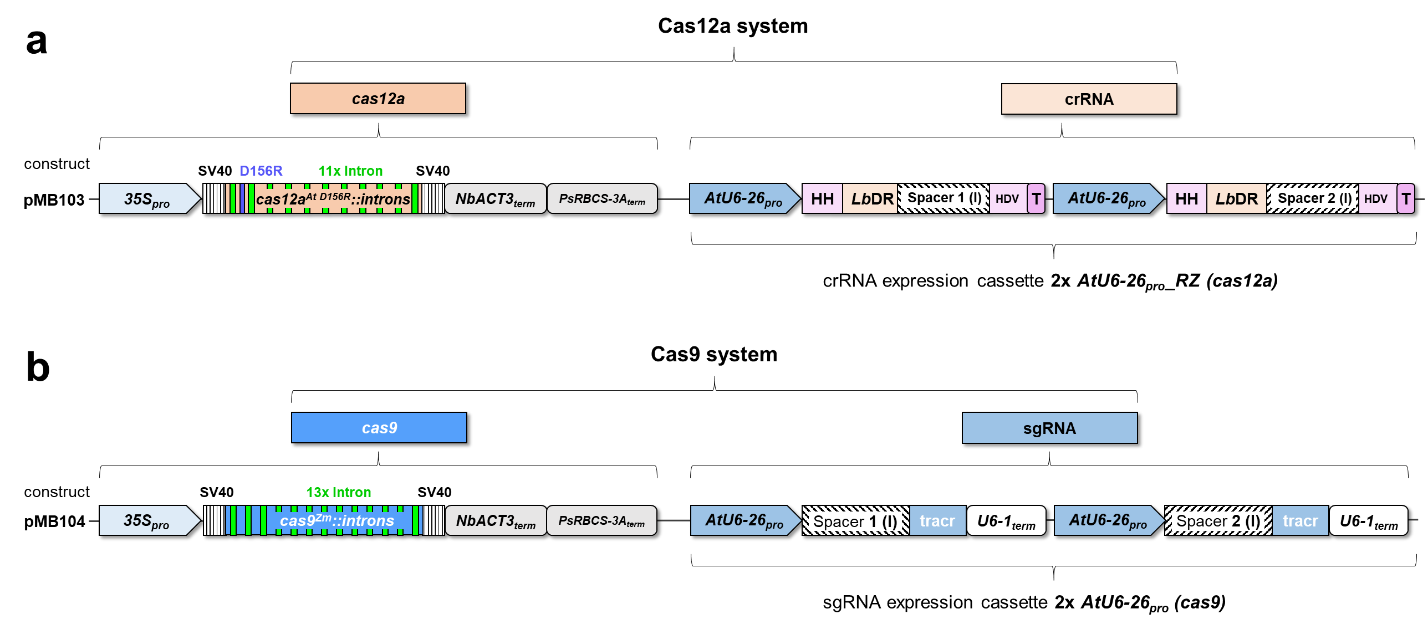
**

**Figure S10 *cas12a* and *cas9* system used for side-by-side comparison (related to Figure 8).**

**(a)** The *cas12a* system consist of a *cas12a* (*cas12a^At D156R^::introns*) and a crRNA expression cassette. The crRNA expression cassette,

“2x *AtU6-26_pro__2xRZ (cas12a)*” is designed to produce two crRNAs with Spacer 1 (I) and Spacer 2 (I) sequences (see Table S3). **(b)** The *cas9* system consist of a *cas9* (*cas9^Zm^::introns*) (Grützner et al., 2021) and a sgRNA expression cassette. The sgRNA expression cassette “2x *AtU6-26_pro_ (cas9)*” is designed to produce two sgRNAs one with Spacer 1 (I), one with Spacer 2 (I) sequences (Table S3). **(a** and **b)** Note that Spacer 1 (I) and Spacer 2 (I) are designed to address the target versions (I), flanking the *csy4* ORF (Bircheneder et al., 2024). Cas endonuclease ORFs are fused to the nuclear localization sequence (NLS) SV40 at their 5’ and 3’ end as indicated. A detailed description of the crRNA expression cassette “*2x AtU6-26pro_2xRZ (cas12a)*” can be found in Table S3 and Bircheneder et al. (2024). A detailed description of the sgRNA expression cassette “*2x AtU6-26_pro_ (cas9)*” can be found in Table S3 and Appendix S9.

*35S_pro_*, Cauliflower mosaic virus 35S RNA gene promoter; SV40, NLS of the Simian Virus 40; *Lb*DR, *Lachnospiraceae bacterium* (*Lb*) direct repeat; HH, hammerhead ribozyme; HDV, hepatitis delta virus ribozyme; *AtU6-26_pro_*, *Arabidopsis thaliana (At)* RNA polymerase III promoter U6-26; T, poly-T; *Ps*, *Pisum sativum*; *Nb*, *Nicotiana benthamiana*; *cas12a^At D156R^::introns*, *A. thaliana* (At) codon adapted *cas12a* gene encoding the D156R replacement and 11 introns (i); *cas9^Zm^::introns, Zea mays* codon adapted *cas9* gene from *Streptococcus pyogenes* (*Sp*) with added 13 introns (i); the added introns are represented by green bars, *e*xpression of the *Cas* versions used is controlled by the promoter *35S_pro_* and the tandem terminators *NbACT3_term_* + *PsRBCS-3A_term_*.

**
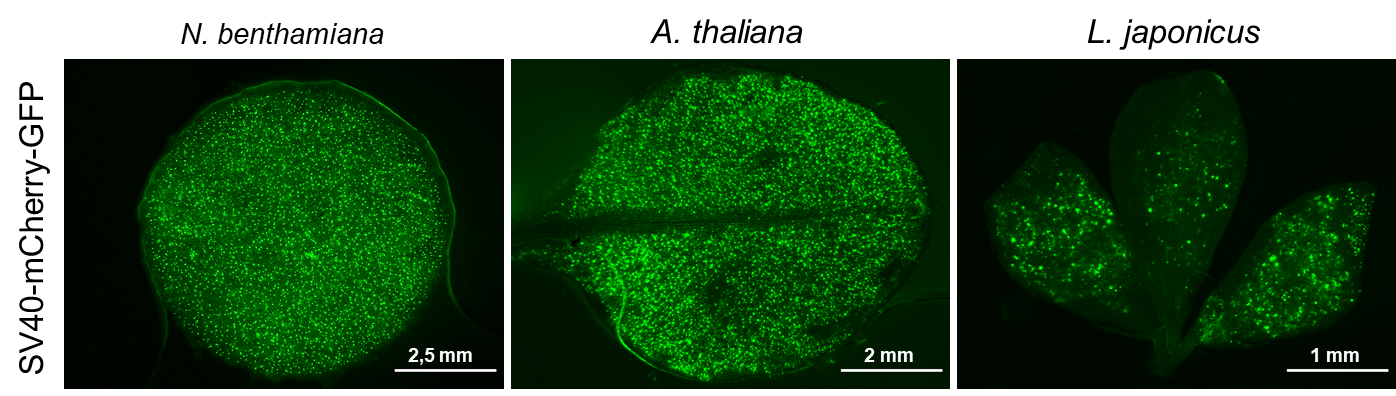
**

****Figure S11 Successful transient transformation of leaves and leaflets of three different plant species.****

Exemplary fluorescence microscope images of a *N. benthamiana* leave disc and a leaves of A*. thaliana* and the three leaflets of a *L. japonicus* leaf*,* 78 hours after infiltration with *A. tumefaciens* carrying plasmid F14. Note the transformation was performed as previously described **(Zhang et al., 2020).**

**
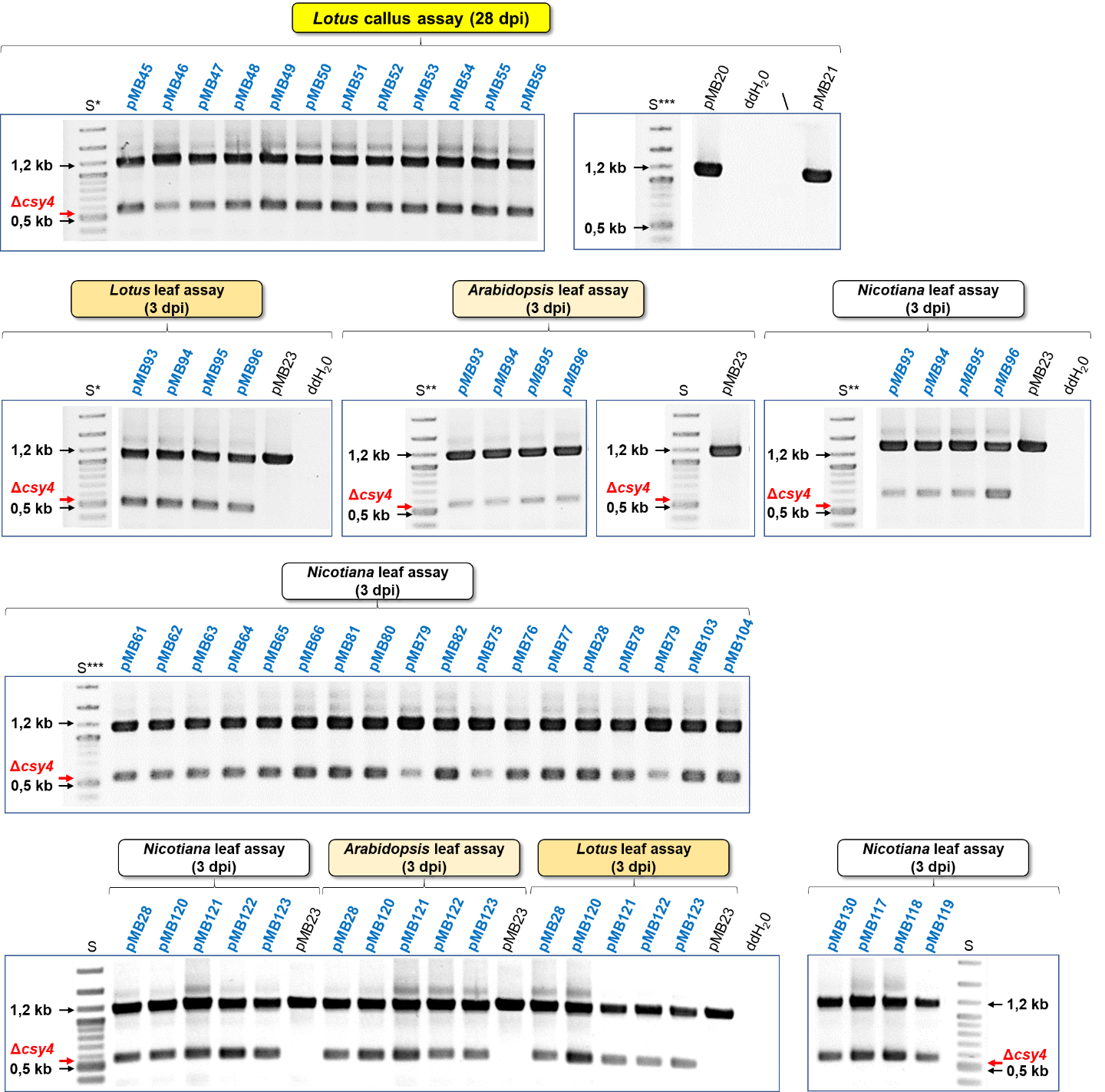
**

****Figure S12 *cas* systems used in this study are active in plant cells (I).****

Control experiment to test the capability of *cas* systems (Figure S1, S2, S4, S5b, S6, S7, S8, S9, and S10) to generate full deletions of the *csy4* ORF. Gel with PCR products from exemplary DNA templates isolated from plant tissue after transformation with T-DNA. The used primer pair pro_FW /term_Rev flanked the ORF of *csy4* to confirm Cas activity by band in the gel with the reduced size of approx. 530 bp, corresponding to a full deletion of approx. 630 bp (Δ*csy4*) (Bircheneder et al., 2024). Constructs harboring a *cas* system and *csy4* are highlighted in blue. Constructs harboring *csy4* but lacking a *cas* system (pMB20, pMB21 and pMB23) served as controls. Note that all constructs with a *cas* system (highlighted in blue) led to a Δ*csy4* sized gel band, indicating the functionality of their *cas* system. No conclusion could be drawn from the intensity of the full deletion bands about the editing efficiency of the corresponding constructs, as the amount of transformed T-DNA within the tissue material sampled for PCR analysis was not normalized. Note that each gel picture shown within a blue-framed box originates from the same gel, including reference standard. Gel lanes are combined here to document the presence or absence of the band representing Δ*csy4*. Intermediate space between the individual lanes was deleted for greater clarity. Note that reference standard lanes marked with asterisk (S*, S** and S***) are identical since the same lane was used twice to ease comparison with plasmid lanes originating from the same gel. All plasmids except of pMB20, pMB21, pMB23 and pMB104 contain *cas12a* systems (for details see Figure S1, S2, S4, S5b, S6, S7, S8, S9, and S11). The plasmid pMB104 contains a *cas9* system (for details see Figure S10b). pMB23 and pMB20 served as control with *csy4* (5’ + 3’ (I) targets), but without *cas* system (for details see Bircheneder et al. (2024)). pMB21 served as control with *csy4* (5’ + 3’ (II) targets) (for details see Bircheneder et al. (2024)).

dpi, days post inoculation; bp, base pair; kb, kilo base pair; S, 1 kb Plus ladder.


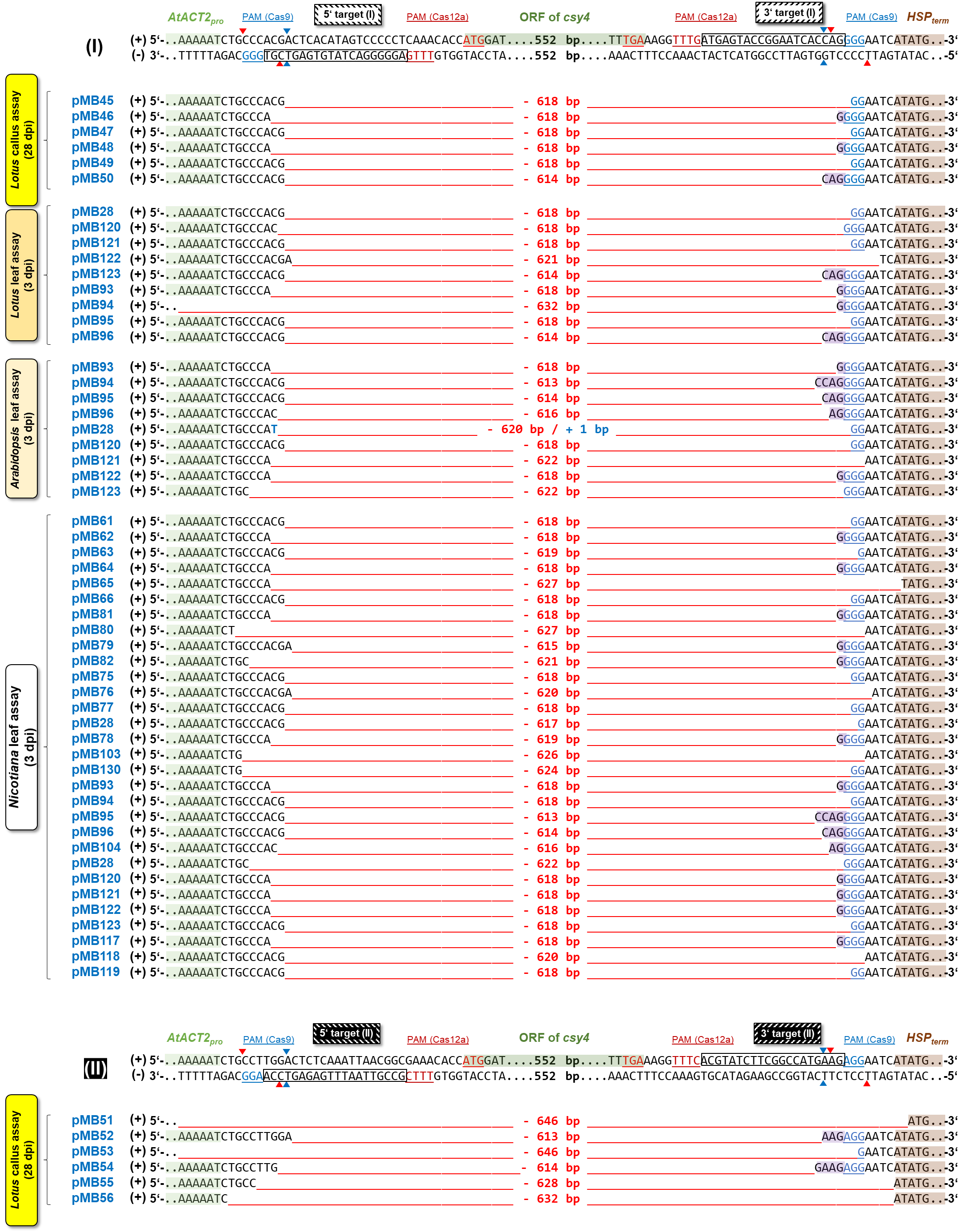


****Figure S13 *cas* systems used in this study are active in plant cells (II).****

Control experiment to test the capability of *cas* systems (Figure S1, S2, S4, S5b, S6, S7, S8, S9 and S10) to generate full deletions of the *csy4* ORF. Δ*csy4* fragments shown in Figure S12 were cloned into BB3, multiplied in *E. coli* and subjected to Sanger sequencing. Sequences of individual clones confirmed that the Δ*csy4* band derived from the loss of the ORF of *csy4*. Negative numbers in red indicate the total deletion size in bp. Note that although the plasmid of origin is indicated on the left, the deletions were randomly selected for display and there was no correlation analysis performed between plasmid and deletion size. Positive number and letter in blue indicate the insertion of a “T”.

(I), Sequence of the 5’ and 3’ target region of *csy4* expression cassette (I) as reference, sequencing results of Δ*csy4* band derived from corresponding constructs with *csy4* expression cassette (I) are listed underneath; (II), Sequence of the 5’ and 3’ target region of *csy4* expression cassette (II) as reference, sequencing results of Δ*csy4* band derived from corresponding constructs with *csy4* expression cassette (II) are listed underneath; (+), plus strand; (-), minus strand; 5’ target sequences (protospacers) and 3’ target sequences (protospacers) are framed; PAM, protospacer adjacent motif; PAM sequences are highlighted in dark red (PAM for *cas12a*) and blue (PAM for *cas9*) and are underlined; red triangles denote the potential cleavage sites of Cas12a; blue triangles denote the cleavage sites of Cas9; *AtACT2_pro_*, Actin2 promoter from *A. thaliana*; *HSP_term_*, heat shock protein terminator from *A. thaliana***.**


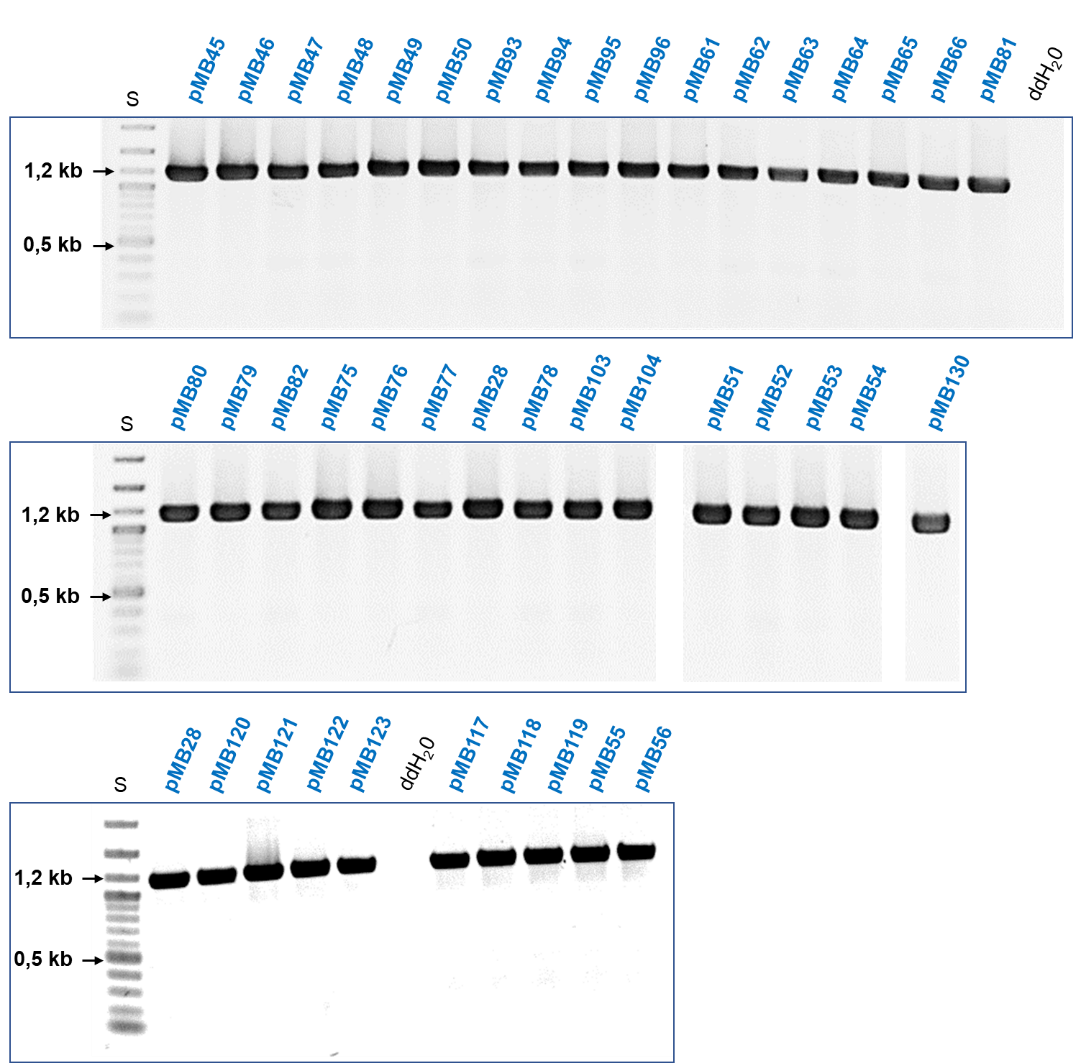


**Figure S14 *cas* systems used in this study were not active in *Agrobacterium***

Control experiment to test the capability of *cas* systems (Figure S1, S2, S4, S5b, S6, S7, S8, S9 and S10) to generate full deletions of the *csy4* ORF. Confirmation that the *csy4* deletions did not already occur in *E. coli* or *Agrobacterium* prior to inoculation of plants. Gel with PCR products from corresponding plasmid templates isolated from *Agrobacterium* strains harboring the indicated plasmids. PCR was performed with primer pair pro_FW /term_Rev flanking the ORF of *csy4* (Figure 5, Bircheneder et al., 2024). Note that no Δ*csy4* band was obtained indicated that deletion events did not occur during cultivation of plasmid-harboring bacteria. Note that each gel picture shown within a blue-framed box originates from the same gel, including reference standard. kb, kilo base pair ; S, 1 kb Plus ladder.

**Table S1 Abbreviations used for tables and appendices in supplement**.

| **Abbreviation** | **Description** |
| --- | --- |
| *35S_pro_* | Cauliflower mosaic virus 35S RNA gene promoter |
| *ATG:HP* | sequence of the 72 bp insertion between ATG and the remaining ORF of the *ATG:6xHis:HP:Firefly luciferase* reporter gene and sequence of the 75bp insertion between ATG and the remaining ORF of the *ATG:6xHis:HP:mCitrine-NLS* reporter gene |
| *AtU6-16_pro_* | *Arabidopsis thaliana (At)* RNA polymerase III promoter U6-1 |
| *AtU6-26_pro_* | *Arabidopsis thaliana (At)* RNA polymerase III promoter U6-26 |
| *AtU6MoClo_pro_* | *Arabidopsis thaliana (At)* RNA polymerase III promoter U6MoClo |
| *cas12a^At^* | *Arabidopsis thaliana (At)* codon adapted *cas12a* gene |
| *cas12a^At D156R^* | *A. thaliana* (At) codon adapted *cas12a* gene encoding the D156R replacement; |
| *cas12a^At D156R^::introns* | expression cassette with *A. thaliana* (At) codon adapted *cas12a* gene encoding the D156R replacement and 11 introns (*i*) |
| *cas12a^Lj^* | *Lotus japonicus (Lj)* codon adapted *cas12a* gene |
| *cas12a^Hs^* | *homo sapiens* (hs) codon adapted *cas12a* gene |
| *cas12a^Nb^* | *Nicotiana benthamiana (Nb)* codon adapted *cas12a* gene |
| *csy4* | endoribonuclease Csy4 gene |
| c-Myc | NLS from the human c-Myc protein |
| DMY | DMY, dummy sequences |
| EGL-13 | NLS from the *Caenorhabditis elegans* transcription factor EGL-13 |
| *GFP* | green fluorescent protein gene |
| HDV | hepatitis delta virus ribozyme |
| HH | hammerhead ribozyme |
| *Lb*DR | *Lachnospiraceae bacterium* direct repeat |
| *LjU6-1_pro_* | *Lotus japonicus (Lj)* RNA polymerase III promoter U6-1 |
| *LjUbi_pro_* | *Lotus japonicus* polyubiquitin promoter |
| *MtU6_pro_* | *Medicago truncatula (Mt)* RNA polymerase III promoter U6 |
| *Nb* | *Nicotiana benthamiana*. |
| *NbACT3_term_* | *Nicotiana benthamiana (Nb) ACT3* terminator |
| NLP | NLS from nucleoplasmin of *Xenopus laevis* |
| *NOS_pro_* | Nopaline synthase promoter |
| *PcUbi4-2_pro_* | *Petroselinum crispum Ubi4-2* promoter |
| *Ps* | *Pisum sativum* |
| *PsRBCS-3A_term_* | *Pisum sativum* *(Ps)* *RBCS-3A* terminator |
| Spc | spacer |
| *cas9^Zm^::introns* | *Zea mays* codon adapted *cas9* gene from *Streptococcus pyogenes* with added 13 introns (i) |
| SV40 | nuclear localization sequence (NLS) of the Simian Virus 40 |
| Tus | NLS from the *E. coli* replication fork arresting protein Tus |

**Table S2 Golden Gate level II (LII) and level III (LIII) plasmids.**

For creating level I (LI) plasmids containing inserts (with overhangs A-B, B-C, C-D, D-E, E-F, F-G or custom positions), the LI backbone BB3 was used. A to G are LI fusion sites of the Golden Gate system (GG) (Binder et al., 2014). Any other fusion site beside A to G was added within this work. Those sequences are elementary parts of the crRNA expression cassettes. Thereby, the crRNA expression cassettes are not interrupted by artificial fusion sites of the GG system. See table S1 for abbreviations.

| **Plasmid** | **AB** | **BC** | **CD** | **DE** | **EF** | **FG** | **Backbone** | **Level** | **Res.** | **Reference** |
| --- | --- | --- | --- | --- | --- | --- | --- | --- | --- | --- |
| **pMB1** | *NOS_pro_* | DMY | *hptII* (hygromycine resistance) gene | DMY | *35S_term_* | DMY | LIIβ F 1-2 - BB20 | II | Spec | - |
| **pMB13** | *LjUbi_pro_* | *ATG:HP* | *mCitrine* without start codon | SV40 | *NOS_term_* | DMY | LIIβ F 4-5 – BB26 | II | Spec | - |
| **pMB14** | *LjUbi_pro_* | *ATG:HP* | *Firefly luciferase* without start codon | DMY | *NOS_term_* | DMY | LIIβ F 4-5 – BB26 | II | Spec | - |
| **pMB15** | *ACT2_pro_* | 5‘ target (I) | *csy4* | 3‘ target (I) | *HSP_term_* | DMY | LIIβ F 5-6 – BB28 | II | Spec | - |
| **pMB18** | *ACT2_pro_* | 5‘ target (II) | *csy4* | 3‘ target (II) | *HSP_term_* | DMY | LIIβ F 5-6 – BB28 | II | Spec | - |
| **pMB19** | *NOS_pro_* | DMY | *Renilla luciferase* | DMY | *35S_term_* | DMY | LIIβ F 1-2 - BB20 | II | Spec | - |
|  | **1-2** | **2-3** | **3-4** | **4-5** | **5-6** | **-** | **Backbone** | **Level** | **Res.** | **Reference** |
| **pMB20** | pMB1 | DMY | DMY | pMB13 | pMB15 | - | LIIIβ F A-B - BB53 | III | Kan | Figure 3 and S12 |
| **pMB21** | pMB1 | DMY | DMY | pMB13 | pMB18 | - | LIIIβ F A-B - BB53 | III | Kan | Figure S1 and S12 |
| **pMB23** | pMB19 | DMY | DMY | pMB14 | pMB15 | - | LIIIβ F A-B - BB53 | III | Kan | Figure 2, 3, 4, 5, 6, 8, S2, S5 and S12 |
| **pMB24** | pMB19 | DMY | DMY | pMB14 | DMY | - | LIIIβ F A-B - BB53 | III | Kan | Figure 2, 3, 4, 5, 6, 8, S2 and S5 |
|  | **AB** | **BC** | **CD** | **DE** | **EF** | **FG** | **Backbone** | **Level** | **Res.** | **Reference** |
| **pMB25** | *LjUbi_pro_* | SV40 (*Lj*) | *cas12a^At D156R^* | SV40 (*Lj*) | *NbACT3_term_* | *PsRBCS-3A_term_* | LIIβ F 2-3 - BB22 | II | Spec | - |
|  | **1-2** | **2-3** | **3-4** | **4-5** | **5-6** | **-** | **Backbone** | **Level** | **Res.** | **Reference** |
| **pMB28** | pMB19 | pMB25 | pMB40 | pMB14 | pMB15 | **-** | LIIIβ F A-B - BB53 | III | Kan | Figure 5, S2, S8, S12, S13 and S14 |

| **Plasmid** | **AA^+^** | **A^+^A^-^** | | **A^-^ F** | | | **FG** | **Backbone** | **Level** | **Res.** | **Reference** |
| --- | --- | --- | --- | --- | --- | --- | --- | --- | --- | --- | --- |
| **pMB29** | *LjU6-1_pro_-Lb*DR | Oligonucleotide Spacer 1 + 2 (I)  for *LjU6_pro__SP (cas12a)* | | *U6-1_tem_* | | | DMY | LIIβ F 3-4 – BB24 | II | Spec | - |
| **pMB30** | *LjU6-1_pro_-Lb*DR | Oligonucleotide Spacer 1 + 2 (II)  for *LjU6_pro__SP (cas12a)* | | *U6-1_term_* | | | DMY | LIIβ F 3-4 – BB24 | II | Spec | - |
|  | **AB^+^** | **B^+^A^+^** | **A^+^C^+^** | **C^+^C^-^** | | **C^-^D^+^** | **D^+^G** | **Backbone** | **Level** | **Res.** | **Reference** |
| **pMB32** | *LjU6-1_pro_-*HH | HH-*Lb*DR | Oligonucleotide Spacer 1 (I) for *LjU6_pro_*_RZ (*cas12a*) | HDV-HH-*Lb*DR | | Oligonucleotide Spacer 2 (I) for *LjU6_pro_*_RZ (*cas12a*) | HDV-Poly-T | LIIβ F 3-4 – BB24 | II | Spec | - |
| **pMB33** | *LjU6-1_pro_-*HH | HH-*Lb*DR | Oligonucleotide Spacer 1 (II) for *LjU6_pro_*_RZ *(cas12a)* | HDV-HH-*Lb*DR | | Oligonucleotide Spacer 1 (II) for *LjU6_pro_*_RZ (*cas12a*) | HDV-Poly-T | LIIβ F 3-4 – BB24 | II | Spec | - |
|  | **AB^+^** | **B^+^A^+^** | **A^+^C^+^** | **C^+^D^-^** | **D^-^C^-^** | **C^-^D^+^** | **D^+^G** | **Backbone** | **Level** | **Res.** | **Reference** |
| **pMB34** | *LjU6-1_pro_-*HH | HH-*Lb*DR | Oligonucleotide Spacer 1 (I) for 2x *LjU6_pro_*_2xRZ (*cas12a*) | HDV-Poly-T*-LjU6-1_pro_-*HH | HH*-Lb*DR | Oligonucleotide Spacer 2 (I) for 2x *LjU6_pro_*_2xRZ (*cas12a*) | HDV-Poly-T | LIIβ F 3-4 – BB24 | II | Spec | - |
| **pMB35** | *LjU6-1_pro_-*HH | HH-*Lb*DR | Oligonucleotide Spacer 1 (II) for 2x *LjU6_pro_*_2xRZ (*cas12a*) | HDV-Poly-T*-LjU6-1_pro_-*HH | HH*-Lb*DR | Oligonucleotide Spacer 1 (II) for 2x *LjU6_pro_*_2xRZ (*cas12a*) | HDV-Poly-T | LIIβ F 3-4 – BB24 | II | Spec | - |
|  | **AB^+^** | **B^+^A^+^** | **A^+^C^+^** | **C^+^C^-^** | **C^-^D^+^** | **D^+^F** | **FG** | **Backbone** | **Level** | **Res.** | **Reference** |
| **pMB36** | *LjUbi_pro_-*HH | HH-*Lb*DR | Oligonucleotide Spacer 1 (I) for *LjUbi_pro_*_RZ (*cas12a*) | HDV-HH-*Lb*DR | Oligonucleotide Spacer 2 (I) for *LjUbi_pro_*_RZ (*cas12a*) | HDV | *PsRBCS-3A_term_* | LIIβ F 3-4 – BB24 | II | Spec | - |
| **pMB37** | *LjUbi_pro_-*HH | HH-*Lb*DR | Oligonucleotide Spacer 1 (II) for *LjUbi_pro_*_RZ (*cas12a*) | HDV-HH-*Lb*DR | Oligonucleotide Spacer 2 (II) for *LjUbi_pro_*_RZ (*cas12a*) | HDV | *PsRBCS-3A_term_* | LIIβ F 3-4 – BB24 | II | Spec | - |
|  | **AB^-^** | **A^+^E^+^** | | | | **E^+^F** | **FG** | **Backbone** | **Level** | **Res.** | **Reference** |
| **pMB38** | *LjUbi_pro_-Lb*DR | Oligonucleotide Spacer 1 (I) + Spacer 2 (I) for *LjUbi_pro__T_4_AT_6_* (*cas12a*) | | | | T_4_AT_6__*Lb*DR | *PsRBCS-3A_term_* | LIIβ F 3-4 – BB24 | II | Spec | - |
| **pMB39** | *LjUbi_pro_-Lb*DR | Oligonucleotide Spacer 1 (II) + Spacer 2 (II) for *LjUbi_pro__T_4_AT_6_* (*cas12a*) | | | | T_4_AT_6__*Lb*DR | *PsRBCS-3A_term_* | LIIβ F 3-4 – BB24 | II | Spec | - |
|  | **AB^+^** | **B^+^A^+^** | **A^+^C^+^** | **C^+^D^-^** | **D^-^C^-^** | **C^-^D^+^** | **D^+^G** | **Backbone** | **Level** | **Res.** | **Reference** |
| **pMB40** | *AtU6-26_pro_* | HH*-Lb*DR | Oligonucleotide_  Spacer 1 (I) for 2x *AtU6-26_pro__2xRZ (cas12a)* | HDV-Poly-T*-AtU6-26_pro_-*HH | HH*-Lb*DR | Oligonucleotide_  Spacer 2 (I) for 2x *AtU6-26_pro__2xRZ (cas12a)* | HDV-Poly-T | LIIβ F 3-4 – BB24 | II | Spec | - |
| **pMB41** | *AtU6-26_pro_* | HH*-Lb*DR | Oligonucleotide_  Spacer 1 (II) for 2x *AtU6-26_pro__2xRZ (cas12a)* | HDV-Poly-T*-AtU6-26_pro_-*HH | HH*-Lb*DR | Oligonucleotide_  Spacer 2 (II) for 2x *AtU6-26_pro__2xRZ (cas12a)* | HDV-Poly-T | LIIβ F 3-4 – BB24 | II | Spec | - |

| **Plasmid** | **AB** | **BC** | **CD** | **DE** | **EF** | **FG** | **Backbone** | **Level** | **Res.** | **Reference** |
| --- | --- | --- | --- | --- | --- | --- | --- | --- | --- | --- |
| **pMB43** | *35S_pro_* | SV40 | *cas12a^At D156R^* | SV40 | *NbACT3_term_* | *PsRBCS-3A_term_* | LIIβ F 2-3 - BB22 | II | Spec | - |
| **pMB44** | *LjUbi_pro_* | SV40 | *cas12a^At D156R^:NLP* | DMY | *NbACT3_term_* | *PsRBCS-3A_term_* | LIIβ F 2-3 - BB22 | II | Spec | - |
|  | **1-2** | **2-3** | **3-4** | **4-5** | **5-6** | - | **Backbone** | **Level** | **Res.** | **Reference** |
| **pMB45** | pMB1 | pMB44 | pMB29 | pMB13 | pMB15 | - | LIIIβ F A-B - BB53 | III | Kan | Figure 3a, S6, S12, S13 and S14 |
| **pMB46** | pMB1 | pMB44 | pMB32 | pMB13 | pMB15 | - | LIIIβ F A-B - BB53 | III | Kan | Figure 3a, S6, S12, S13 and S14 |
| **pMB47** | pMB1 | pMB44 | pMB34 | pMB13 | pMB15 | - | LIIIβ F A-B - BB53 | III | Kan | Figure 3a, S6, S12, S13 and S14 |
| **pMB48** | pMB1 | pMB44 | pMB36 | pMB13 | pMB15 | - | LIIIβ F A-B - BB53 | III | Kan | Figure 3a, S6, S12, S13 and S14 |
| **pMB49** | pMB1 | pMB44 | pMB38 | pMB13 | pMB15 | - | LIIIβ F A-B - BB53 | III | Kan | Figure 3a, S6, S12, S13 and S14 |
| **pMB50** | pMB1 | pMB44 | pMB40 | pMB13 | pMB15 | - | LIIIβ F A-B - BB53 | III | Kan | Figure 3a, S6, S12, S13 and S14 |
| **pMB51** | pMB1 | pMB44 | pMB30 | pMB13 | pMB18 | - | LIIIβ F A-B - BB53 | III | Kan | Figure S1, S12, S13 and S14 |
| **pMB52** | pMB1 | pMB44 | pMB33 | pMB13 | pMB18 | - | LIIIβ F A-B - BB53 | III | Kan | Figure S1, S12, S13 and S14 |
| **pMB53** | pMB1 | pMB44 | pMB35 | pMB13 | pMB18 | - | LIIIβ F A-B - BB53 | III | Kan | Figure S1, S12, S13 and S14 |
| **pMB54** | pMB1 | pMB44 | pMB37 | pMB13 | pMB18 | - | LIIIβ F A-B - BB53 | III | Kan | Figure S1, S12, S13 and S14 |
| **pMB55** | pMB1 | pMB44 | pMB39 | pMB13 | pMB18 | - | LIIIβ F A-B - BB53 | III | Kan | Figure S1, S12, S13 and S14 |
| **pMB56** | pMB1 | pMB44 | pMB41 | pMB13 | pMB18 | - | LIIIβ F A-B - BB53 | III | Kan | Figure S1, S12, S13 and S14 |
|  | **AH^+^** | **H^+^A^+^** | **A^+^A^-^** | **A^-^F** | **FG** | | **Backbone** | **Level** | **Res.** | **Reference** |
| **pMB57** | *MtU6_pro_*_*Lb*DR | *Lb*DR | Oligonucleotide Spacer 1 + 2 (I)  for *MtU6_pro__SP (Ccs12a)* | *U6-1_tem_* | DMY | | LIIβ F 3-4 – BB24 | II | Spec | - |
| **pMB58** | *AtU6MoClo_pro_*_*Lb*DR | *Lb*DR | Oligonucleotide Spacer 1 + 2 (I)  for *AtU6MoClo_pro__SP (cas12a)* | *U6-1_tem_* | DMY | | LIIβ F 3-4 – BB24 | II | Spec | - |
| **pMB59** | *AtU6-1_pro_*_*Lb*DR | *Lb*DR | Oligonucleotide Spacer 1 + 2 (I)  for *AtU6-1_pro__SP (cas12a)* | *U6-1_tem_* | DMY | | LIIβ F 3-4 – BB24 | II | Spec | - |
| **pMB60** | *AtU6-26_pro_*_*Lb*DR | *Lb*DR | Oligonucleotide Spacer 1 + 2 (I)  for *AtU6-26_pro__SP (cas12a)* | *U6-1_tem_* | DMY | | LIIβ F 3-4 – BB24 | II | Spec | - |
|  | **1-2** | **2-3** | **3-4** | **4-5** | **5-6** | - | **Backbone** | **Level** | **Res.** | **Reference** |
| **pMB61** | pMB19 | pMB43 | pMB29 | pMB14 | pMB15 | - | LIIIβ F A-B - BB53 | III | Kan | Figure 3b, S6, S12, S13 and S14 |
| **pMB62** | pMB19 | pMB43 | pMB57 | pMB14 | pMB15 | - | LIIIβ F A-B - BB53 | III | Kan | Figure 3b, S6, S12, S13 and S14 |
| **pMB63** | pMB19 | pMB43 | pMB58 | pMB14 | pMB15 | - | LIIIβ F A-B - BB53 | III | Kan | Figure 3b, S6, S12, S13 and S14 |
| **pMB64** | pMB19 | pMB43 | pMB59 | pMB14 | pMB15 | - | LIIIβ F A-B - BB53 | III | Kan | Figure 3b, S6, S12, S13 and S14 |
| **pMB65** | pMB19 | pMB43 | pMB60 | pMB14 | pMB15 | - | LIIIβ F A-B - BB53 | III | Kan | Figure 3b, S6, S12, S13 and S14 |
| **pMB66** | pMB19 | pMB43 | pMB40 | pMB14 | pMB15 | - | LIIIβ F A-B - BB53 | III | Kan | Figure 3b, 4, 6, S5, S6, S7, S9, S12, S13 and S14 |
|  | **AB** | **BC** | **CD** | **DE** | **EF** | **FG** | **Backbone** | **Level** | **Res.** | **Reference** |
| **pMB67** | *NOS_pro_* | SV40 | *cas12a^At D156R^* | SV40 | *NbACT3_term_* | *PsRBCS-3A_term_* | LIIβ F 2-3 - BB22 | II | Spec | - |
| **pMB68** | *cUbi4-2_pro_* | SV40 | *cas12a^At D156R^* | SV40 | *NbACT3_term_* | *PsRBCS-3A_term_* | LIIβ F 2-3 - BB22 | II | Spec | - |
| **pMB69** | *LjUbi_pro_* | SV40 | *cas12a^At D156R^* | SV40 | *NbACT3_term_* | *PsRBCS-3A_term_* | LIIβ F 2-3 - BB22 | II | Spec | - |
| **pMB70** | *2x35S_pro_* | SV40 | *cas12a^At D156R^* | SV40 | *NbACT3_term_* | *PsRBCS-3A_term_* | LIIβ F 2-3 - BB22 | II | Spec | - |
| **pMB71** | *35S_pro_* | SV40 | *cas12a^At D156R^* | SV40 | DMY | *PsRBCS-3A_term_* | LIIβ F 2-3 - BB22 | II | Spec | - |
| **pMB72** | *35S_pro_* | SV40 | *cas12a^At D156R^* | SV40 | *NbACT3_term_* | DMY | LIIβ F 2-3 - BB22 | II | Spec | - |
| **pMB73** | *35S_pro_* | SV40 | *cas12a^At D156R^* | SV40 | *35S_term_* | DMY | LIIβ F 2-3 - BB22 | II | Spec | - |
| **pMB74** | *35S_pro_* | SV40 | *cas12a^At D156R^* | SV40 | *35S_term_* | *PsRBCS-3A_term_* | LIIβ F 2-3 - BB22 | II | Spec | - |
|  | **1-2** | **2-3** | **3-4** | **4-5** | **5-6** | - | **Backbone** | **Level** | **Res.** | **Reference** |
| **pMB75** | pMB19 | pMB67 | pMB40 | pMB14 | pMB15 | - | LIIIβ F A-B - BB53 | III | Kan | Figure 4, S12, S13 and S14 |
| **pMB76** | pMB19 | pMB68 | pMB40 | pMB14 | pMB15 | - | LIIIβ F A-B - BB53 | III | Kan | Figure 4, S7, S12, S13 and S14 |
| **pMB77** | pMB19 | pMB69 | pMB40 | pMB14 | pMB15 | - | LIIIβ F A-B - BB53 | III | Kan | Figure 4, S7, S12, S13 and S14 |
| **pMB78** | pMB19 | pMB70 | pMB40 | pMB14 | pMB15 | - | LIIIβ F A-B - BB53 | III | Kan | Figure 4, S7, S12, S13 and S14 |
| **pMB79** | pMB19 | pMB71 | pMB40 | pMB14 | pMB15 | - | LIIIβ F A-B - BB53 | III | Kan | Figure 4, S7, S12, S13 and S14 |
| **pMB80** | pMB19 | pMB72 | pMB40 | pMB14 | pMB15 | - | LIIIβ F A-B - BB53 | III | Kan | Figure 4, S7, S12, S13 and S14 |
| **pMB81** | pMB19 | pMB73 | pMB40 | pMB14 | pMB15 | - | LIIIβ F A-B - BB53 | III | Kan | Figure 4, S7, S12, S13 and S14 |
| **pMB82** | pMB19 | pMB74 | pMB40 | pMB14 | pMB15 | - | LIIIβ F A-B - BB53 | III | Kan | Figure 4, S7, S12, S13 and S14 |
|  | **AI^+^** | **I^+^F^+^** | **I^+^F^-^** | **F^-^I^-^** | **I^-^G^-^** | **G^-^G** | **Backbone** | **Level** | **Res.** | **Reference** |
| **pMB83** | *AtU6-26_pro_ (cas9)* | Oligonucleotide_Spacer 1 (I) for 2x *AtU6-26_pro_ (cas9)* | tracr*-U6_term_* | *AtU6-26_pro_ (cas9)* | Oligonucleotide_Spacer 2 (I) for 2x *AtU6-26_pro_ (cas9)* | tracr*-U6_term_* | LIIβ F 3-4 – BB24 | II | Spec | - |
|  | **AB** | **BC** | **CD** | **DE** | **EF** | **FG** | **Backbone** | **Level** | **Res.** | **Reference** |
| **pMB89** | *35S_pro_* | SV40 | *cas12a^At^* | SV40 | *NbACT3_term_* | *PsRBCS-3A_term_* | LIIβ F 2-3 - BB22 | II | Spec | - |
| **pMB90** | *35S_pro_* | SV40 | *cas12a^Nb^* | SV40 | *NbACT3_term_* | *PsRBCS-3A_term_* | LIIβ F 2-3 - BB22 | II | Spec | - |
| **pMB91** | *35S_pro_* | SV40 | *cas12a^Lj^* | SV40 | *NbACT3_term_* | *PsRBCS-3A_term_* | LIIβ F 2-3 - BB22 | II | Spec | - |
| **pMB92** | *35S_pro_* | SV40 | *cas12a^Hs^* | SV40 | *NbACT3_term_* | *PsRBCS-3A_term_* | LIIβ F 2-3 - BB22 | II | Spec | - |
|  | **1-2** | **2-3** | **3-4** | **4-5** | **5-6** | - | **Backbone** | **Level** | **Res.** | **Reference** |
| **pMB93** | pMB19 | pMB89 | pMB40 | pMB14 | pMB15 | - | LIIIβ F A-B - BB53 | III | Kan | Figure 2, S4, S5, S12, S13 and S14 |
| **pMB94** | pMB19 | pMB90 | pMB40 | pMB14 | pMB15 | - | LIIIβ F A-B - BB53 | III | Kan | Figure 2, S4, S12, S13 and S14 |
| **pMB95** | pMB19 | pMB91 | pMB40 | pMB14 | pMB15 | - | LIIIβ F A-B - BB53 | III | Kan | Figure 2, S4, S12, S13 and S14 |
| **pMB96** | pMB19 | pMB92 | pMB40 | pMB14 | pMB15 | - | LIIIβ F A-B - BB53 | III | Kan | Figure 2, S4, S12, S13 and S14 |
|  | **AB** | **BC** | **CD** | **DE** | **EF** | **FG** | **Backbone** | **Level** | **Res.** | **Reference** |
| **pMB97** | *35S_pro_* | SV40 | *cas12a^At D156R^::introns* | SV40 | *NbACT3_term_* | *PsRBCS-3A_term_* | LIIβ F 2-3 - BB22 | II | Spec | - |
| **pMB98** | *35S_pro_* | SV40 | *cas9^Zm^::introns* | SV40 | *NbACT3_term_* | *PsRBCS-3A_term_* | LIIβ F 2-3 - BB22 | II | Spec | - |
| **pMB100** | *35S_pro_* | SV40 (*Lj*) | *cas12a^At D156R^* | SV40 (*Lj*) w.o. stop | *GFP* | *PsRBCS-3A_term_* | LIIβ F 2-3 - BB22 | II | Spec | - |
| **pMB101** | *35S_pro_* | SV40 (*Lj*) | *cas12a^At D156R^::introns* | SV40 (*Lj*) w.o. stop | *GFP* | *PsRBCS-3A_term_* | LIIβ F 2-3 - BB22 | II | Spec | - |
| **FF#23** | *LjUbi_pro_* | SV40 | *mCherry* | DMY | *NOS_term_* | DMY | LIIβ F 2-3 - BB22 | II | Spec | - |
| **F14** | *35S_pro_* | SV40 | *mCherry* | *GFP* | *35S_term_* | DMY | LIIβ F 1-2 - BB20 | II | Spec | Figure S11 |
|  | **1-2** | **2-3** | **3-4** | **4-5** | **5-6** | - | **Backbone** | **Level** | **Res.** | **Reference** |
| **pMB103** | pMB19 | pMB97 | pMB40 | pMB14 | pMB15 | - | LIIIβ F A-B - BB53 | III | Kan | Figure 6, 8, S9, S10a. S12, S13 and S14 |
| **pMB104** | pMB19 | pMB98 | pMB83 | pMB14 | pMB15 | - | LIIIβ F A-B - BB53 | III | Kan | Figure 8, S10, S12, S13 and S14 |
|  | **AB** | **BC** | **CD** | **DE** | **EF** | **FG** | **Backbone** | **Level** | **Res.** | **Reference** |
| **pMB110** | *LjUbi_pro_* | SV40 (*Lj*) | *cas12a^At D156R^* | DMY | *NbACT3_term_* | *PsRBCS-3A_term_* | LIIβ F 2-3 - BB22 | II | Spec | - |
| **pMB111** | *LjUbi_pro_* | DMY | *cas12a^At D156R^* | SV40 (*Lj*) | *NbACT3_term_* | *PsRBCS-3A_term_* | LIIβ F 2-3 - BB22 | II | Spec | - |
| **pMB112** | *LjUbi_pro_* | DMY | *cas12a^At D156R^* | DMY | *NbACT3_term_* | *PsRBCS-3A_term_* | LIIβ F 2-3 - BB22 | II | Spec | - |
| **pMB113** | *LjUbi_pro_* | c-Myc (*Lj*) | *cas12a^At D156R^* | c-Myc (*Lj*) | *NbACT3_term_* | *PsRBCS-3A_term_* | LIIβ F 2-3 - BB22 | II | Spec | - |
| **pMB114** | *LjUbi_pro_* | NLP (*Lj*) | *cas12a^At D156R^* | NLP (*Lj*) | *NbACT3_term_* | *PsRBCS-3A_term_* | LIIβ F 2-3 - BB22 | II | Spec | - |
| **pMB115** | *LjUbi_pro_* | Tus (*Lj*) | *cas12a^At D156R^* | Tus (*Lj*) | *NbACT3_term_* | *PsRBCS-3A_term_* | LIIβ F 2-3 - BB22 | II | Spec | - |
| **pMB116** | *LjUbi_pro_* | EGL-13 (*Lj*) | *cas12a^At D156R^* | EGL-13 (*Lj*) | *NbACT3_term_* | *PsRBCS-3A_term_* | LIIβ F 2-3 - BB22 | II | Spec | - |
|  | **1-2** | **2-3** | **3-4** | **4-5** | **5-6** | - | **Backbone** | **Level** | **Res.** | **Reference** |
| **pMB117** | pMB19 | pMB110 | pMB40 | pMB14 | pMB15 |  | LIIIβ F A-B - BB53 | III | Kan | Figure S2, S12, S13 and S14 |
| **pMB118** | pMB19 | pMB111 | pMB40 | pMB14 | pMB15 | **-** | LIIIβ F A-B - BB53 | III | Kan | Figure S2, S12, S13 and S14 |
| **pMB119** | pMB19 | pMB112 | pMB40 | pMB14 | pMB15 | **-** | LIIIβ F A-B - BB53 | III | Kan | Figure S2, S12, S13 and S14 |
| **pMB120** | pMB19 | pMB113 | pMB40 | pMB14 | pMB15 | **-** | LIIIβ F A-B - BB53 | III | Kan | Figure 5, S8, S12, S13 and S14 |
| **pMB121** | pMB19 | pMB114 | pMB40 | pMB14 | pMB15 | **-** | LIIIβ F A-B - BB53 | III | Kan | Figure 5, S8, S12, S13 and S14 |
| **pMB122** | pMB19 | pMB115 | pMB40 | pMB14 | pMB15 | **-** | LIIIβ F A-B - BB53 | III | Kan | Figure 5, S8, S12, S13 and S14 |
| **pMB123** | pMB19 | pMB116 | pMB40 | pMB14 | pMB15 | **-** | LIIIβ F A-B - BB53 | III | Kan | Figure 5, S8, S12, S13 and S14 |
|  | **AB** | **BC** | **CD** | **DE** | **EF** | **FG** | **Backbone** | **Level** | **Res.** | **Reference** |
| **pMB124** | *35S_pro_* | c-Myc (*Lj*) | *cas12a^At D156R^* | c-Myc (*Lj*) w.o. stop | *GFP* | *PsRBCS-3A_term_* | LIIβ F 2-3 - BB22 | II | Spec | - |
| **pMB125** | *35S_pro_* | NLP (*Lj*) | *cas12a^At D156R^* | NLP (*Lj*) w.o. stop | *GFP* | *PsRBCS-3A_term_* | LIIβ F 2-3 - BB22 | II | Spec | - |
| **pMB126** | *35S_pro_* | DMY | *cas12a^At D156R^* | *GFP* | DMY | *PsRBCS-3A_term_* | LIIβ F 2-3 - BB22 | II | Spec | - |
| **pMB127** | *35S_pro_* | DMY | *cas12a^At D156R^::introns* | *GFP* | DMY | *PsRBCS-3A_term_* | LIIβ F 2-3 - BB22 | II | Spec | - |
| **pMB128** | *35S_pro_* | NLP (*Lj*) | *cas12a^At D156R^::introns* | NLP (*Lj*) w.o. stop | *GFP* | *PsRBCS-3A_term_* | LIIβ F 2-3 - BB22 | II | Spec | - |
| **pMB129** | *35S_pro_* | NLP (*Lj*) | *cas12a^At D156R^::introns* | NLP (*Lj*) | *NbACT3_term_* | *PsRBCS-3A_term_* | LIIβ F 2-3 - BB22 | II | Spec | - |
|  | **1-2** | **2-3** | **3-4** | **4-5** | **5-6** | - | **Backbone** | **Level** | **Res.** | **Reference** |
| **pMB131** | DMY | DMY | **FF#23** | DMY | DMY | - | LIIIβ F A-B - BB53 | III | Kan | Figure 7 |
| **pMB132** | DMY | pMB100 | **FF#23** | DMY | DMY | - | LIIIβ F A-B - BB53 | III | Kan | Figure 7 |
| **pMB133** | DMY | pMB124 | **FF#23** | DMY | DMY | - | LIIIβ F A-B - BB53 | III | Kan | Figure 7 |
| **pMB134** | DMY | pMB125 | **FF#23** | DMY | DMY | - | LIIIβ F A-B - BB53 | III | Kan | Figure 7 |
| **pMB135** | DMY | pMB126 | **FF#23** | DMY | DMY | - | LIIIβ F A-B - BB53 | III | Kan | Figure 7 |
| **pMB136** | DMY | pMB127 | **FF#23** | DMY | DMY | - | LIIIβ F A-B - BB53 | III | Kan | Figure 7 |
| **pMB137** | DMY | pMB101 | **FF#23** | DMY | DMY | - | LIIIβ F A-B - BB53 | III | Kan | Figure 7 |
| **pMB138** | DMY | pMB128 | **FF#23** | DMY | DMY | - | LIIIβ F A-B - BB53 | III | Kan | Figure 7 |

**Table S3 Primers, gene strands and oligonucleotides used in this study.**

Primer sequences for amplifying a particular LI module for the GG system (Binder et al., 2014) with specific LI fusion sites, for example A-B. See table S1 for abbreviations.

| **Genotyping and sequencing** | **Primer** | **Primer Sequence 5`-3’** |
| --- | --- | --- |
|  | pro_Fw | ATGAAGACTTTACGTTTGGCTTGACCTTGATG |
|  | term_Rev | ATGAAGACTTCAGACCATACCATAGCACATACA |
| **Cloning** | **Primer** | **Primer Sequence 5`-3’** |
| *hptII*, C-D (LI) | DC-901 Hyg C-D F | ATGAAGACTTTACGGGTCTCACACCATGAAAAAGCCTGAACTCACCG |
|  | DC-902 Hyg C-D R | ATGAAGACTTCAGAGGTCTCACCTTCTATTTCTTTGCCCTCGGACGA |
| *ACT2_pro_*, A-B (L1) | DC-264 AtACT2 Pro A-B F1 | TTGAAGACATTACGGGTCTCAGCGGCCAACAAACTATTCGTCACGTCG |
|  | DC-265 AtACT2 Pro A-B R1 | TTGAAGACATTAGACTTCTTCCTTGTTCTTCTCTGTC |
|  | DC-266 AtACT2 Pro A-B F2 | TTGAAGACATTCTAAGAGAGAAAGTAAGAGATAATCC |
|  | DC-267 AtACT2 Pro A-B R2 | TTGAAGACATCAGAGGTCTCACAGATTTTTATGAGCTGCAAACACACAAAAAG |
| *mCitrine* without start codon, C-D (L1) | *mCitrine* F | ATGAAGACTTTACGGGTCTCACACCGGCGGCAGCGG |
|  | *mCitrine* R | ATGAAGACTTCAGAGGTCTCACCTTCTTGTACAGCTCGTCCATG |
| *Firefly luciferase* without start codon, C-D (L1) | *Firefly luciferase* F | ATGAAGACTTTACGGGTCTCACACCGAAGATGCAAAGAACATTA |
|  | *Firefly luciferase* R | ATGAAGACTTCAGAGGTCTCACCTTTTATAGTTTG |
| *Renilla luciferase*, C-D (L1) | DC-1262 RenLuc C-D F | ATGAAGACTTTACGGGTCTCACACCATGACGAGTAAAGTGTATGATCC |
|  | DC-1263 RenLuc C-D R | ATGAAGACTTCAGAGGTCTCACCTTTTACTGCTCATTCTTAAGAACACG |
| *NbACT3_term_*, E-F (L1) | DC-1762 NbACT3 Term E-F F1 | ATGAAGACTTTACGGGTCTCAAATCATACAGCATTCCCAGAAAGAGAA |
|  | DC-1763 NbACT3 Term E-F R1 | TAGAAGACAAGACGAAAATGAACTGACAAGCTCTTTATC |
|  | DC-1764 NbACT3 Term E-F F2 | TAGAAGACAACGTCTCATTTTTTTGGTATCCAA |
|  | DC-1765 NbACT3 Term E-F R2 | ATGAAGACTTCAGAGGTCTCACTCAATGCTAGCTTGTTTACACCTCG |
| *PsRBCS-3A_term_*, F-G (L1) | DC-944 Peas rbcs 3A E-F F | ATGAAGACTTTACGGGTCTCAAATCCAGGCCTCCCAGCTTTCGT |
|  | CD-945 Peas rbcs 3A E-F R | ATGAAGACTTCAGAGGTCTCACTCAAAGCCTATACTGTACTTAACTTGATT |
| *csy4*, C-D (L1) | *csy4* F | ATGAAGACTTTACGGGTCTCACACCATGGACCACTACCTCGACAT |
|  | *csy4* R | ATGAAGACTTCAGAGGTCTCACCTTTCAGAACCAGGGAACGAAAC |
| *cas12a^At D156R^*, C-D | *cas12a^At D156R^* p1 (L1) F | ATGAAGACTTTACGGGTCTCACACCATGAGCAAGCTCGAGAAG |
|  | *cas12a^At D156R^* p1 (L1) R | ATGAAGACTTCAGAGGTCTCAGCATCGAACAACTTCTCA |
|  | *cas12a^At D156R^* p2 (L1) F | ATGAAGACTTTACGGGTCTCAATGCTGATTTCGTCCTCG |
|  | *cas12a^At D156R^* p2 (L1) R | ATGAAGACTTCAGAGGTCTCAAGGTTTCTCTCACCACGA |
|  | *cas12a^At D156R^* p3 (L1) F | ATGAAGACTTTACGGGTCTCAACCTCCTCTACATCGTTG |
|  | *cas12a^At D156R^* p3 (L1) R | ATGAAGACTTCAGAGGTCTCACCTTGTGCTTAACGGAGGTCTG |
| *LjU6-1_pro_-Lb*DR, A-A^+^ (L1) | *LjU6-1_pro_-Lb*DR F | ATGAAGACTTTACGGGTCTCAGCGGAATCTTTGAGAGGGAATAAAAGAAA |
|  | *LjU6-1_pro_-Lb*DR R | ATGAAGACTTCAGAGGTCTCAATCTACACTTAGTAGAAATTCGAACCTTGTTGCTGA |
| *LjU6-1_pro_-*HH*,* A-B^+^ (L 1) | *LjU6-1_pro_-HH* F | ATGAAGACTTTACGGGTCTCAGCGGaaTCTTTGAGAGGG |
|  | *LjU6-1_pro_-HH* R | ATGAAGACTTCAGAGGTCTCAATTTcGAACCTTGTTGCTGAC |
| *Lb*DR-*U6-1_tem_*, A^-^-F (L1) | *Lb*DR-*U6-1_tem_* F | ATGAAGACTTTACGGGTCTCAAATTTCTACTAAGTGTAGATTTTTTTTGGCAAAAATTTTC |
|  | *Lb*DR-*U6-1_tem_* R | ATGAAGACTTCAGAGGTCTCACTCAGCTGATCCTAAATGC |
| *AtU6-26_pro_*, A-B^+^ (L1) | *AtU6-26_pro_* (A-B^+^) F | ATGAAGACTTTACGGGTCTCAGCGGCTTTTTTTCTTCTTCTTCGTTC |
|  | *AtU6-26_pro_* (A-B^+^) R | ATGAAGACTTCAGAGGTCTCAATTTCAATCACTACTTCGACTCTAGC |
| HDV-Poly-T*-AtU6-26_pro_-*HH, C^+^-D^-^ p2 (L1) | *AtU6-26_pro_* (C^+^-D^-^) p2 F | ATGAAGACTTCTAGTTCGCTTTTTTTCTTCTTCTTCGTTC |
|  | *AtU6-26_pro_* (C^+^-D^-^) p2 R | ATGAAGACTTCAGAGGTCTCAAGTAATTTCAATCACTACTTCGACTCTAGC |
| *cas12a^At D156R^:NLP*, C-D (L1) | *cas12a^At D156R^* p1 (L1) F | ATGAAGACTTTACGGGTCTCACACCATGAGCAAGCTCGAGAAG |
|  | *cas12a^At D156R^* p1(L1) R | ATGAAGACTTCAGAGGTCTCAGCATCGAACAACTTCTCA |
|  | *cas12a^At D156R^* p2(L1) F | ATGAAGACTTTACGGGTCTCAATGCTGATTTCGTCCTCG |
|  | *cas12a^At D156R^* p2 (L1) R | ATGAAGACTTCAGAGGTCTCAAGGTTTCTCTCACCACGA |
|  | *cas12a^At D156R^* p3 (L1) F | ATGAAGACTTTACGGGTCTCAACCTCCTCTACATCGTTG |
|  | *cas12a^At D156R^:NLP* (L1) R | ATGAAGACTTCAGAGGTCTCACCTTTCATTTCTTCTTCTTAGCCT |
| *cas9^At^*, C-D (L1) | *cas9^At^* p1 (L1) F | ATGAAGACTTTACGGGTCTCACACCATGGATAAGAAGTACTCTATC |
|  | *cas9^At^* p1 (L1) R | ATGAAGACTTCAGAGGTCTCAATCCAAGCGAATCTTGAG |
|  | *cas9^At^* p2(L1) F | ATGAAGACTTTACGGGTCTCAGGATGACCAGAAAGTCTG |
|  | *cas9^At^* p2 (L1) R | ATGAAGACTTCAGAGGTCTCATGCCTCTTGATGAATCCTG |
|  | *cas9^At^* p3 (L1) F | ATGAAGACTTTACGGGTCTCAGGCAGCTTGTGGAAACC |
|  | *cas9^At^* p3 (L1) R | ATGAAGACTTCAGAGGTCTCACCTTATCAGCCCTTGAATCACC |
| *GFP,* E-F (L1) | 15aaLinker:*GFP* F | ATGAAGACTTTACGGGTCTCAAATCGAGGTGGAGGAGGTTCTG |
|  | 15aaLinker:*GFP* R | ATGAAGACTTCAGAGGTCTCACTCATTACTTGTACAGCTCGTC |
| HDV-Poly-T*-LjU6-1_pro_-*HH, C^+^-D^-^  p2 (L1) | HDV-Poly-T*-LjU6-1_pro_-*HH p2 F | ATGAAGACTTCTAGTTCGaATCTTTGAGAGGGAATAAAAG |
|  | HDV-Poly-T*-LjU6-1_pro_-*HH p2 R | ATGAAGACTTCAGAGGTCTCAAGTAATTTcGAACCTTGTTGCTGAC |
| *LjUbi_pro_-*HH, A-B^+^ (L1) | *LjUbi_pro_-*HH F | ATGAAGACTTTACGGGTCTCAGCGGGGAGAGAGGATTTTGAGG |
|  | *LjUbi_pro_-*HH R | ATGAAGACTTCAGAGGTCTCAATTTCTGTAATCACATCAACAACAG |
| *LjUbi_pro_-Lb*DR, A-B^-^ (L1) | *LjUbi_pro_-*HH F | ATGAAGACTTTACGGGTCTCAGCGGGGAGAGAGGATTTTGAGG |
|  | *LjUbi_pro_-Lb*DR R | ATGAAGACTTCAGAGGTCTCAATCTACACTTAGTAGAAATTCTGTAATCACATCAACAACAG |
| *MtU6_pro_*_*Lb*DR, A-H^+^ (L1) | *MtU6_pro_*_*Lb*DR F | ATGAAGACTTTACGGGTCTCAGCGGATGCCTATCTTATA |
|  | *MtU6_pro_*_*Lb*DR R | ATGAAGACTTCAGAGGTCTCAGAAATTCAAGCCTACTGGTTCGC |
| *AtU6MoClo_pro_*_*Lb*DR, A-H^+^ (L1) | *AtU6MoClo_pro_*_*Lb*DR F | ATGAAGACTTTACGGGTCTCAGCGGTGATCAAAAGTCCC |
|  | *AtU6MoClo_pro_*_*Lb*DR R | ATGAAGACTTCAGAGGTCTCAGAAATTCAATCGCTATGTCGACTC |
| *AtU6-1_pro_*_*Lb*DR, A-H^+^ (L1) | *AtU6-1_pro_*_*Lb*DR F | ATGAAGACTTTACGGGTCTCAGCGGAGAAATCTCAAAAT |
|  | *AtU6-1_pro_*_*Lb*DR R | ATGAAGACTTCAGAGGTCTCAGAAATTCAATCACTACTTCGTGTC |
| *AtU6-26_pro_*_*Lb*DR, A-H^+^ (L1) | *AtU6-26_pro_* (A-B^+^) F | ATGAAGACTTTACGGGTCTCAGCGGCTTTTTTTCTTCTTCTTCGTTC |
|  | *AtU6-26_pro_*_*Lb*DR R | ATGAAGACTTCAGAGGTCTCAGAAATTCAATCACTACTTCGACTC |
| *PcUbi4-2_pro_,* A-B (L1) | *PcUbi4-2_pro_* F | ATGAAGACTTTACGGGTCTCAGCGGAAAAATTACGGATATGAAT |
|  | *PcUbi4-2_pro_* R | ATGAAGACTTCAGAGGTCTCACAGAGCTGCACATACATAACA |
| *2x35S_pro_*, A-B (L1) | *2x35S_pro_* p2 F | ATGAAGACTTCATGGTGGAGCACGACACACTT |
|  | *2x35S_pro_* p2 R | ATGAAGACTTTGTTATCACATCAATCCACT |
|  | *2x35S_pro_* p3 F | ATGAAGACTTAACATGGTGGAGCACGA |
|  | *2x35S_pro_* p3 R | ATGAAGACTTGGTCCTCTCCAAATGAAATG |
|  | *2x35S_pro_* p4 F | ATGAAGACTTGACCTCGAGAATTCTCA |
|  | *2x35S_pro_* p4 R | ATGAAGACTTCAGAGGTCTCACAGATGCTATCGTTCGTA |
| *cas9^At D156R^*, C-D (L1) | *cas9^At D156R^* p1 (L1) a F | ATGAAGACTTTACGGGTCTCACACCatgAGCAAGCTCGAGAAG |
|  | *cas9^At D156R^* p1 (L1) a R | ATGAAGACTTGTCGAAGAATCCGGTGAAGG |
|  | *cas9^At D156R^* p1 (L1) b F | ATGAAGACTTCGACAACAGGGAAAACATGTTCAG |
|  | *cas9^At D156R^* p1 (L1) b R | ATGAAGACTTCAGAGGTCTCAGCATCGAACAACTTCTCA |
|  | *cas9^At D156R^* p2(L1) F | ATGAAGACTTTACGGGTCTCAATGCTGATTTCGTCCTCG |
|  | *cas9^At D156R^* p2 (L1) R | ATGAAGACTTCAGAGGTCTCAAGGTTTCTCTCACCACGA |
|  | *cas9^At D156R^* p3 (L1) F | ATGAAGACTTTACGGGTCTCAACCTCCTCTACATCGTTG |
|  | *cas9^At D156R^* p3 (L1) R | ATGAAGACTTCAGAGGTCTCACCTTGTGCTTAACGGAGGTCTG |
| *cas12a^Nb^*, C-D (L1) | *cas12a^Nb^* p1 (L1) F | ATGAAGACTTTACGGGTCTCACACCATGAGCAAGCTCGAGAAG |
|  | *cas12a^Nb^* p1(L1) R | ATGAAGACTTCAGAGGTCTCAAGCGTCAGCATACTCTTG |
|  | *cas12a^Nb^* p2 (L1) F | ATGAAGACTTTACGGGTCTCACGCTGATCTGTCCGTGG |
|  | *cas12a^Nb^* p2 (L1) R | ATGAAGACTTCAGAGGTCTCACACTTGTTGATGGCGATC |
|  | *cas12a^Nb^* p3 (L1) F | ATGAAGACTTTACGGGTCTCAAGTGCCCGAAGAACATC |
|  | *cas12a^Nb^* p3 (L1) R | ATGAAGACTTCAGAGGTCTCACCTTATGCTTAACGCTAGTCTG |
| *cas12a^Hs^*, C-D (L1) | *cas12a^Hs^* p1(L1) F | ATGAAGACTTTACGGGTCTCACACCATGAGCAAGCTGGAGAAG |
|  | *cas12a^Hs^* p1(L1) R | ATGAAGACTTCAGAGGTCTCACTTCTCCACCACAGACAG |
|  | *cas12a^Hs^* p2(L1) F | ATGAAGACTTTACGGGTCTCAGAAGCTGAAGGAGATCATC |
|  | *cas12a^Hs^* p2 (L1) R | ATGAAGACTTCAGAGGTCTCATTGATCTTGAAGATGTTCTT |
|  | *cas12a^Hs^* p3(L1) F | ATGAAGACTTTACGGGTCTCATCAATACAGAGGTGCGC |
|  | *cas12a^Hs^* p3 (L1) R | ATGAAGACTTCAGAGGTCTCACCTTGTGCTTCACGCTGGTCT |
| *cas9^Zm^::introns*, C-D | *cas9^Zm^::introns* p1 (L1) F | ATGAAGACTTTACGGGTCTCACACCATGGACAAGAAGTACAGCAT |
|  | *cas9^Zm^::introns* p1(L1) R | ATGAAGACTTCAGAGGTCTCACCTGCGTAGCCATTCTTC |
|  | *cas9^Zm^::introns* p2 (L1) F | ATGAAGACTTTACGGGTCTCACAGGGTACATTGATGGAG |
|  | *cas9^Zm^::introns* p2 (L1) R | ATGAAGACTTCAGAGGTCTCAGTCCAGTATAGTCTTGCC |
|  | *cas9^Zm^::introns* p3 (L1) F | ATGAAGACTTTACGGGTCTCAGGACTTTCTCAAATCCGAC |
|  | *cas9^Zm^::introns* p3 (L1) R | ATGAAGACTTCAGAGGTCTCATCTTCCTAACGTCGTACAC |
|  | *cas9^Zm^::introns* p4 (L1) F | ATGAAGACTTTACGGGTCTCAAAGATGATCGCGAAGTC |
|  | *cas9^Zm^::introns* p4 (L1) R | ATGAAGACTTCAGAGGTCTCACCTTGTCAGCCCTAGAGTCACC |
| *AtU6-26_pro_ (cas9)*, A-I^+^ (L1) | *AtU6-26_pro_* (A-B^+^) F | ATGAAGACTTTACGGGTCTCAGCGGCTTTTTTTCTTCTTCTTCGTTC |
|  | *AtU6-26_pro_ (cas9)*, A-I^+^ R | ATGAAGACTTCAGAGGTCTCACAATCACTACTTCGACTC |
| *AtU6-26_pro_ (cas9)*, F^-^-I^-^ (L1) | *AtU6-26_pro_ (cas9)*, F^-^-I^-^ F | ATGAAGACTTTACGGGTCTCAAGCTCTTTTTTTCTTCTTCTTCGTTC |
|  | *AtU6-26_pro_ (cas9)*, F^-^-I^-^ R | ATGAAGACTTCAGAGGTCTCACACTACTTCGACTCTAGC |
| tracr*-U6_term_*, F^+^-F^-^ (L1) | tracr*-U6_term_* (F^+^-F^-^) F | ATGAAGACTTTACGGGTCTCATAGAGCTAGAAATAGCAAG |
|  | tracr*-U6_term_* (F^+^-F^-^) F | ATGAAGACTTCAGAGGTCTCAAGCTTAGGTCAGGTGGCTGATCCTAAATGCTATC |
| tracr*-U6_term_*, G^-^-G (L1) | tracr*-U6_term_* (G^-^-G) F | ATGAAGACTTTACGGGTCTCAGTTTTAGAGCTAGAAATAG |
|  | tracr*-U6_term_* (G^-^-G) R | ATGAAGACTTCAGAGGTCTCAGACAGCTGATCCTAAATGC |

| **Cloning** | **Gene strand** | **Segment of gene strand Sequence 5`-3’** |
| --- | --- | --- |
| *cas12a^Lj^*, C-D  for complete sequence see Appendix S16 | *cas12a^Lj^* p1 (L1) 5’ region | ATGAAGACTTTACGGGTCTCACACCATGAGCAAGTTAGAGAAATTC… |
|  | *cas12a^Lj^* p1(L1) 3’ region | ATGAAGACTTCAGAGGTCTCACGATCAACTTATGACAATC… |
|  | *cas12a^Lj^* p2 (L1) F 5’ region | ATGAAGACTTTACGGGTCTCAATCGATTTCTTTAAGGACTC… |
|  | *cas12a^Lj^* p2 (L1) R 3’ region | ATGAAGACTTCAGAGGTCTCACCTTATGTTTAACAGAAGTCTGTG… |
| *cas12a^At D156R^::introns*, C-D  for complete sequence see Appendix S17 | *cas12a^At D156R^::introns* p1 (L1) 5’ region | ATGAAGACTTTACGGGTCTCACACCATGAGCAAGCTCGAGAAGTTTAC… |
|  | *cas12a^At D156R^::introns* p1 (L1) 3’ region | ATGAAGACTTCAGAGGTCTCACACGAAATCCCCGTAGAAAGACTC… |
|  | *cas12a^At D156R^::introns* p2 (L1) 5’ region | ATGAAGACTTTACGGGTCTCACGTGCTCGCTTACGATATC… |
|  | *cas12a^At D156R^::introns* p2 (L1) 3’ region | ATGAAGACTTCAGAGGTCTCAAAAAATTATCGACCTTTTAC… |
|  | *cas12a^At D156R^::introns* p3 a (L1) 5’ region | ATGAAGACTTTACGGGTCTCATTTTTTTTGGAGCCATTATG… |
|  | *cas12a^At D156R^::introns* p3 a (L1) 3’ region | ATGAAGACTTCCGTTTGCATCAGCATTCTTCG… |
|  | *cas12a^At D156R^::introns* p3 b (L1) 5’ region | ATGAAGACTTACGGGGCTTACAACATTGCG… |
|  | *cas12a^At D156R^::introns* p3 b (L1) 3’ region | ATGAAGACTTCAGAGGTCTCACCTTGTGCTTAACGGAGGTCTG… |

| **Cloning** | **Oligonucleotide**  **(+) strand / (-) strand** | **Oligonucleotide Sequence 5`-3’** |
| --- | --- | --- |
| *ATG:HP*, B-C (LI) | *ATG:HP* (+) | ATGAAGACTTTACGGGTCTCATCTGAACAATGGGCAGCCACCACCACCACCACCACAGCGGAATGAGTTCACTGCCGTATAGGCAGCTAAGAAAATCAGGTGGCACCTGAGACCTCTGAAGTCTTCAT |
|  | *ATG:HP* (-) | ATGAAGACTTCAGAGGTCTCAGGTGCCACCTGATTTTCTTAGCTGCCTATACGGCAGTGAACTCATTCCGCTGTGGTGGTGGTGGTGGTGGCTGCCCATTGTTCAGATGAGACCCGTAAAGTCTTCAT |
| 5‘ target (I), B-C (L1) | 5‘ target (I) (+) | ATGAAGACTTTACGGGTCTCATCTGCCCACGACTCACATAGTCCCCCTCAAACACCTGAGACCTCTGAAGTCTTCAT |
|  | 5‘ target (I) (-) | ATGAAGACTTCAGAGGTCTCAGGTGTTTGAGGGGGACTATGTGAGTCGTGGGCAGATGAGACCCGTAAAGTCTTCAT |
| 3‘ target (I), D-E (L1) | 3‘ target (I) (+) | ATGAAGACTTTACGGGTCTCAAAGGTTTGATGAGTACCGGAATCACCAGGGGAATCTGAGACCTCTGAAGTCTTCAT |
|  | 3‘ target (I) (-) | ATGAAGACTTCAGAGGTCTCAGATTCCCCTGGTGATTCCGGTACTCATCAAACCTTTGAGACCCGTAAAGTCTTCAT |
| 5‘ target (II), B-C (L1) | 5‘ target (II) (+) | ATGAAGACTTTACGGGTCTCATCTGCCTTGGACTCTCAAATTAACGGCGAAACACCTGAGACCTCTGAAGTCTTCAT |
|  | 5‘ target (II) (-) | ATGAAGACTTCAGAGGTCTCAGGTGTTTCGCCGTTAATTTGAGAGTCCAAGGCAGATGAGACCCGTAAAGTCTTCAT |
| 5‘ target (II), D-E (L1) | 3‘ target (II) (+) | ATGAAGACTTTACGGGTCTCAAAGGTTTCACGTATCTTCGGCCATGAAGAGGAATCTGAGACCTCTGAAGTCTTCAT |
|  | 3‘ target (II) (-) | ATGAAGACTTCAGAGGTCTCAGATTCCTCTTCATGGCCGAAGATACGTGAAACCTTTGAGACCCGTAAAGTCTTCAT |
| SV40 (*Lj*), B-C (L1) | N-SV40 (*Lj*) (+) | ATGAAGACTTTACGGGTCTCATCTGAACAATGCTTCAACCTAAGAAGAAGAGAAAGGTTGGAGGAAACACCTGAGACCTCTGAAGTCTTCAT |
|  | N-SV40 (*Lj*) (-) | ATGAAGACTTCAGAGGTCTCAGGTGTTTCCTCCAACCTTTCTCTTCTTCTTAGGTTGAAGCATTGTTCAGATGAGACCCGTAAAGTCTTCAT |
| SV40 (*Lj*), D-E (L1) | C-SV40 (*Lj*) (+) | ATGAAGACTTTACGGGTCTCAAAGGGACCTAAGAAGAAGAGAAAGGTTTGAAATCTGAGACCTCTGAAGTCTTCAT |
|  | C-SV40 (*Lj*) (-) | ATGAAGACTTCAGAGGTCTCAGATTTCAAACCTTTCTCTTCTTCTTAGGTCCCTTTGAGACCCGTAAAGTCTTCAT |
| c-Myc (*Lj*), B-C (L1) | N-c-Myc (*Lj*) (+) | ATGAAGACTTTACGGGTCTCATCTGAACAATGCTTCAACCTGCTGCTAAGAGAGTTAAGCTTGATGGAGGAAACACCTGAGACCTCTGAAGTCTTCAT |
|  | N-c-Myc (*Lj*) (-) | ATGAAGACTTCAGAGGTCTCAGGTGTTTCCTCCATCAAGCTTAACTCTCTTAGCAGCAGGTTGAAGCATTGTTCAGATGAGACCCGTAAAGTCTTCAT |
| c-Myc (*Lj*), B-C (L1) | C-c-Myc (*Lj*) (+) | ATGAAGACTTTACGGGTCTCAAAGGGACCTGCTGCTAAGAGAGTTAAGCTTGATTGAAATCTGAGACCTCTGAAGTCTTCAT |
|  | C-c-Myc (*Lj*) (-) | ATGAAGACTTCAGAGGTCTCAGATTTCAATCAAGCTTAACTCTCTTAGCAGCAGGTCCCTTTGAGACCCGTAAAGTCTTCAT |
| NLP (*Lj*), B-C (L1) | N-NLP (*Lj*) (+) | ATGAAGACTTTACGGGTCTCATCTGAACAATGCTTCAAAAGAGGCCTGCTGCTACTAAGAAGGCTGGTCAAGCTAAGAAGAAGAAGGGAGGAAACACCTGAGACCTCTGAAGTCTTCAT |
|  | N-NLP (*Lj*) (-) | ATGAAGACTTCAGAGGTCTCAGGTGTTTCCTCCCTTCTTCTTCTTAGCTTGACCAGCCTTCTTAGTAGCAGCAGGCCTCTTTTGAAGCATTGTTCAGATGAGACCCGTAAAGTCTTCAT |
| NLP (*Lj*), B-C (L1) | C-NLP (*Lj*) (+) | ATGAAGACTTTACGGGTCTCAAAGGGAAAGAGGCCTGCTGCTACTAAGAAGGCTGGTCAAGCTAAGAAGAAGAAGTGAAATCTGAGACCTCTGAAGTCTTCAT |
|  | C-NLP (*Lj*) (-) | ATGAAGACTTCAGAGGTCTCAGATTTCACTTCTTCTTCTTAGCTTGACCAGCCTTCTTAGTAGCAGCAGGCCTCTTTCCCTTTGAGACCCGTAAAGTCTTCAT |
| Tus (*Lj*), B-C (L1) | N-Tus (*Lj*) (+) | ATGAAGACTTTACGGGTCTCATCTGAACAATGCTTCAAAAGCTTAAGATTAAGAGGCCTGTTAAGGGAGGAAACACCTGAGACCTCTGAAGTCTTCAT |
|  | N-Tus (*Lj*) (-) | ATGAAGACTTCAGAGGTCTCAGGTGTTTCCTCCCTTAACAGGCCTCTTAATCTTAAGCTTTTGAAGCATTGTTCAGATGAGACCCGTAAAGTCTTCAT |
| Tus (*Lj*), B-C (L1) | C-Tus (*Lj*) (+) | ATGAAGACTTTACGGGTCTCAAAGGGAAAGCTTAAGATTAAGAGGCCTGTTAAGTGAAATCTGAGACCTCTGAAGTCTTCAT |
|  | C-Tus (*Lj*) (-) | ATGAAGACTTCAGAGGTCTCAGATTTCACTTAACAGGCCTCTTAATCTTAAGCTTTCCCTTTGAGACCCGTAAAGTCTTCAT |
| EGL-13 (*Lj*), B-C (L1) | N-EGL-13 (*Lj*) p1 (+) | ATGAAGACTTTACGGGTCTCATCTGAACAATGCTTCAAATGTCTAGAAGAAGAAAGGCTAATCCTACTAAGCTTTCTGAAAGTCTTCAT |
|  | N-EGL-13 (*Lj*) p1 (-) | ATGAAGACTTTCAGAAAGCTTAGTAGGATTAGCCTTTCTTCTTCTAGACATTTGAAGCATTGTTCAGATGAGACCCGTAAAGTCTTCAT |
|  | N-EGL-13 (*Lj*) p2 (+) | ATGAAGACTTCTGAGAATGCTAAGAAGCTTGCTAAGGAGGTTGAGAATGGAGGAAACACCTGAGACCTCTGAAGTCTTCAT |
|  | N-EGL-13 (*Lj*) p2 (-) | ATGAAGACTTCAGAGGTCTCAGGTGTTTCCTCCATTCTCAACCTCCTTAGCAAGCTTCTTAGCATTCTCAGAAGTCTTCAT |
| EGL-13 (*Lj*), B-C (L1) | C-EGL-13 (*Lj*) p1 (+) | ATGAAGACTTTACGGGTCTCAAAGGGAATGTCTAGAAGAAGAAAGGCTAATCCTACTAAGCTTTCTGAAAGTCTTCAT |
|  | C-EGL-13 (*Lj*) p1 (-) | ATGAAGACTTTCAGAAAGCTTAGTAGGATTAGCCTTTCTTCTTCTAGACATTCCCTTTGAGACCCGTAAAGTCTTCAT |
|  | C-EGL-13 (*Lj*) p2 (+) | ATGAAGACTTCTGAGAATGCTAAGAAGCTTGCTAAGGAGGTTGAGAATTGAAATCTGAGACCTCTGAAGTCTTCAT |
|  | C-EGL-13 (*Lj*) p2 (-) | ATGAAGACTTCAGAGGTCTCAGATTTCAATTCTCAACCTCCTTAGCAAGCTTCTTAGCATTCTCAGAAGTCTTCAT |
| SV40 (*Lj*) w.o. stop, D-E (L1) | SV40 (*Lj*) w.o. stop (+) | ATGAAGACTTTACGGGTCTCAAAGGGACCTAAGAAGAAGAGAAAGGTTAATCTGAGACCTCTGAAGTCTTCAT |
|  | SV40 (*Lj*) w.o. stop (-) | ATGAAGACTTCAGAGGTCTCAGATTAACCTTTCTCTTCTTCTTAGGTCCCTTTGAGACCCGTAAAGTCTTCAT |
| c-Myc (*Lj*) w.o. stop, D-E (L1) | c-Myc (*Lj*) w.o. stop (+) | ATGAAGACTTTACGGGTCTCAAAGGGACCTGCTGCTAAGAGAGTTAAGCTTGATAATCTGAGACCTCTGAAGTCTTCAT |
|  | c-Myc (*Lj*) w.o. stop (-) | ATGAAGACTTCAGAGGTCTCAGATTATCAAGCTTAACTCTCTTAGCAGCAGGTCCCTTTGAGACCCGTAAAGTCTTCAT |
| NLP (*Lj*) w.o. stop, D-E (L1) | NLP (*Lj*) w.o. stop (+) | ATGAAGACTTTACGGGTCTCAAAGGGAAAGAGGCCTGCTGCTACTAAGAAGGCTGGTCAAGCTAAGAAGAAGAAGAATCTGAGACCTCTGAAGTCTTCAT |
|  | NLP (*Lj*) w.o. stop (-) | ATGAAGACTTCAGAGGTCTCAGATTCTTCTTCTTCTTAGCTTGACCAGCCTTCTTAGTAGCAGCAGGCCTCTTTCCCTTTGAGACCCGTAAAGTCTTCAT |
| Oligonucleotide Spacer 1 + 2 (I) for *LjU6_pro__SP (cas12a), MtU6_pro__SP (cas12a)*, *AtU6MoClo_pro__SP (cas12a), AtU6-1_pro__SP (cas12a) and AtU6-26_pro__SP (cas12a)* A^+^-A^-^ (L1) | Spacer 1 + 2 (I) for *SP_cas12a* (+) | ATGAAGACTTTACGGGTCTCAAGATAGGGGGACTATGTGAGTCGTAATTTCTACTAAGTGTAGATATGAGTACCGGAATCACCAGAATTTGAGACCTCTGAAGTCTTCAT |
|  | Spacer 1 + 2 (I) for *SP_cas12a*  (-) | ATGAAGACTTCAGAGGTCTCAAATTCTGGTGATTCCGGTACTCATATCTACACTTAGTAGAAATTACGACTCACATAGTCCCCCTATCTTGAGACCCGTAAAGTCTTCAT |
| Oligonucleotide Spacer 1 + 2 (II) for *LjU6_pro__SP (cas12a), MtU6_pro__SP (cas12a)*, *AtU6MoClo_pro__SP (cas12a), AtU6-1_pro__SP (cas12a) and AtU6-26_pro__SP (cas12a)* A^+^-A^-^ (L1) | Spacer 1 + 2 (II) for *SP_cas12a* (+) | ATGAAGACTTTACGGGTCTCAAGATGCCGTTAATTTGAGAGTCCAAATTTCTACTAAGTGTAGATACGTATCTTCGGCCATGAAGTGAGACCTCTGAAGTCTTCAT |
|  | Spacer 1 + 2 (II) for *SP_cas12a* (-) | ATGAAGACTTCAGAGGTCTCACTTCATGGCCGAAGATACGTATCTACACTTAGTAGAAATTTGGACTCTCAAATTAACGGCATCTTGAGACCCGTAAAGTCTTCAT |
| HH*-Lb*DR, B^+^-A^+^ (L1) | HH*-Lb*DR (+) | ATGAAGACTTTACGGGTCTCAAAATTACTGATGAGTCCGTGAGGACGAAACGAGTAAGCTCGTCTAATTTCTACTAAGTGTAGATTGAGACCTCTGAAGTCTTCAT |
|  | HH*-Lb*DR (-) | ATGAAGACTTCAGAGGTCTCAATCTACACTTAGTAGAAATTAGACGAGCTTACTCGTTTCGTCCTCACGGACTCATCAGTAATTTTGAGACCCGTAAAGTCTTCAT |
| HDV-HH-*Lb*DR, C^+^-C^-^ (L1) | HDV-HH-*Lb*DR p1 (+) | ATGAAGACTTTACGGGTCTCAGGCATGGTCCCAGCCTCCTCGCTGGCGCCGGCTGGGCAACATGCTTCGGCATGGCGAATGGGACTGACGCTAGCCTAGTTCGAAATAAGTCTTCAT |
|  | HDV-HH-*Lb*DR p1 (-) | ATGAAGACTTATTTCGAACTAGGCTAGCGTCAGTCCCATTCGCCATGCCGAAGCATGTTGCCCAGCCGGCGCCAGCGAGGAGGCTGGGACCATGCCTGAGACCCGTAAAGTCTTCAT |
|  | HDV-HH-*Lb*DR p2 (+) | ATGAAGACTTAAATTACTGATGAGTCCGTGAGGACGAAACGAGTAAGCTCGTCTAATTTCTACTAAGTGTTGAGACCTCTGAAGTCTTCAT |
|  | HDV-HH-*Lb*DR p2 (-) | ATGAAGACTTCAGAGGTCTCAACACTTAGTAGAAATTAGACGAGCTTACTCGTTTCGTCCTCACGGACTCATCAGTAATTTAAGTCTTCAT |
| HDV-Poly-T, D^+^-G (L1) | HDV-Poly-T (+) | ATGAAGACTTTACGGGTCTCAGGCCGGCATGGTCCCAGCCTCCTCGCTGGCGCCGGCTGGGCAACATGCTTCGGCATGGCGAATGGGACTTTTTTGTCTGAGACCTCTGAAGTCTTCAT |
|  | HDV-Poly-T (-) | ATGAAGACTTCAGAGGTCTCAGACAAAAAAGTCCCATTCGCCATGCCGAAGCATGTTGCCCAGCCGGCGCCAGCGAGGAGGCTGGGACCATGCCGGCCTGAGACCCGTAAAGTCTTCAT |
| HDV-Poly-T*-LjU6-1_pro_-*HH, C^+^-D^-^  p1 (L1) | HDV-Poly-T*-LjU6-1_pro_-*HH p1 (+) | ATGAAGACTTTACGGGTCTCAGGCATGGTCCCAGCCTCCTCGCTGGCGCCGGCTGGGCAACATGCTTCGGCATGGCGAATGGGACTTTTTTGACGCTAGCCTAGAAGTCT |
|  | HDV-Poly-T*-LjU6-1_pro_-*HH p1 (-) | AGACTTCTAGGCTAGCGTCAAAAAAGTCCCATTCGCCATGCCGAAGCATGTTGCCCAGCCGGCGCCAGCGAGGAGGCTGGGACCATGCCTGAGACCCGTAAAGTCTTCAT |
| HH*-Lb*DR, D^-^-C^-^ (L1) | HH*-Lb*DR, D^-^-C^-^ (+) | ATGAAGACTTTACGGGTCTCATACTGATGAGTCCGTGAGGACGAAACGAGTAAGCTCGTCTAATTTCTACTAAGTGTTGAGACCTCTGAAGTCTTCAT |
|  | HH*-Lb*DR, D^-^-C^-^ (-) | ATGAAGACTTCAGAGGTCTCAACACTTAGTAGAAATTAGACGAGCTTACTCGTTTCGTCCTCACGGACTCATCAGTATGAGACCCGTAAAGTCTTCAT |
| Oligonucleotide Spacer 1 (I) for *LjUbi_pro__RZ* *(cas12a)*, *LjU6_pro__RZ* *(cas12a),* 2x *LjU6_pro__2xRZ* *(cas12a)* and 2x *AtU6-26_pro__2xRZ (cas12a)*, A^+^-C^+^ (L1) | Spacer 1 (I) for *RZ_cas12a* (+) | ATGAAGACTTTACGGGTCTCAAGATAGGGGGACTATGTGAGTCGTGGCCGGCATGAGACCTCTGAAGTCTTCAT |
|  | Spacer 1 (I) for *RZ_cas12a* (-) | ATGAAGACTTCAGAGGTCTCATGCCGGCCACGACTCACATAGTCCCCCTATCTTGAGACCCGTAAAGTCTTCAT |
| Oligonucleotide Spacer 1 (II) for *LjUbi_pro__RZ* *(cas12a)*, *LjU6_pro__RZ* *(cas12a),* 2x *LjU6_pro__2xRZ* *(cas12a)* and 2x *AtU6-26_pro__2xRZ (cas12a)*, A^+^-C^+^ (L1) | Spacer 1 (II) for *RZ_cas12a* (+) | ATGAAGACTTTACGGGTCTCAAGATGCCGTTAATTTGAGAGTCCAGGCCGGCATGAGACCTCTGAAGTCTTCAT |
|  | Spacer 1 (II) for *RZ_cas12a* (-) | ATGAAGACTTCAGAGGTCTCATGCCGGCCTGGACTCTCAAATTAACGGCATCTTGAGACCCGTAAAGTCTTCAT |
| HDV, D^+^-F (L1) | HDV (+) | ATGAAGACTTTACGGGTCTCAGGCCGGCATGGTCCCAGCCTCCTCGCTGGCGCCGGCTGGGCAACATGCTTCGGCATGGCGAATGGGACTGAGTGAGACCTCTGAAGTCTTCAT |
|  | HDV (-) | ATGAAGACTTCAGAGGTCTCACTCAGTCCCATTCGCCATGCCGAAGCATGTTGCCCAGCCGGCGCCAGCGAGGAGGCTGGGACCATGCCGGCCTGAGACCCGTAAAGTCTTCAT |
| Oligonucleotide Spacer 1 (I) + Spacer 2 (I) for *LjUbi_pro__T_4_AT_6_* (*cas12a*) | Spacer 1 + 2 (I) for *LjUbi_pro__T_4_AT_6_* (*cas12a*) (+) | ATGAAGACTTTACGGGTCTCAAGATAGGGGGACTATGTGAGTCGTTTTTATTTTTTAATTTCTACTAAGTGTAGATATGAGTACCGGAATCACCAGTTTTTGAGACCTCTGAAGTCTTCA |
|  | Spacer 1 + 2 (I) for *LjUbi_pro__T_4_AT_6_* (*cas12a*) (-) | TGAAGACTTCAGAGGTCTCAAAAACTGGTGATTCCGGTACTCATATCTACACTTAGTAGAAATTAAAAAATAAAAACGACTCACATAGTCCCCCTATCTTGAGACCCGTAAAGTCTTCAT |
| Oligonucleotide Spacer 1 (II) + Spacer 2 (II) for *LjUbi_pro__T_4_AT_6_* (*cas12a*) | Spacer 1 + 2 (II) for *LjUbi_pro__T_4_AT_6_* (*cas12a*) (+) | ATGAAGACTTTACGGGTCTCAAGATGCCGTTAATTTGAGAGTCCATTTTATTTTTTAATTTCTACTAAGTGTAGATACGTATCTTCGGCCATGAAGTTTTTGAGACCTCTGAAGTCTTCA |
|  | Spacer 1 + 2 (II) for *LjUbi_pro__T_4_AT_6_* (*cas12a*) (-) | TGAAGACTTCAGAGGTCTCAAAAACTTCATGGCCGAAGATACGTATCTACACTTAGTAGAAATTAAAAAATAAAATGGACTCTCAAATTAACGGCATCTTGAGACCCGTAAAGTCTTCAT |
| T_4_AT_6__*Lb*DR, E^+^-F (L1) | T_4_AT_6__*Lb*DR (+) | ATGAAGACTTTACGGGTCTCATTTTATTTTTTAATTTCTACTAAGTGTAGATTGAGTGAGACCTCTGAAGTCTTCAT |
|  | T_4_AT_6__*Lb*DR (-) | ATGAAGACTTCAGAGGTCTCACTCAATCTACACTTAGTAGAAATTAAAAAATAAAATGAGACCCGTAAAGTCTTCAT |
| HDV-Poly-T*-AtU6-26_pro_-*HH, C^+^-D^-^ p1 (L1) | HDV-Poly-T*-AtU6-26_pro_-*HH p1 (+) | ATGAAGACTTTACGGGTCTCAGGCATGGTCCCAGCCTCCTCGCTGGCGCCGGCTGGGCAACATGCTTCGGCATGGCGAATGGGACTTTTTTGACGCTAGCCTAGAAGTCTTCAT |
|  | HDV-Poly-T*-AtU6-26_pro_-*HH p1 (-) | ATGAAGACTTCTAGGCTAGCGTCAAAAAAGTCCCATTCGCCATGCCGAAGCATGTTGCCCAGCCGGCGCCAGCGAGGAGGCTGGGACCATGCCTGAGACCCGTAAAGTCTTCAT |
| Oligonucleotide_  Spacer 2 (I) for *LjUbi_pro__RZ* *(cas12a)*, *LjU6_pro__RZ* *(cas12a),* 2x *LjU6_pro__2xRZ* *(cas12a)* and 2x *AtU6-26_pro__2xRZ (cas12a)*, A^+^-C^+^ (L1) | Spacer 2 (I) for *RZ_cas12a* (+) | ATGAAGACTTTACGGGTCTCAGTGTAGATATGAGTACCGGAATCACCAGGGCCTGAGACCTCTGAAGTCTTCAT |
|  | Spacer 2 (I) for *RZ_cas12a* (-) | ATGAAGACTTCAGAGGTCTCAGGCCCTGGTGATTCCGGTACTCATATCTACACTGAGACCCGTAAAGTCTTCAT |
| Oligonucleotide_  Spacer 2 (II) for *LjUbi_pro__RZ* *(cas12a)*, *LjU6_pro__RZ* *(cas12a),* 2x *LjU6_pro__2xRZ* *(cas12a)* and 2x *AtU6-26_pro__2xRZ (cas12a)*, A^+^-C^+^ (L1) | Spacer 2 (II) for *RZ_cas12a* (+) | ATGAAGACTTTACGGGTCTCAGTGTAGATACGTATCTTCGGCCATGAAGggccTGAGACCTCTGAAGTCTTCAT |
|  | Spacer 2 (II) for *RZ_cas12a* (-) | ATGAAGACTTCAGAGGTCTCAGGCCCTTCATGGCCGAAGATACGTATCTACACTGAGACCCGTAAAGTCTTCAT |
| *Lb*DR, H^+^-A^+^ (L1) | *Lb*DR (+) | ATGAAGACTTTACGGGTCTCATTTCTACTAAGTGTAGATTGAGACCTCTGAAGTCTTCAT |
|  | *Lb*DR (-) | ATGAAGACTTCAGAGGTCTCAATCTACACTTAGTAGAAATGAGACCCGTAAAGTCTTCAT |
| DMY, E-F (L1) | DMY, E-F (+) | ATGAAGACTTTACGGGTCTCAAATCTGACCTAAGCTAGCCTAGTGAGTGAGACCTCTGAAGTCTTCAT |
|  | DMY, E-F (-) | ATGAAGACTTCAGAGGTCTCACTCACTAGGCTAGCTTAGGTCAGATTTGAGACCCGTAAAGTCTTCAT |
| *2x35S_pro_*, A-B (L1) | *2x35S_pro_* p1 (+) | ATGAAGACTTTACGGGTCTCAGCGGTTGCGGCAGCGTGAAGCTTGCATGCCTGCAGGTCAACATGAAGTCTTCAT |
|  | *2x35S_pro_* p1 (-) | ATGAAGACTTCATGTTGACCTGCAGGCATGCAAGCTTCACGCTGCCGCAACCGCTGAGACCCGTAAAGTCTTCAT |
| Oligonucleotide_  Spacer 1 (I) for 2x *AtU6-26_pro_ (cas9)*, I^+^-F^+^ (L1) | Spacer 1 (I) for 2x *AtU6-26_pro_ (cas9)* (+) | ATGAAGACTTTACGGGTCTCAATTGAGGGGGACTATGTGAGTCGTgttttagaTGAGACCTCTGAAGTCTTCAT |
|  | Spacer 1 (I) for 2x *AtU6-26_pro_ (cas9)* (-) | ATGAAGACTTCAGAGGTCTCATCTAAAACACGACTCACATAGTCCCCCTCAATTGAGACCCGTAAAGTCTTCAT |
| Oligonucleotide_  Spacer 2 (I) for 2x *AtU6-26_pro_ (cas9)*, I^-^-G^-^ (L1) | Spacer 2 (I) for 2x *AtU6-26_pro_ (cas9)* (+) | ATGAAGACTTTACGGGTCTCAAGTGATTGATGAGTACCGGAATCACCAGgtttTGAGACCTCTGAAGTCTTCAT |
|  | Spacer 2 (I) for 2x *AtU6-26_pro_ (cas9)* (-) | ATGAAGACTTCAGAGGTCTCAAAACCTGGTGATTCCGGTACTCATCAATCACTTGAGACCCGTAAAGTCTTCAT |

**Table S4 Spacer sequences used in this study.**

| Incorporation in crRNA and sgRNA expression cassettes for targeting 5’ and 3’ targets in the *csy4* expression cassette of version (I) or (II) | **Spacer 1** | **Spacer 2** |
| --- | --- | --- |
| **(I)** | 5‘ AGGGGGACTATGTGAGTCGT 3‘ | 5’ ATGAGTACCGGAATCACCAG 3‘ |
| **(II)** | 5‘ GCCGTTAATTTGAGAGTCCA 3‘ | 5’ ACGTATCTTCGGCCATGAAG 3‘ |

**Data S1** Sequences of plasmids. RAR archive of annotated maps of plasmids (Genbank format).

Sequences of annotated level II and level III plasmids used in this study are provided as RAR archive in Genbank format.

CGGGGAGAGAGGATTTTGAGGAAATAATTAATTGAATTACTTGTATTATTGATAAAGTAATTTAGATAAGTTGTTAGTACAACTTATTGCAACTATGGATAGACAAAAATCACTTATTTTAAGGGGAGAAGTATATAACAACTTATTATAAAATTTCTGAGCAGCCGGCCTCCTCCAATCAATCATAGAGAGTGGAGCCCATTCTGGAAAACCAAGGAACCCCCAACTTGCACTGGTGCGGTGGCCCAATTCAAAAAAAACGGGGCCCAGCAAAGTAACGCCGTTGTAACGATTTATCAATCCAAACTCAAAAGGCGGCGAGATGCGTTTAACTCCGTGAAATTAACAAACCGCCAACAACTTGCAATTTGCAACTACCGTTTCCAAGAAGAACTCAACCACACAACGTATCCTATCCCAAACCACACGCAACTAGTGACGCGTCATAAGGACACGTGTCACAATTTGACTGGTTAATAATTTCACCGCTTTTGCTATAAATTACCTCCAATCCCCTTAGCTTCTTCACAATTCAGTTCCCAACCCTAACAATTCTTGTTCATATCGCTTCTCTCTACTTTCAAGGTATGATCCAATTTCTCTCTTCTTTCTCTGTAATCCTTTCGTTGAGTTTTGTTTCCGATCAATCATAGGTAGTTTTCTTGTTTCGAAGCATGAGATCTAGGAATTTTTTGTGATTTTCCAAAATTGAGATCGGTTTGAAATTGAATTTTACAGCTTGAATCTCAGATCTTGTTTTATCAATGTTTTCGATGGCTTGCGATGTAGATCTATGATAATTGTGGTTCAGTTTTGTTAGGAATCGATTTCGGTTTAGCAATTGCAGATTAATTAGGGTTTCCAATTGAATTCTTCAGATCCGTTATGGAATTATGTCAAATAATTTATTCAAATTGGAAATTATTGTTAGATCCACTCTTAATCTGTTTGATCCAAGCTTCAATTAGGGTTTTCACTTGTTTCAATTTCTTGTTATGGATTCTGATTTATCTGTTGATGTTAGATCCACTCTTAATCTGTTTGATCCAAGCGTTAATTAGGGTTTTCACTTGTTTCAATTTCGTGTGTTGGATTCTGATTTATCTGTTGTTGATGTGATTACAGaaattactgatgagtccgtgaggacgaaacgagtaagctcgtctAATTTCTACTAAGTGTAGATNNNNNNNNNNNNNNNNNNNNNNNggccggcatggtcccagcctcctcgctggcgccggctgggcaacatgcttcggcatggcgaatgggacTGACGCTAGCCTAGTTCGaaattactgatgagtccgtgaggacgaaacgagtaagctcgtctAATTTCTACTAAGTGTAGATNNNNNNNNNNNNNNNNNNNNNNNggccggcatggtcccagcctcctcgctggcgccggctgggcaacatgcttcggcatggcgaatgggacTGAGCAGGCCTCCCAG…

**Appendix S1 crRNA expression cassette *LjUbi_pro__RZ (cas12a).***

The sequence (5’ to 3’) of the crRNA expression cassette *LjUbi_pro__RZ* *(cas12a)* is shown with the following colour code: *LjUbi_pro_* - HH (hammerhead ribozyme) - *Lb*DR – Spacer 1 (I) / (II) - HDV (hepatitis delta virus ribozyme) - dummy - HH - *Lb*DR – Spacer 2 (I) / (II) - HDV - *PsRBCS-3A_term_*. LI fusion sites of the Golden Gate system (GG) are highlighted in yellow and underlined: GCGG = LI fusion site A, aaat = LI fusion site B^+^, AGAT = LI fusion site A^+^, ggca = LI fusion site C^+^, GTGT = LI fusion site C^-^, ggcc = LI fusion site D^+^, TGAG = LI fusion site F. Note that the fusion sites B^+^, A^+^, C^+^, C^-^, and D^+^ were added to the published Golden Gate (GG) system. LI fusion sites B to E of the GG system were therefore omitted. Sequences of B^+^, A^+^, C^+^, C^-^ and D^+^ are elementary parts of the crRNA expression cassette. Thereby, the crRNA expression cassette is not interrupted by artificial fusion sites of the GG system. This allows the cloning of oligonucleotide templates (Appendix S11 and S12) into the crRNA expression cassette and additionally the production of functional crRNAs. Used Spacer sequences can be found in Table S3. See table S1 for abbreviations.

GCGGGGAGAGAGGATTTTGAGGAAATAATTAATTGAATTACTTGTATTATTGATAAAGTAATTTAGATAAGTTGTTAGTACAACTTATTGCAACTATGGATAGACAAAAATCACTTATTTTAAGGGGAGAAGTATATAACAACTTATTATAAAATTTCTGAGCAGCCGGCCTCCTCCAATCAATCATAGAGAGTGGAGCCCATTCTGGAAAACCAAGGAACCCCCAACTTGCACTGGTGCGGTGGCCCAATTCAAAAAAAACGGGGCCCAGCAAAGTAACGCCGTTGTAACGATTTATCAATCCAAACTCAAAAGGCGGCGAGATGCGTTTAACTCCGTGAAATTAACAAACCGCCAACAACTTGCAATTTGCAACTACCGTTTCCAAGAAGAACTCAACCACACAACGTATCCTATCCCAAACCACACGCAACTAGTGACGCGTCATAAGGACACGTGTCACAATTTGACTGGTTAATAATTTCACCGCTTTTGCTATAAATTACCTCCAATCCCCTTAGCTTCTTCACAATTCAGTTCCCAACCCTAACAATTCTTGTTCATATCGCTTCTCTCTACTTTCAAGGTATGATCCAATTTCTCTCTTCTTTCTCTGTAATCCTTTCGTTGAGTTTTGTTTCCGATCAATCATAGGTAGTTTTCTTGTTTCGAAGCATGAGATCTAGGAATTTTTTGTGATTTTCCAAAATTGAGATCGGTTTGAAATTGAATTTTACAGCTTGAATCTCAGATCTTGTTTTATCAATGTTTTCGATGGCTTGCGATGTAGATCTATGATAATTGTGGTTCAGTTTTGTTAGGAATCGATTTCGGTTTAGCAATTGCAGATTAATTAGGGTTTCCAATTGAATTCTTCAGATCCGTTATGGAATTATGTCAAATAATTTATTCAAATTGGAAATTATTGTTAGATCCACTCTTAATCTGTTTGATCCAAGCTTCAATTAGGGTTTTCACTTGTTTCAATTTCTTGTTATGGATTCTGATTTATCTGTTGATGTTAGATCCACTCTTAATCTGTTTGATCCAAGCGTTAATTAGGGTTTTCACTTGTTTCAATTTCGTGTGTTGGATTCTGATTTATCTGTTGTTGATGTGATTACAGAATTTCTACTAAGTGTAGATNNNNNNNNNNNNNNNNNNNNNNNTTTTATTTTTTAATTTCTACTAAGTGTAGATNNNNNNNNNNNNNNNNNNNNNNNTTTTATTTTTTAATTTCTACTAAGTGTAGATTGAGCAGGCCTCCCAG…

**Appendix S2 crRNA expression cassette *LjUbi_pro__ T_4_AT_6_ (cas12a).***

The sequence (5’ to 3’) of the crRNA expression cassette *LjUbi_pro__T_4_AT_6_* *(cas12a)* is shown with the following colour code: *LjUbi_pro_* - *Lb*DR – Spacer 1 (I) / (II) - T_4_AT_6_ - *Lb*DR – Spacer 2 (I) / (II) - T_4_AT_6_ - *Lb*DR - *PsRBCS-3A_term_*. LI fusion sites of the Golden Gate system (GG) are highlighted in yellow and underlined: GCGG = LI fusion site A, AGAT = LI fusion site A^+^, TTTT = LI fusion site E^+^, TGAG = LI fusion site F. Note that the fusion sites A^+^ and E^+^ were added to the published Golden Gate (GG) system. LI fusion sites B to E of the GG system were therefore omitted. Sequences of A^+^ and E^+^ are elementary parts of the crRNA expression cassette. Thereby, the crRNA expression cassette is not interrupted by artificial fusion sites of the GG system. This allows the cloning of oligonucleotide templates (Appendix S13) into the crRNA expression cassette and additionally the production of functional crRNAs. Used Spacer sequences can be found in Table S3. See table S1 for abbreviations.

GCGGAATCTTTGAGAGGGAATAAAAGAAATACTTCCAAAAATCCATTAGAAGAACTATAAATCTTCTGGGCATGTAAATATAACGTAAAATATATGTTATGTTACAACCTTATTTCATTCACATTTTAATTTTTTTTCACACAATTTTTATTTAATTTTGTTTTTATCTCTATTTTATTTTCATATGATAATACATATTATCTCTTTATTTTACTTTATGTGTCTCTTTGCTCTCACATTTTATTTCTTAACTAACATATTCTCATTAGCAGTAGCTATCGTAAGACTAATCTCACACAATAATTAATTATCCATTCTATTTATGAATTTTTATCATTTAATAATTCTTTACCTGTGCAAATTTTGAAGGAAAAAGTTTCTGAATATAAAATAACAATAATACTAATTAGATGTCAAATCAAATCACCCAGGCTAGACAATAGTGGTAATGAAGAAGGCTAGAGGTAGCTCTGTGCCTCTAGGGGAGTGAGAGACGTTTTTCATTTATTGGTGTGTTGAAACTTGAAACGTTGATGTTCTCCACAGCTCCACGTATTCTTTCGTCCTAGAACGAACTAGAGCAACATAATCTGTCTCTTCCTGAAGCTTCATTGTTTTTCTTTCCTTCTAGCAGCATCTTCTTAACAGACGAAGAAGGGACTCAGATGCTTGCTTGCATGCTTCCAAAGTTTGGAGCTGAGGAGACTTGTGCTATTTGAGTCTGTTTTAGCATTCGCAAGCCCCACACCGGATAAACATACAGAAATCTGAGTGTTTATAAGCAAATAGTCAGCAACAAGGTTCGaaattactgatgagtccgtgaggacgaaacgagtaagctcgtctAATTTCTACTAAGTGTAGATNNNNNNNNNNNNNNNNNNNNNNNggccggcatggtcccagcctcctcgctggcgccggctgggcaacatgcttcggcatggcgaatgggacTGACGCTAGCCTAGTTCGaaattactgatgagtccgtgaggacgaaacgagtaagctcgtctAATTTCTACTAAGTGTAGATNNNNNNNNNNNNNNNNNNNNNNNggccggcatggtcccagcctcctcgctggcgccggctgggcaacatgcttcggcatggcgaatgggacTTTTTTGTCTGTC

**Appendix S3 crRNA expression cassette *LjU6_pro__RZ (cas12a).***

The sequence (5’ to 3’) of the crRNA expression cassette *Lj*U6_pro__RZ is shown with the following colour code: *LjU6-1_pro_* - G - HH (hammerhead ribozyme) - *Lb*DR – Spacer 1 (I) / (II) - HDV (hepatitis delta virus ribozyme) - dummy - HH (hammerhead ribozyme) - *Lb*DR – Spacer 2 (I) / (II) - Poly-T. LI fusion sites of the Golden Gate system (GG) are highlighted in yellow and underlined: GCGG = LI fusion site A, aaat = LI fusion site B^+^, AGAT = LI fusion site A^+^, ggca = LI fusion site C^+^, GTGT = LI fusion site C^-^, ggcc = LI fusion site D^+^, TGTC = LI fusion site G. Note: The fusion sites B^+^, A^+^, C^+^, C^-^, and D^+^ were added to the GG system. LI fusion sites B to F of the GG system were therefore omitted. Sequences of B^+^, A^+^, C^+^, C^-^ and D^+^ are elementary parts of the crRNA expression cassette. Thereby, the crRNA expression cassette is not interrupted by artificial fusion sites of the GG system. This allows the cloning of oligonucleotide templates (Appendix S11 and S12) into the crRNA expression cassette and additionally the production of functional crRNAs. A =The *Bsa*I site in *LjU6-1_pro_* was mutated (T → A) to fit to the GG system. C = The *Bbs*I (*Bpi*I) site in *LjU6-1_pro_* has been already mutated (G → C) (Wang et al., 2016). G = Additional G to facilitate pre-crRNA expression. Used Spacer sequences can be found in Table S3. See table S1 for abbreviations.

GCGGAATCTTTGAGAGGGAATAAAAGAAATACTTCCAAAAATCCATTAGAAGAACTATAAATCTTCTGGGCATGTAAATATAACGTAAAATATATGTTATGTTACAACCTTATTTCATTCACATTTTAATTTTTTTTCACACAATTTTTATTTAATTTTGTTTTTATCTCTATTTTATTTTCATATGATAATACATATTATCTCTTTATTTTACTTTATGTGTCTCTTTGCTCTCACATTTTATTTCTTAACTAACATATTCTCATTAGCAGTAGCTATCGTAAGACTAATCTCACACAATAATTAATTATCCATTCTATTTATGAATTTTTATCATTTAATAATTCTTTACCTGTGCAAATTTTGAAGGAAAAAGTTTCTGAATATAAAATAACAATAATACTAATTAGATGTCAAATCAAATCACCCAGGCTAGACAATAGTGGTAATGAAGAAGGCTAGAGGTAGCTCTGTGCCTCTAGGGGAGTGAGAGACGTTTTTCATTTATTGGTGTGTTGAAACTTGAAACGTTGATGTTCTCCACAGCTCCACGTATTCTTTCGTCCTAGAACGAACTAGAGCAACATAATCTGTCTCTTCCTGAAGCTTCATTGTTTTTCTTTCCTTCTAGCAGCATCTTCTTAACAGACGAAGAAGGGACTCAGATGCTTGCTTGCATGCTTCCAAAGTTTGGAGCTGAGGAGACTTGTGCTATTTGAGTCTGTTTTAGCATTCGCAAGCCCCACACCGGATAAACATACAGAAATCTGAGTGTTTATAAGCAAATAGTCAGCAACAAGGTTCGaaattactgatgagtccgtgaggacgaaacgagtaagctcgtctAATTTCTACTAAGTGTAGATNNNNNNNNNNNNNNNNNNNNNNNggccggcatggtcccagcctcctcgctggcgccggctgggcaacatgcttcggcatggcgaatgggacTTTTTTGACGCTAGCCTAGTTCGAATCTTTGAGAGGGAATAAAAGAAATACTTCCAAAAATCCATTAGAAGAACTATAAATCTTCTGGGCATGTAAATATAACGTAAAATATATGTTATGTTACAACCTTATTTCATTCACATTTTAATTTTTTTTCACACAATTTTTATTTAATTTTGTTTTTATCTCTATTTTATTTTCATATGATAATACATATTATCTCTTTATTTTACTTTATGTGTCTCTTTGCTCTCACATTTTATTTCTTAACTAACATATTCTCATTAGCAGTAGCTATCGTAAGACTAATCTCACACAATAATTAATTATCCATTCTATTTATGAATTTTTATCATTTAATAATTCTTTACCTGTGCAAATTTTGAAGGAAAAAGTTTCTGAATATAAAATAACAATAATACTAATTAGATGTCAAATCAAATCACCCAGGCTAGACAATAGTGGTAATGAAGAAGGCTAGAGGTAGCTCTGTGCCTCTAGGGGAGTGAGAGACGTTTTTCATTTATTGGTGTGTTGAAACTTGAAACGTTGATGTTCTCCACAGCTCCACGTATTCTTTCGTCCTAGAACGAACTAGAGCAACATAATCTGTCTCTTCCTGAAGCTTCATTGTTTTTCTTTCCTTCTAGCAGCATCTTCTTAACAGACGAAGAAGGGACTCAGATGCTTGCTTGCATGCTTCCAAAGTTTGGAGCTGAGGAGACTTGTGCTATTTGAGTCTGTTTTAGCATTCGCAAGCCCCACACCGGATAAACATACAGAAATCTGAGTGTTTATAAGCAAATAGTCAGCAACAAGGTTCGaaattactgatgagtccgtgaggacgaaacgagtaagctcgtctAATTTCTACTAAGTGTAGATNNNNNNNNNNNNNNNNNNNNNNNggccggcatggtcccagcctcctcgctggcgccggctgggcaacatgcttcggcatggcgaatgggacTTTTTTGTC

**Appendix S4 crRNA expression cassette 2x *LjU6_pro__2xRZ (cas12a).***

The sequence (5’ to 3’) of the crRNA expression cassette 2x *LjU6_pro__2xRZ (cas12a)* is shown with the following colour code: *LjU6-1_pro_* - G - HH (hammerhead ribozyme) - *Lb*DR – Spacer 1 (I) / (II) - HDV (hepatitis delta virus ribozyme) - Poly-T – dummy - *LjU6-1_pro_* - G - HH - *Lb*DR – Spacer 2 (I) / (II) - HDV - Poly-T. LI fusion sites of the Golden Gate system (GG) are highlighted in yellow and underlined: GCGG = LI fusion site A, aaat = LI fusion site B^+^, AGAT = LI fusion site A^+^, ggca = LI fusion site C^+^, tact = LI fusion site D^-^, GTGT = LI fusion site C^-^, ggcc = LI fusion site D^+^, TGTC = LI fusion site G. Note that the fusion sites B^+^, A^+^, C^+^, C^-^, D^+^ and D^-^ were added to the published Golden Gate (GG) system. LI fusion sites B to F of the GG system were therefore omitted. Sequences of B^+^, A^+^, C^+^, C^-^, D^+^ and D^-^ are elementary parts of the crRNA expression cassette. Thereby, the crRNA expression cassette is not interrupted by artificial fusion sites of the GG system. This allows the cloning of oligonucleotide templates (Appendix S11 and S12) into the crRNA expression cassette and additionally the production of functional crRNAs. A =The *Bsa*I site in *LjU6-1_pro_* was mutated (T → A) to fit to the GG system. C = The *Bbs*I (*Bpi*I) site in *LjU6-1_pro_* has been already mutated (G → C) (Wang et al., 2016). G = Additional G to facilitate pre-crRNA expression. Used Spacer sequences can be found in Table S3. See table S1 for abbreviations.

GCGGATGCCTATCTTATAtgatcaatgaggcatttaattgggtgcatatgatggtgaaaaaaggtgcagctcctggcttgggaatgatgactcatgtggaatttggtcttaaatttatcacatccttttgggatgtgatgattgtatcacttgttcattttgcaaagacaaggtgcactgctacaaactttggtttaatctgaaataaaacaaaactcactgagaggaagatgcatcccagtaggtgaaagtcgagaaggatttgcatgttactattacacttgctttttagtcccacatcgtctgaaacataaaatatttcagcgtttaaatacttcaaGCGAACCAGTAGGCTTGAATTTCTACTAAGTGTAGATNNNNNNNNNNNNNNNNNNNNNNNAATTTCTACTAAGTGTAGATNNNNNNNNNNNNNNNNNNNNNNNAATTTCTACTAAGTGTAGATTTTTTTTGGCAAAAATTTTCAGATTTTTTCTTCATCTGTAGATTTCTGGGTTTTTTTTTCCGTTTCGTGAATCATAAGTGAAGTTTTGGATGCAAATCTGCGCGAAAAAAGTTGGACCTGCAATGAGCTTATTTAGATAGCTAAGACAAAGTGATTGGTCCGTTGTTTCAGTTCTGATTGTCAGAGAGTTTGTTTCGAGTCGGCGACACCAATGCGTTTTGTTAACCAGATTTCGGGTAAGAAATGTATCGAGAGTTTGTTTCCAGACGGCTACATCATTTTCTTATGAAGGGTGAAATTAGATAGACCAAAGATTGAAACACAACATTTCTTTCACAAAAATATAATAAACTTGATAGCATTTAGGATCAGCTGAG

**Appendix S5 crRNA expression cassette *MtU6_pro__SP (cas12a).***

The sequence (5’ to 3’) of the crRNA expression cassette *LjU6_pro__SP (cas12a)* is shown with the following colour code: *MtU6_pro_* - G - *Lb*DR – Spacer 1 (I) - *Lb*DR – Spacer 2 (I) - *Lb*DR - *U6-1_term_* (with Poly-T). LI fusion sites of the Golden Gate system (GG) are highlighted in yellow and underlined: GCGG = LI fusion site A, TTTC = LI fusion site H^+^, AGAT = LI fusion site A^+^, AATT = LI fusion site A^-^, TGAG = LI fusion site F. Note that the fusion sites H^+^, A^+^ and A^-^ were added to the published GG system. LI fusion sites B to E of the GG system were therefore omitted. Sequences of H^+^, A^+^ and A^-^ are elementary parts of the crRNA expression cassette. Thereby, the crRNA expression cassette is not interrupted by artificial fusion sites of the GG system. This allows the cloning of oligonucleotide templates (Appendix S10) into the crRNA expression cassette and additionally the production of functional crRNAs. G = Additional G to facilitate pre-crRNA expression. Used Spacer sequences can be found in Table S3. See table S1 for abbreviations.

GCGGTGATCAAAAGTCCCacatcgatcaggtgatatatagcagcttagtttatataatgataGAGTCGACATAGCGATTGAATTTCTACTAAGTGTAGATNNNNNNNNNNNNNNNNNNNNNNNAATTTCTACTAAGTGTAGATNNNNNNNNNNNNNNNNNNNNNNNAATTTCTACTAAGTGTAGATTTTTTTTGGCAAAAATTTTCAGATTTTTTCTTCATCTGTAGATTTCTGGGTTTTTTTTTCCGTTTCGTGAATCATAAGTGAAGTTTTGGATGCAAATCTGCGCGAAAAAAGTTGGACCTGCAATGAGCTTATTTAGATAGCTAAGACAAAGTGATTGGTCCGTTGTTTCAGTTCTGATTGTCAGAGAGTTTGTTTCGAGTCGGCGACACCAATGCGTTTTGTTAACCAGATTTCGGGTAAGAAATGTATCGAGAGTTTGTTTCCAGACGGCTACATCATTTTCTTATGAAGGGTGAAATTAGATAGACCAAAGATTGAAACACAACATTTCTTTCACAAAAATATAATAAACTTGATAGCATTTAGGATCAGCTGAG

**Appendix S6 crRNA expression cassette *AtU6MoClo_pro__SP (cas12a).***

The sequence (5’ to 3’) of the crRNA expression cassette *LjU6_pro__SP (cas12a)* is shown with the following colour code: *AtU6MoClo_pro_* - G - *Lb*DR – Spacer 1 (I) - *Lb*DR – Spacer 2 (I) - *Lb*DR - *U6-1_term_* (with Poly-T). LI fusion sites of the Golden Gate system (GG) are highlighted in yellow and underlined: GCGG = LI fusion site A, TTTC = LI fusion site H^+^, AGAT = LI fusion site A^+^, AATT = LI fusion site A^-^, TGAG = LI fusion site F. Note that the fusion sites H^+^, A^+^ and A^-^ were added to the published GG system. LI fusion sites B to E of the GG system were therefore omitted. Sequences of H^+^, A^+^ and A^-^ are elementary parts of the crRNA expression cassette. Thereby, the crRNA expression cassette is not interrupted by artificial fusion sites of the GG system. This allows the cloning of oligonucleotide templates (Appendix S10) into the crRNA expression cassette and additionally the production of functional crRNAs. G = Additional G to facilitate pre-crRNA expression. Used Spacer sequences can be found in Table S3. See table S1 for abbreviations.

GCGGAGAAATCTCAAAATTCCGGCAGAACAATTTTGAATCTCGATCCGTAGAAACCAGACGGTCATTGTTTTAGTTCCACCACGATTATATTTGAAATTTACGTGAGTGTGAGTGAGACTTGCATAAGAAAATAAAATCTTTAGTTGGGAAAAAATTCAATAATATAAATGGGCTTGAGAAGGAAGCGAGGGATAGGCCTTTTTCTAAAATAGGCCCATTTAAGCTATTAACAATCTTCAAAAGTACCACAGCGCTTAGGTAAAGAAAGCAGCTGAGTTTATATATggttaGACACGAAGTAGTGATTGAATTTCTACTAAGTGTAGATNNNNNNNNNNNNNNNNNNNNNNNAATTTCTACTAAGTGTAGATNNNNNNNNNNNNNNNNNNNNNNNAATTTCTACTAAGTGTAGATTTTTTTTGGCAAAAATTTTCAGATTTTTTCTTCATCTGTAGATTTCTGGGTTTTTTTTTCCGTTTCGTGAATCATAAGTGAAGTTTTGGATGCAAATCTGCGCGAAAAAAGTTGGACCTGCAATGAGCTTATTTAGATAGCTAAGACAAAGTGATTGGTCCGTTGTTTCAGTTCTGATTGTCAGAGAGTTTGTTTCGAGTCGGCGACACCAATGCGTTTTGTTAACCAGATTTCGGGTAAGAAATGTATCGAGAGTTTGTTTCCAGACGGCTACATCATTTTCTTATGAAGGGTGAAATTAGATAGACCAAAGATTGAAACACAACATTTCTTTCACAAAAATATAATAAACTTGATAGCATTTAGGATCAGCTGAG

**Appendix S7 crRNA expression cassette *AtU6-1_pro__SP (cas12a).***

The sequence (5’ to 3’) of the crRNA expression cassette *LjU6_pro__SP (cas12a)* is shown with the following colour code: *MtU6-1_pro_* - G - *Lb*DR – Spacer 1 (I) - *Lb*DR – Spacer 2 (I) - *Lb*DR - *U6-1_term_* (with Poly-T). LI fusion sites of the Golden Gate system (GG) are highlighted in yellow and underlined: GCGG = LI fusion site A, TTTC = LI fusion site H^+^, AGAT = LI fusion site A^+^, AATT = LI fusion site A^-^, TGAG = LI fusion site F. Note that the fusion sites H^+^, A^+^ and A^-^ were added to the published GG system. LI fusion sites B to E of the GG system were therefore omitted. Sequences of H^+^, A^+^ and A^-^ are elementary parts of the crRNA expression cassette. Thereby, the crRNA expression cassette is not interrupted by artificial fusion sites of the GG system. This allows the cloning of oligonucleotide templates (Appendix S10) into the crRNA expression cassette and additionally the production of functional crRNAs. G = Additional G to facilitate pre-crRNA expression. Used Spacer sequences can be found in Table S3. See table S1 for abbreviations.

GCGGCTTTTTTTCTTCTTCTTCGTTCATACAGTTTTTTTTTGTTTATCAGCTTACATTTTCTTGAACCGTAGCTTTCGTTTTCTTCTTTTTAACTTTCCATTCGGAGTTTTTGTATCTTGTTTCATAGTTTGTCCCAGGATTAGAATGATTAGGCATCGAACCTTCAAGAATTTGATTGAATAAAACATCTTCATTCTTAAGATATGAAGATAATCTTCAAAAGGCCCCTGGGAATCTGAAAGAAGAGAAGCAGGCCCATTTATATGGGAAAGAACAATAGTATTTCTTATATAGGCCCATTTAAGTTGAAAACAATCTTCAAAAGTCCCACATCGCTTAGATAAGAAAACGAAGCTGAGTTTATATACAGCTAGAGTCGAAGTAGTGATTGAATTTCTACTAAGTGTAGATNNNNNNNNNNNNNNNNNNNNNNNAATTTCTACTAAGTGTAGATNNNNNNNNNNNNNNNNNNNNNNNAATTTCTACTAAGTGTAGATTTTTTTTGGCAAAAATTTTCAGATTTTTTCTTCATCTGTAGATTTCTGGGTTTTTTTTTCCGTTTCGTGAATCATAAGTGAAGTTTTGGATGCAAATCTGCGCGAAAAAAGTTGGACCTGCAATGAGCTTATTTAGATAGCTAAGACAAAGTGATTGGTCCGTTGTTTCAGTTCTGATTGTCAGAGAGTTTGTTTCGAGTCGGCGACACCAATGCGTTTTGTTAACCAGATTTCGGGTAAGAAATGTATCGAGAGTTTGTTTCCAGACGGCTACATCATTTTCTTATGAAGGGTGAAATTAGATAGACCAAAGATTGAAACACAACATTTCTTTCACAAAAATATAATAAACTTGATAGCATTTAGGATCAGCTGAG

**Appendix S8 crRNA expression cassette *AtU6-26_pro__SP (cas12a).***

The sequence (5’ to 3’) of the crRNA expression cassette *LjU6_pro__SP (cas12a)* is shown with the following colour code: *AtU6-26_pro_* - G - *Lb*DR – Spacer 1 (I) - *Lb*DR – Spacer 2 (I) - *Lb*DR - *U6-1_term_* (with Poly-T). LI fusion sites of the Golden Gate system (GG) are highlighted in yellow and underlined: GCGG = LI fusion site A, TTTC = LI fusion site H^+^, AGAT = LI fusion site A^+^, AATT = LI fusion site A^-^, TGAG = LI fusion site F. Note that the fusion sites H^+^, A^+^ and A^-^ were added to the published GG system. LI fusion sites B to E of the GG system were therefore omitted. Sequences of H^+^, A^+^ and A^-^ are elementary parts of the crRNA expression cassette. Thereby, the crRNA expression cassette is not interrupted by artificial fusion sites of the GG system. This allows the cloning of oligonucleotide templates (Appendix S10) into the crRNA expression cassette and additionally the production of functional crRNAs. G = Additional G to facilitate pre-crRNA expression. Used Spacer sequences can be found in Table S3. See table S1 for abbreviations.

GCGGCTTTTTTTCTTCTTCTTCGTTCATACAGTTTTTTTTTGTTTATCAGCTTACATTTTCTTGAACCGTAGCTTTCGTTTTCTTCTTTTTAACTTTCCATTCGGAGTTTTTGTATCTTGTTTCATAGTTTGTCCCAGGATTAGAATGATTAGGCATCGAACCTTCAAGAATTTGATTGAATAAAACATCTTCATTCTTAAGATATGAAGATAATCTTCAAAAGGCCCCTGGGAATCTGAAAGAAGAGAAGCAGGCCCATTTATATGGGAAAGAACAATAGTATTTCTTATATAGGCCCATTTAAGTTGAAAACAATCTTCAAAAGTCCCACATCGCTTAGATAAGAAAACGAAGCTGAGTTTATATACAGCTAGAGTCGAAGTAGTGATTGNNNNNNNNNNNNNNNNNNNNgttttagagctagaaatagcaagttaaaataaggctagtccgttatcaacttgaaaaagtggcaccgagtcggtgcTTTTTTTGGCAAAAATTTTCAGATTTTTTCTTCATCTGTAGATTTCTGGGTTTTTTTTTCCGTTTCGTGAATCATAAGTGAAGTTTTGGATGCAAATCTGCGCGAAAAAAGTTGGACCTGCAATGAGCTTATTTAGATAGCTAAGACAAAGTGATTGGTCCGTTGTTTCAGTTCTGATTGTCAGAGAGTTTGTTTCGAGTCGGCGACACCAATGCGTTTTGTTAACCAGATTTCGGGTAAGAAATGTATCGAGAGTTTGTTTCCAGACGGCTACATCATTTTCTTATGAAGGGTGAAATTAGATAGACCAAAGATTGAAACACAACATTTCTTTCACAAAAATATAATAAACTTGATAGCATTTAGGATCAGCcacctgacctaagctCTTTTTTTCTTCTTCTTCGTTCATACAGTTTTTTTTTGTTTATCAGCTTACATTTTCTTGAACCGTAGCTTTCGTTTTCTTCTTTTTAACTTTCCATTCGGAGTTTTTGTATCTTGTTTCATAGTTTGTCCCAGGATTAGAATGATTAGGCATCGAACCTTCAAGAATTTGATTGAATAAAACATCTTCATTCTTAAGATATGAAGATAATCTTCAAAAGGCCCCTGGGAATCTGAAAGAAGAGAAGCAGGCCCATTTATATGGGAAAGAACAATAGTATTTCTTATATAGGCCCATTTAAGTTGAAAACAATCTTCAAAAGTCCCACATCGCTTAGATAAGAAAACGAAGCTGAGTTTATATACAGCTAGAGTCGAAGTAGTGATTGNNNNNNNNNNNNNNNNNNNNgttttagagctagaaatagcaagttaaaataaggctagtccgttatcaacttgaaaaagtggcaccgagtcggtgcTTTTTTTGGCAAAAATTTTCAGATTTTTTCTTCATCTGTAGATTTCTGGGTTTTTTTTTCCGTTTCGTGAATCATAAGTGAAGTTTTGGATGCAAATCTGCGCGAAAAAAGTTGGACCTGCAATGAGCTTATTTAGATAGCTAAGACAAAGTGATTGGTCCGTTGTTTCAGTTCTGATTGTCAGAGAGTTTGTTTCGAGTCGGCGACACCAATGCGTTTTGTTAACCAGATTTCGGGTAAGAAATGTATCGAGAGTTTGTTTCCAGACGGCTACATCATTTTCTTATGAAGGGTGAAATTAGATAGACCAAAGATTGAAACACAACATTTCTTTCACAAAAATATAATAAACTTGATAGCATTTAGGATCAGCtgtc

**Appendix S9 sgRNA expression cassette 2x *AtU6-26_pro_ (cas9).***

The sequence (5’ to 3’) of the sgRNA expression cassette 2x *AtU6-26_pro_ (cas9)* is shown with the following colour code: *AtU6-26_pro_* - G - Spacer 1 (I) / (II) - tracr - *U6-1_term_* (with Poly-T) - dummy - *AtU6-26_pro_* - G - Spacer 2 (I) / (II) - tracr - *U6-1_term_* (with Poly-T). LI fusion sites of the Golden Gate system (GG) are highlighted in yellow and underlined: GCGG = LI fusion site A, ATTG = LI fusion site I^+^, taga = LI fusion site F^+^, agct = LI fusion site F^-^, AGTG = LI fusion site I^-^, gttt = LI fusion site G^-^, tgtc = LI fusion site G. Note that the fusion sites I^+^, F^+^, F^-^, I^-^ and G^-^ were added to the published GG system. LI fusion sites B to F of the GG system were therefore omitted. Sequences of I^+^, F^+^, F^-^, I^-^ and G^-^ are elementary parts of the sgRNA expression cassette. Thereby, the sgRNA expression cassette is not interrupted by artificial fusion sites of the GG system. This allows the cloning of oligonucleotide templates (Appendix S14 and S15) into the sgRNA expression cassette and additionally the production of functional sgRNAs. G = Additional G to facilitate sgRNA expression; can be omitted if the subsequent spacer sequence starts with a G. Used Spacer sequences can be found in Table S3. See table S1 for abbreviations.

ATGAAGACTTTACGGGTCTCAAGATNNNNNNNNNNNNNNNNNNNNAATTTCTACTAAGTGTAGATNNNNNNNNNNNNNNNNNNNNAATTTGAGACCTCTGAAGTCTTCAT

**Appendix S10** **Oligonucleotide with Spacer 1 and Spacer 2 templates for cloning into the crRNA expression cassettes *LjU6_pro__SP (cas12a), MtU6_pro__SP (cas12a), AtU6MoClo_pro__SP (cas12a), AtU6-1_pro__SP (cas12a) and AtU6-26_pro__SP (cas12a)*.**

The template (5‘ to 3‘) is shown with the following colour code: *Bpi*I restriction site - *Bsa*I restriction site - *Lb*DR (part) – Spacer 1 (I) / (II) - *Lb*DR – Spacer 2 (I) / (II) - *Lb*DR (part) - *Bsa*I restriction site - *Bpi*I restriction site.

LI fusion sites of the Golden Gate system (GG) are highlighted in yellow and underlined: TACG and TCTG = fusion sites for directional LI cloning in the backbone BB3, AGAT = L1 fusion sites A^+^ and AATT = L1 fusion site A^-^ for LII cloning. Note that the spacer sequences have to be added in the correct orientation, 5’ to 3’. Used Spacer sequences can be found in Table S3. See table S1 for abbreviations.

ATGAAGACTTTACGGGTCTCAAGATNNNNNNNNNNNNNNNNNNNNggccggcaTGAGACCTCTGAAGTCTTCAT

**Appendix S11 Oligonucleotide with Spacer 1 template for cloning into the crRNA expression cassettes *LjUbi_pro__RZ* *(cas12a)*, *LjU6_pro__RZ (cas12a)*, 2x *LjU6_pro__2xRZ (cas12a)* and 2x *AtU6-26_pro__2xRZ (cas12a)*.**

The oligonucleotide template (5‘ to 3‘) is shown with the following colour code: *Bpi*I restriction site - *Bsa*I restriction site - *Lb*DR (part) – Spacer 1 (I) / (II) - hepatitis delta virus ribozyme (HDV) - *BsaI* restriction site - *Bpi*I restriction site. LI fusion sites of the Golden Gate system (GG) are highlighted in yellow and underlined: TACG and TCTG = fusion sites for directional LI cloning in the backbone BB3, AGAT = L1 fusion sites A^+^ and ggca = LI fusion site C^+^ for LII cloning. Note that the Spacer 1 sequence has to be added in the correct orientation, 5’ to 3’. Used Spacer sequences can be found in Table S3. See table S1 for abbreviations.

ATGAAGACTTTACGGGTCTCAGTGTAGATNNNNNNNNNNNNNNNNNNNNggccTGAGACCTCTGAAGTCTTCAT

**Appendix S12 Oligonucleotide with Spacer 2 template for cloning into the crRNA expression cassettes *LjUbi_pro__RZ* *(cas12a)*, *LjU6_pro__RZ (cas12a)*, 2x *LjU6_pro__2xRZ (cas12a)* and 2x *AtU6-26_pro__2xRZ (cas12a)*.**

The oligonucleotide template (5‘ to 3‘) is shown with the following colour code: *Bpi*I restriction site - *Bsa*I restriction site - *Lb*DR (part) – Spacer 2 (I) / (II) - hepatitis delta virus ribozyme (HDV) - *Bsa*I restriction site - *Bpi*I restriction site. LI fusion sites of the Golden Gate system (GG) are highlighted in yellow and underlined: TACG and TCTG = fusion sites for directional LI cloning in the backbone BB3, GTGT = L1 fusion sites C^-^ and ggcc = LI fusion site D^+^ for LII cloning. Note that the Spacer 2 sequence has to be added in the correct orientation, 5’ to 3’. Used Spacer sequences can be found in Table S3. See table S1 for abbreviations.

ATGAAGACTTTACGGGTCTCAAGATNNNNNNNNNNNNNNNNNNNNTTTTATTTTTTAATTTCTACTAAGTGTAGATNNNNNNNNNNNNNNNNNNNNTTTTTGAGACCTCTGAAGTCTTCA

**Appendix S13 Oligonucleotide with Spacer 1 and Spacer 2 templates for cloning into the crRNA expression cassette *LjUbi_pro__T_4_AT_6_ (cas12a).***

The template (5‘ to 3‘) is shown with the following colour code: *Bpi*I restriction site - *Bsa*I restriction site - *Lb*DR (part) – Spacer 1 (I) / (II) - T_4_AT_6_ - *Lb*DR – Spacer 2 (I) / (II) - T_4_AT_6_ - *Bsa*I restriction site - *Bpi*I restriction site.

LI fusion sites of the Golden Gate system (GG) are highlighted in yellow and underlined: TACG and TCTG = fusion sites for directional LI cloning in the backbone BB3, AGAT = L1 fusion sites A^+^ and TTTT = LI fusion site E^+^ for LII cloning. Note that the Spacer 1 sequence has to be added in the correct orientation, 5’ to 3’. Used Spacer sequences can be found in Table S3. See table S1 for abbreviations.

ATGAAGACTTTACGGGTCTCAATTGNNNNNNNNNNNNNNNNNNNNgttttagaTGAGACCTCTGAAGTCTTCAT

**Appendix S14 Oligonucleotide with Spacer 1 template for cloning into the sgRNA expression cassette 2x *AtU6-26_pro_ (cas9)*.**

The oligonucleotide template (5‘ to 3‘) is shown with the following colour code: *Bpi*I restriction site - *Bsa*I restriction site - *AtU6-26_pro_* - G - Spacer 1 - tracr - *Bsa*I restriction site - *Bpi*I restriction site. LI fusion sites of the Golden Gate system (GG) are highlighted in yellow and underlined: TACG and TCTG = fusion sites for directional LI cloning in the backbone BB3, ATTG = LI fusion site I^+^ and taga = LI fusion site F^+^ for LII cloning. G = Additional G to facilitate sgRNA expression; can be omitted if the subsequent spacer sequence starts with a G. Note that the Spacer 1 sequence has to be added in the correct orientation, 5’ to 3’. Used Spacer sequence can be found in Table S3. See table S1 for abbreviations.

ATGAAGACTTTACGGGTCTCAAGTGATTGNNNNNNNNNNNNNNNNNNNNgtttTGAGACCTCTGAAGTCTTCAT

**Appendix S15 Oligonucleotide with Spacer 2 template for cloning into the sgRNA expression cassette 2x *AtU6-26_pro_ (cas9****)*.

The oligonucleotide template (5‘ to 3‘) is shown with the following colour code: *Bpi*I restriction site - *Bsa*I restriction site - *LjU6-1_pro_* - G - Spacer 2 - tracr - *Bsa*I restriction site - *Bpi*I restriction site. LI fusion sites of the Golden Gate system (GG) are highlighted in yellow and underlined: TACG and TCTG = fusion sites for directional LI cloning in the backbone BB3, AGTG = LI fusion site I^-^ and gttt = LI fusion site G^-^ for LII cloning. G = Additional G to facilitate sgRNA expression; can be omitted if the subsequent spacer sequence starts with a G. Note that the Spacer 2 sequence has to be added in the correct orientation, 5’ to 3’. Used Spacer sequence can be found in Table S3. See table S1 for abbreviations.

CACCATGAGCAAGTTAGAGAAATTCACCAATTGCTATTCCCTCAGTAAGACACTCAGATTCAAAGCCATCCCTGTAGGTAAGACACAAGAAAACATTGACAACAAGCGCCTTCTGGTAGAGGACGAGAAGAGAGCCGAAGATTACAAGGGCGTTAAAAAGTTGCTAGATAGGTACTACTTGTCTTTCATCAATGATGTACTGCATTCAATTAAGCTCAAGAACCTTAATAACTACATATCCTTGTTCCGCAAAAAGACCAGAACTGAGAAAGAGAATAAGGAGCTCGAAAACCTGGAGATCAATCTCAGAAAGGAGATTGCTAAAGCGTTTAAGGGGAATGAAGGATATAAGTCCCTCTTTAAGAAAGACATAATTGAGACAATCTTGCCTGAGTTTCTAGATGATAAGGACGAAATTGCCTTGGTGAATAGCTTTAACGGGTTCACCACAGCATTTACAGGGTTTTTTGATAATAGGGAGAACATGTTTAGTGAAGAGGCTAAATCAACATCTATTGCGTTTAGATGCATCAATGAAAATCTTACACGTTATATTAGTAACATGGATATATTTGAGAAGGTTGATGCAATATTCGATAAACACGAAGTTCAAGAGATTAAGGAGAAGATCTTGAATAGCGATTATGATGTTGAGGATTTCTTTGAAGGGGAGTTTTTCAATTTTGTTTTAACTCAGGAGGGCATTGATGTGTATAACGCTATTATTGGTGGTTTCGTGACTGAATCCGGAGAGAAGATTAAGGGACTTAATGAATACATTAACCTCTATAATCAGAAAACCAAGCAGAAGTTGCCGAAGTTTAAACCGCTTTACAAACAGGTCCTCAGTGATCGCGAATCTTTGAGCTTCTATGGTGAGGGATATACTAGTGATGAAGAGGTTTTGGAGGTGTTCCGAAATACCTTGAATAAGAATTCAGAGATTTTTAGTTCAATTAAGAAGTTGGAGAAACTGTTTAAGAACTTCGACGAATATTCATCTGCTGGCATTTTCGTCAAGAATGGCCCTGCGATAAGCACGATCAGCAAAGATATTTTTGGAGAGTGGAATGTGATTCGCGATAAATGGAATGCAGAATATGATGACATTCACTTGAAAAAGAAAGCAGTTGTGACAGAGAAGTATGAGGATGATAGGAGGAAAAGCTTCAAGAAAATTGGATCTTTCAGCTTAGAACAATTACAGGAATATGCAGATGCTGATCTTTCAGTTGTCGAAAAGCTCAAGGAAATCATCATCCAAAAGGTTGACGAGATTTACAAAGTCTATGGGAGTTCCGAAAAACTCTTCGATGCTGATTTTGTTCTTGAAAAGTCTCTGAAGAAAAACGATGCTGTTGTTGCTATCATGAAGGACTTACTTGATTCTGTGAAATCCTTCGAGAATTATATAAAAGCATTTTTCGGTGAGGGTAAAGAAACCAATCGTGACGAGTCATTTTATGGAGATTTTGTGCTGGCATATGACATTCTTCTAAAAGTGGATCACATCTATGATGCTATTAGGAACTATGTGACTCAGAAGCCGTATTCTAAAGACAAGTTCAAGTTGTATTTCCAAAACCCTCAGTTTATGGGTGGTTGGGATAAAGACAAAGAAACTGATTACAGAGCTACTATTTTAAGGTATGGCTCAAAATATTACTTGGCGATTATGGACAAAAAGTACGCAAAATGCCTACAAAAGATAGATAAGGATGATGTCAACGGAAACTATGAGAAAATCAACTACAAGCTTCTTCCTGGACCTAATAAGATGCTACCTAAGGTATTTTTCTCCAAGAAGTGGATGGCCTACTACAACCCATCTGAGGATATTCAGAAAATCTACAAGAACGGTACTTTCAAAAAAGGCGATATGTTCAATCTGAATGATTGTCATAAGTTGATCGATTTCTTTAAGGACTCAATATCAAGGTATCCTAAGTGGTCCAACGCATACGACTTTAACTTCTCTGAAACCGAGAAATACAAGGACATAGCAGGCTTCTATAGGGAAGTAGAAGAACAGGGATATAAAGTCTCCTTTGAATCAGCCTCAAAGAAGGAAGTGGATAAACTCGTTGAAGAAGGCAAATTGTATATGTTTCAGATCTACAACAAGGACTTTAGTGATAAGTCTCATGGGACCCCAAATCTTCATACGATGTACTTCAAGCTTCTTTTCGACGAGAACAATCACGGTCAAATTAGATTGTCTGGCGGGGCAGAACTCTTTATGAGAAGAGCTTCTCTGAAAAAAGAAGAGCTTGTTGTCCATCCAGCTAATTCTCCAATAGCCAACAAAAATCCCGATAACCCAAAAAAGACCACAACCCTCTCTTACGATGTATACAAGGACAAGAGGTTTTCCGAAGATCAATATGAGCTGCATATCCCCATAGCTATCAACAAATGCCCAAAGAATATCTTCAAGATCAACACTGAGGTTCGAGTCCTCTTAAAGCACGATGATAATCCCTACGTTATAGGAATTGACAGAGGTGAGAGAAACCTGCTTTATATTGTAGTGGTGGATGGTAAAGGGAATATTGTCGAGCAATATAGTCTGAATGAGATTATCAATAACTTCAACGGTATCAGAATAAAAACCGATTACCATAGCCTTCTAGATAAAAAAGAAAAGGAAAGGTTTGAAGCACGTCAGAATTGGACCTCTATTGAGAATATCAAAGAGCTAAAGGCGGGATATATCTCACAAGTTGTGCACAAAATATGTGAACTGGTGGAAAAGTATGATGCCGTGATAGCTTTGGAGGACTTGAACTCTGGATTTAAAAACTCCAGAGTAAAAGTCGAAAAACAAGTGTATCAGAAGTTCGAAAAGATGTTAATAGATAAGCTTAATTACATGGTTGATAAGAAATCCAATCCTTGTGCTACAGGTGGAGCTCTGAAAGGGTATCAGATCACTAACAAATTCGAGAGCTTCAAGTCTATGTCCACTCAAAACGGTTTCATCTTTTATATTCCTGCCTGGTTAACTAGTAAGATAGATCCATCTACTGGATTTGTTAATCTCCTTAAGACAAAATACACGAGCATTGCTGACTCTAAAAAGTTCATTTCAAGCTTTGACCGTATTATGTACGTTCCAGAAGAGGATCTTTTCGAATTCGCTTTGGACTATAAGAATTTCTCTAGGACTGATGCAGATTATATAAAGAAATGGAAGCTCTACTCATATGGGAACAGAATTAGGATCTTTAGAAACCCAAAGAAAAATAACGTTTTTGATTGGGAGGAGGTTTGTCTAACGTCAGCTTACAAGGAGTTGTTTAACAAGTACGGTATAAATTACCAACAAGGAGACATTCGAGCTCTGCTTTGTGAACAATCAGATAAAGCCTTTTACTCAAGTTTTATGGCACTTATGTCTCTTATGTTACAAATGCGTAACTCAATAACAGGAAGGACAGACGTTGATTTTCTAATTTCTCCCGTGAAGAACTCTGATGGGATTTTTTACGACAGTCGCAATTACGAGGCACAAGAAAATGCGATTTTGCCTAAGAATGCAGACGCAAATGGTGCTTACAATATCGCTAGAAAAGTCTTGTGGGCTATAGGCCAGTTCAAGAAAGCCGAAGATGAAAAGCTTGACAAAGTGAAGATAGCCATTAGTAATAAGGAATGGTTGGAATATGCACAGACTTCTGTTAAACATAAGG

**Appendix S16 Coding sequence of *cas12a^Lj^.***

Nucleotide sequence (5‘ to 3‘) of *cas12a^Lj^*. The *cas12a* CDS (coding sequence) is codon adapted for *Lotus japonicus* (*Lj*). First and last codon are shown in red (Start codon ATG, last codon CAT), note that a stop codon is missing in order to fuse a nuclear localization sequence (NLS) on 3’ end, CACC = LI fusion site A, AAGG = LI fusion site D, ATCG = LI fusion site (part of the *cas12a^Lj^* sequence) to combine the ordered two *cas12a^Lj^* strands, *cas12a^Lj^* p1 *and cas12a^Lj^* p2. See table S1 for abbreviations.

CACCATGAGCAAGCTCGAGAAGTTTACCAACTGCTACAGCCTCTCTAAGACCCTCAGGTTCAAGGCTATCCCTGTGGGAAAGACCCAAGAGAATATCGACAACAAGAGGCTCCTCGTCGAGGATGAGAAGAGAGCTGAAGATTACAAGGGCGTGAAGAAGCTCCTCGACAGGTAAGGACTTCTCATGAATATTAGTGGCAGATTAGTGTTGTTAAAGTCTTTGGTTAGATAATCGATGCCTCCTAATTGTCCATGTTTTACTGGTTTTCTACAATTACAGGTACTACCTCAGCTTCATCAACGATGTGCTCCACAGCATCAAGCTCAAGAACCTCAACAACTACATCAGCCTCTTCCGTAAGAAAACCAGGACCGAGAAAGAGAACAAAGAGCTTGAGAACCTCGAGATCAACCTCCGTAAAGAGATCGCCAAGGCTTTCAAGGGAAACGAGGGATACAAGAGCCTCTTCAAGAAGGATATTATCGAGACAATCCTGCCTGAGTTCCTGGACGATAAGGATGAGATCGCTCTCGTGAACAGCTTCAACGGATTCACTACTGCCTTCACCGGATTCTTCAGAAACAGGGAAAACATGTTCAGCGAAGAGGCCAAGAGCACCTCTATCGCTTTCAGATGCATCAACGAGAACCTCACGCGTTACATCAGCAACATGGACATCTTCGAGAAGGTAACATTCCTTAGTTACCTTTCTTTTCTTTTTCCATCATAAGTTTATAGATTGTACATGCTTTGAGATTTTTCTTTGCAAACAATCTCAGGTGGACGCCATCTTCGATAAGCACGAGGTGCAAGAAATCAAAGAGAAGATCCTCAACAGCGACTACGACGTCGAGGACTTTTTTGAAGGGGAGTTCTTCAACTTCGTTCTCACCCAAGAGGGCATCGACGTGTACAACGCTATTATCGGAGGATTCGTGACCGAGTCTGGGGAGAAGATTAAGGGACTCAACGAGTACATCAACCTGTACAACCAGAAAACGAAGCAGAAGCTCCCGAAGTTCAAGCCGCTCTACAAGCAGGTAAGGATTTTTATGATATACTATGCTTATGTATTTTGTACTGAAAGCATATCCTGCTTCATTGGGATATTACTGAAAGCATTTAACTACATGTAAACTCACTTGATGATCAATAAACTTGATTTTGCAGGTTCTCTCTGATCGTGAGAGCCTCTCATTTTACGGTGAGGGTTACACCTCTGACGAGGAAGTGCTTGAGGTTTTCCGTAACACCCTCAACAAGAACAGCGAGATCTTCTCGTCCATCAAGAAGTTGGAGAAGCTTTTCAAGAACTTCGACGAGTACAGCAGCGCTGGGATCTTCGTTAAGAACGGACCTGCTATCAGCACCATCAGCAAGGATATTTTCGGCGAGTGGAACGTGATCAGGGACAAGTGGAATGCTGAGTACGATGACATCCACCTCAAGAAGAAGGCTGTCGTCACTGAGAAGTACGAGGATGACAGGCGTAAGTCGTTCAAGAAGATCGGCTCTTTCAGCCTCGAGCAGCTTCAAGAATACGCTGATGCTGATCTCAGCGTGGTCGAGAAGCTCAAAGAGATCATCATCCAGAAGGTAAGTTGTTACTTATGATTGTTTTCCTCTCTGCTACATGTATTTTGTTGTTCATTTCTGTAAGATATAAGAATTGAGTTTTCCTCTGATGATATTATTAGGTCGACGAGATCTACAAGGTGTACGGGTCCTCTGAGAAGTTGTTCGATGCTGATTTCGTCCTCGAGAAGAGTCTGAAGAAGAACGACGCTGTCGTCGCGATCATGAAGGATTTGCTCGACAGCGTGAAGTCCTTCGAGAACTATATCAAGGCCTTCTTCGGAGAGGGCAAAGAGACTAATAGGGACGAGTCTTTCTACGGGGATTTCGTGCTCGCTTACGATATCCTCCTCAAGGTTAGTATCATATGAAGAAATACCTAGTTTCAGTTGATGAATGCTATTTTCTGACCTCAGTTGTTCTCTTTTGAGAATTATTTCTTTTCTAATTTGCCTGATTTTTCTATTAATTCATTAGGTGGACCATATCTACGACGCCATCAGAAACTACGTGACCCAGAAGCCTTACAGCAAGGACAAGTTCAAGTTGTACTTTCAGAACCCGCAGTTCATGGGCGGATGGGACAAAGACAAAGAGACAGATTACAGGGCCACCATCCTCAGGTACGGGTCTAAGTACTACCTGGCCATCATGGACAAGAAATACGCCAAGTGCCTCCAAAAGATCGACAAGGATGACGTGAACGGGAACTATGAGAAGATCAACTACAAGCTCCTTCCGGGACCGAACAAGATGCTTCCTAAGGTAAATCCTGGTCCACACTTTTACGATAAAAACACAAGATTTTAAACTATGAACTGATCAATAATCATTCCTAAAAGACCACACTTTTGTTTTGTTTCTAAAGTAATTTTTACTGTTATAACAGGTGTTCTTCAGCAAGAAATGGATGGCCTACTACAACCCGTCTGAGGACATCCAGAAAATCTACAAGAACGGGACCTTCAAGAAAGGCGACATGTTCAACCTCAACGACTGCCACAAGCTCATCGATTTCTTCAAGGACAGCATCTCGCGTTACCCGAAGTGGTCTAACGCTTACGACTTTAACTTCAGCGAGACAGAAAAGTACAAGGATATCGCCGGGTTCTACCGTGAGGTTGAGGAACAGGGTTACAAGGTAAAATATTGGATGCCAGACGATATTCTTTCTTTTGATTTGTAACTTTTTCCTGTCAAGGTCGATAAATTTTATTTTTTTTGGTAAAAGGTCGATAATTTTTTTTTGGAGCCATTATGTAATTTTCCTAATTAACTGAACCAAAATTATACTTTGCAGGTTAGCTTCGAGAGCGCCTCCAAGAAAGAGGTTGACAAGTTGGTCGAAGAGGGCAAGCTCTACATGTTCCAGATCTATAACAAGGACTTCTCCGACAAGAGCCACGGAACTCCTAACCTCCATACGATGTACTTCAAGCTGCTTTTCGACGAGAACAACCACGGGCAGATCAGACTTTCTGGTGGTGCTGAACTCTTCATGCGTAGGGCCTCACTCAAGAAAGAAGAGTTGGTTGTTCACCCGGCCAACTCTCCAATCGCTAACAAGAATCCTGACAACCCGAAAAAGACCACCACGCTGTCTTACGACGTCTACAAGGACAAAAGGTAAGTTCTGCATTTGGTTATGCTCCTTGCATTTTAGGTGTTCGTCGCACTTCCATTTCCATGAATAGCTAAGATTTTTTTTCTCTGCATTCATTCTTCTTGCCTCAGTTCTAACTGTTTGTGGTATTTTTGTTTTAATTATTGCTACAGGTTCAGCGAGGACCAGTACGAGCTTCATATCCCGATCGCTATCAACAAGTGCCCGAAGAACATCTTCAAGATCAATACCGAGGTGAGGGTGCTGCTCAAGCACGATGATAACCCTTACGTGATCGGAATCGATCGTGGTGAGAGAAACCTCCTCTACATCGTTGTGGTGGACGGAAAGGGAAACATCGTCGAGCAGTACAGCCTGAACGAGATTATCAACAATTTCAACGGCATCAGGATCAAGACCGACTACCACTCACTCCTCGATAAGAAAGAAAAAGAGCGTTTCGAGGCCAGGCAGAACTGGACTTCTATCGAAAACATCAAAGAGTTGAAGGCCGGCTACATCTCTCAGGTAAAGCAACTGTGTTTTAATCAATTTCTTGTCAGGATATATGGATTATAACTTAATTTTTGAGAAATCTGTAGTATTTGGCGTGAAATGAGTTTGCTTTTTGGTTTCTCCCGTGTTATAGGTGGTGCATAAGATCTGCGAGCTGGTGGAAAAGTACGATGCTGTGATCGCTCTTGAGGACCTCAACTCTGGGTTCAAGAACAGTAGAGTGAAGGTTGAGAAGCAGGTCTACCAAAAGTTCGAGAAGATGCTCATCGACAAGCTCAACTACATGGTGGACAAAAAGAGCAACCCTTGCGCTACCGGTGGTGCTCTTAAGGGATACCAGATCACGAACAAGTTCGAGTCCTTCAAGAGCATGAGCACCCAGAACGGCTTCATCTTCTATATCCCTGCTTGGCTCACCAGCAAGATCGATCCTTCTACAGGTAATCTTGAAATTGGAACTCTTCTTTTGTTGTCTAAACCTATCAATTTCTTTGCGGAAATTTATTTGAAGCTGTAGAGTTAAAATTGAGTCTTTTAAACTTTTGTAGGTTTCGTGAACCTGCTCAAGACCAAGTACACCTCGATCGCCGACAGCAAGAAGTTCATCTCGTCTTTCGACAGGATCATGTACGTGCCGGAAGAGGATCTTTTCGAGTTCGCTCTCGACTATAAGAACTTCAGCAGGACCGACGCCGACTACATTAAGAAGTGGAAGCTCTACTCCTACGGGAACCGTATCAGGATCTTCCGAAATCCGAAGAAAAACAACGTGTTCGACTGGGAAGAAGTGTGCCTCACCTCTGCCTACAAAGAACTGTTCAACAAGTACGGCATCAACTACCAGCAGGGTGATATCAGGGCTCTTTTGTGCGAGCAGAGCGACAAGGCATTCTACAGCTCATTCATGGCCCTCATGTCTCTCATGCTCCAGATGAGGAACTCTATCACCGGAAGGACCGATGTGGACTTCCTTATCTCTCCGGTCAAGAACTCTGACGGGATCTTCTACGACAGCCGTAACTATGAGGCTCAAGAGAACGCTATCCTGCCGAAGAATGCTGATGCAAACGGGGCTTACAACATTGCGAGAAAGGTCTGTCTTTCCTATTTCATATGTTTAATCCTAGGAATTTGATCAATTGATTGTATGTATGTCGATCCCAAGACTTTCTTGTTCACTTATATCTTAACTCTCTCTTTGCTGTTTCTTGCAGGTTCTCTGGGCTATCGGGCAGTTTAAGAAAGCGGAAGATGAGAAGCTGGACAAGGTGAAGATCGCCATCTCCAACAAAGAGTGGCTTGAGTACGCTCAGACCTCCGTTAAGCACAAGG

**Appendix S17 Open reading frame of *cas12a^At D156R^::introns.***

Nucleotide sequence (5‘ to 3‘) of *cas12a^At D156R^::introns*. First and last codon are shown in red (Start codon ATG, last codon CAC), note that a stop codon is missing in order to fuse a nuclear localization sequence (NLS) on the 3’ end. Introns are highlighted in green**,** AGA = mutation D156R (GAC to AGA), A = sequence change (T>A) to create an additional exon/intron splice-site, CACC = LI fusion site A, AAGG = LI fusion site D, CGTC, TTTT, and ACGG = fusion sites (part of the *cas12a^At D156R^::introns* sequence) to combine the ordered *cas12a^At D156R^::introns* strands, *cas12a^At D156R^::introns* p1, *cas12a^At D156R^::introns* p2, *cas12a^At D156R^::introns* p3 and *cas12a^At D156R^::introns* p4. See table S1 for abbreviations.

**Appendix S18 Reference of the intron sequences in *cas12a^At D156R^::introns.***

The intron sequences were taken from *cas9^Zm^::introns* on pAGM51561 (Grützner et al., 2021). The order of the introns on the *cas12a^At D156R^::introns* sequence was chosen and optimized by using the software NetGene2 to predict highly confident splice acceptor and donor sites. The following table shows which introns of *cas9^Zm^::introns* were used as introns 1 - 11 in *cas12a^At D156R^::introns.* See table S1 for abbreviations.

| **Intron position number**  ***(cas9^Zm^::introns)*** | **Intron position number**  ***(cas12a^At D156R^::introns)*** |
| --- | --- |
| intron 1 | intron 3 |
| intron 2 | intron 2 |
| intron 3 | intron 10 |
| intron 4 | intron 4 |
| intron 5 | intron 5 |
| intron 6 | intron 6 |
| intron 7 | - |
| intron 8 | intron 8 |
| intron 9 | intron 9 |
| intron 10 | - |
| intron 11 | intron 11 |
| intron 12 | intron 7 |
| intron 13 | intron 1 |

(Upstream of *cas12a* ORF)

TCTGaacaATGCTTCAACCTAAGAAGAAGAGAAAGGTTGGAGGAAACACC

M L Q P K K K R K V G G N T

(Downstream *cas12a* ORF)

AAGGGACCTAAGAAGAAGAGAAAGGTTTGAAATC

K G P K K K R K V *

**Appendix S19 Nucleotide sequences and predicted translated product SV40 NLS used for N- and C- terminal tagging of Cas12a.**

The sequences (+ strand, 5‘ to 3‘) of the upstream and downstream SV40 NLS region of the corresponding *Cas* (Figure 5 and 6) are shown with the following colour code: Plant Kozak, SV40, start and stop codon in red and underlined. The coding sequence is *Lotus japonicus* codon adapted. The corresponding amino acid sequence is shown below the nucleotide sequence. LI fusion sites of the Golden Gate system (GG) are highlighted by yellow background and underlined: TCTG = LI fusion site B, CACC = LI fusion site C, AAGG = LI fusion site D, AATC = LI fusion site E. Sequence regions that have not been highlighted are DMY (dummy) sequences that primarily serve to maintain the open reading frame. See table S1 for abbreviations.

(Upstream of *cas12a* ORF)

TCTGaacaATGCTTCAACCTGCTGCTAAGAGAGTTAAGCTTGATGGAGGAAACACC

M L Q P A A K R V K L D G G N T

(Downstream *cas12a* ORF)

AAGGGACCTGCTGCTAAGAGAGTTAAGCTTGATTGAAATC

K G P A A K R V K L D *

**Appendix S20 Nucleotide sequences and predicted translated product c-Myc NLS used for N- and C- terminal tagging of Cas12a.**

The sequences (+ strand, 5‘ to 3‘) of the upstream and downstream c-Myc NLS region of the corresponding *Cas* (Figure 5 and 7) are shown with the following colour code: Plant Kozak, c-Myc, start and stop codon in red and underlined. The coding sequence is *Lotus japonicus* codon adapted. The corresponding amino acid sequence is shown below the nucleotide sequence. LI fusion sites of the Golden Gate system (GG) are highlighted by yellow background and underlined: TCTG = LI fusion site B, CACC = LI fusion site C, AAGG = LI fusion site D, AATC = LI fusion site E. Sequence regions that have not been highlighted are DMY (dummy) sequences that primarily serve to maintain the open reading frame. See table S1 for abbreviations.

(Upstream of *cas12a* ORF)

TCTGaacaATGCTTCAAAAGAGGCCTGCTGCTACTAAGAAGGCTGGTCAAGCTAAGAAGAAGAAGGGAGGAAACACC

M L Q K R P A A T K K A G Q A K K K K G G N T

(Downstream *cas12a* ORF)

AAGGGAAAGAGGCCTGCTGCTACTAAGAAGGCTGGTCAAGCTAAGAAGAAGAAGTGAAATC

K G K R P A A T K K A G Q A K K K K *

**Appendix S21 Sequence of the *Lotus japonicus* codon adapted NLP NLS region.**

The sequences (+ strand, 5‘ to 3‘) of the upstream and downstream NLP NLS region of the corresponding *Cas* (Figure 5 and 7) are shown with the following colour code: Plant Kozak, NLP, start and stop codon in red and underlined. The coding sequence is *Lotus japonicus* codon adapted. The corresponding amino acid sequence is shown below the nucleotide sequence. LI fusion sites of the Golden Gate system (GG) are highlighted by yellow background and underlined: TCTG = LI fusion site B, CACC = LI fusion site C, AAGG = LI fusion site D, AATC = LI fusion site E. Sequence regions that have not been highlighted are DMY (dummy) sequences that primarily serve to maintain the open reading frame. See table S1 for abbreviations.

(Upstream of *cas12a* ORF)

TCTGaacaATGCTTCAAAAGCTTAAGATTAAGAGGCCTGTTAAGGGAGGAAACACCT

M L Q K L K I K R P V K G G N T

(Downstream *cas12a* ORF)

AAGGGAAAGCTTAAGATTAAGAGGCCTGTTAAGTGAAATC

K G K L K I K R P V K *

**Appendix S22 Sequence of the *Lotus japonicus* codon adapted Tus NLS region.**

The sequences (+ strand, 5‘ to 3‘) of the upstream and downstream Tus NLS region of the corresponding *Cas* (Figure 5) are shown with the following colour code: Plant Kozak, Tus, start and stop codon in red and underlined. The coding sequence is *Lotus japonicus* codon adapted. The corresponding amino acid sequence is shown below the nucleotide sequence. LI fusion sites of the Golden Gate system (GG) are highlighted by yellow background and underlined: TCTG = LI fusion site B, CACC = LI fusion site C, AAGG = LI fusion site D, AATC = LI fusion site E. Sequence regions that have not been highlighted are DMY (dummy) sequences that primarily serve to maintain the open reading frame. See table S1 for abbreviations.

(Upstream of *cas12a* ORF)

TCTGaacaATGCTTCAAATGTCTAGAAGAAGAAAGGCTAATCCTACTAAGCTTTCTGAGAATGCTAAGAAGCTTGCTAAGGAGGTTGAGAATGGAGGAAACACCT

M L Q M S R R R K A N P T K L S E N A K K L A K E V E N G G N T

(Downstream *cas12a* ORF)

AAGGGAATGTCTAGAAGAAGAAAGGCTAATCCTACTAAGCTTTCTGAGAATGCTAAGAAGCTTGCTAAGGAGGTTGAGAATTGAAATC

K G M S R R R K A N P T K L S E N A K K L A K E V E N *

**Appendix S23 Sequence of the *Lotus japonicus* codon adapted EGL-13 NLS region.**

The sequences (+ strand, 5‘ to 3‘) of the upstream and downstream EGL-13 NLS region of the corresponding *Cas* (Figure 5) are shown with the following colour code: Plant Kozak, EGL-13, start and stop codon in red and underlined. The coding sequence is *Lotus japonicus* codon adapted. The corresponding amino acid sequence is shown below the nucleotide sequence. LI fusion sites of the Golden Gate system (GG) are highlighted by yellow background and underlined: TCTG = LI fusion site B, CACC = LI fusion site C, AAGG = LI fusion site D, AATC = LI fusion site E. Sequence regions that have not been highlighted are DMY (dummy) sequences that primarily serve to maintain the open reading frame. See table S1 for abbreviations.

TCTGCCCACGACTCACATAGTCCCCCTCAAACACCATGGATCATTATCTTGATATTAGACTTAGACCTGATCCTGAATTTCCTCCTGCTCAACTTATGTCTGTTCTTTTTGGAAAGCTTCATCAAGCTCTTGTTGCTCAAGGAGGAGATAGAATTGGAGTTTCTTTTCCTGATCTTGATGAATCTAGATCTAGACTTGGAGAAAGACTTAGAATTCATGCTTCTGCTGATGATCTTAGAGCTCTTCTTGCTAGACCTTGGCTTGAAGGACTTAGAGATCATCTTCAATTTGGAGAACCTGCTGTTGTTCCTCATCCTACTCCTTATAGACAAGTTTCTAGAGTTCAAGCTAAGTCTAATCCTGAAAGACTTAGAAGAAGATTGATGAGGAGACATGATCTTTCTGAAGAAGAAGCTAGAAAGAGAATTCCTGATACTGTTGCTAGAGCTCTTGATCTTCCTTTTGTTACTCTTAGATCTCAATCTACTGGACAACATTTTAGACTTTTTATTAGACATGGACCTCTTCAAGTTACTGCTGAAGAAGGAGGATTTACTTGTTATGGACTTTCTAAGGGAGGATTTGTTCCTTGGTTTTGAAAGGTTTGATGAGTACCGGAATCACCAGGGGAATC

**Appendix S24 ORF of c*sy4* with flanking 5’ and 3’ targets in *csy4* expression cassette version (I).**

The sequence (+ strand, 5‘ to 3‘), is shown with the following colour code: PAM (Cas9), complement - 5’ target sequence (protospacer), complement - PAM (Cas12a), complement - open reading frame (ORF) of *csy4* *-* PAM (Cas12a) – 3’ target sequence (protospacer) *-* PAM (Cas9)*.* Start and stop codon of the ORF from *csy4* are shown in red and underlined. LI fusion sites of the Golden Gate system (GG) are highlighted by yellow background and underlined: TCTG = LI fusion site B, CACC = LI fusion site C, AAGG = LI fusion site D, AATC = LI fusion site E.

TCTGCCTTGGACTCTCAAATTAACGGCGAAACACCATGGATCATTATCTTGATATTAGACTTAGACCTGATCCTGAATTTCCTCCTGCTCAACTTATGTCTGTTCTTTTTGGAAAGCTTCATCAAGCTCTTGTTGCTCAAGGAGGAGATAGAATTGGAGTTTCTTTTCCTGATCTTGATGAATCTAGATCTAGACTTGGAGAAAGACTTAGAATTCATGCTTCTGCTGATGATCTTAGAGCTCTTCTTGCTAGACCTTGGCTTGAAGGACTTAGAGATCATCTTCAATTTGGAGAACCTGCTGTTGTTCCTCATCCTACTCCTTATAGACAAGTTTCTAGAGTTCAAGCTAAGTCTAATCCTGAAAGACTTAGAAGAAGATTGATGAGGAGACATGATCTTTCTGAAGAAGAAGCTAGAAAGAGAATTCCTGATACTGTTGCTAGAGCTCTTGATCTTCCTTTTGTTACTCTTAGATCTCAATCTACTGGACAACATTTTAGACTTTTTATTAGACATGGACCTCTTCAAGTTACTGCTGAAGAAGGAGGATTTACTTGTTATGGACTTTCTAAGGGAGGATTTGTTCCTTGGTTTTGAAAGGTTTCACGTATCTTCGGCCATGAAGAGGAATC

**Appendix S25 ORF of *csy4* with flanking 5’ and 3’ targets in *csy4* expression cassette version (II).**

The sequence (+ strand, 5‘ to 3‘), is shown with the following colour code: PAM (Cas9), complement - 5’ target sequence (protospacer), complement - PAM (Cas12a) complement - open reading frame (ORF) of *csy4* - PAM (Cas12a) - 3’ target sequence (protospacer) *-* PAM (Cas9)*.* Start and stop codon of the ORF from *csy4* are shown in red and underlined. LI fusion sites of the Golden Gate system (GG) are highlighted in yellow and underlined: TCTG = LI fusion site B, CACC = LI fusion site C, AAGG = LI fusion site D, AATC = LI fusion site E.

**Bibliography**

**Binder, A., Lambert, J., Morbitzer, R., Popp, C., Ott, T., Lahaye, T., and Parniske, M.** (2014). A modular plasmid assembly kit for multigene expression, gene silencing and silencing rescue in plants. PLoS One **9** (2): e88218. http://doi.org/10.1371/journal.pone.0088218.

**Grützner, R., Martin, P., Horn, C., Mortensen, S., Cram, E.J., Lee-Parsons, C.W.T., Stuttmann, J., and Marillonnet, S.** (2021). High-efficiency genome editing in plants mediated by a Cas9 gene containing multiple introns. Plant Commun **2** (2): 100135. http://doi.org/10.1016/j.xplc.2020.100135.

**Schindele, P., and Puchta, H.** (2020). Engineering CRISPR/LbCas12a for highly efficient, temperature-tolerant plant gene editing. Plant Biotechnol J **18** (5): 1118–1120. http://doi.org/10.1111/pbi.13275.

**Tang, X., Lowder, L.G., Zhang, T., Malzahn, A.A., Zheng, X., Voytas, D.F., Zhong, Z., Chen, Y., Ren, Q., Li, Q., Kirkland, E.R., Zhang, Y., and Qi, Y.** (2017). A CRISPR-Cpf1 system for efficient genome editing and transcriptional repression in plants. Nat Plants **3**: 17018. http://doi.org/10.1038/nplants.2017.18.

**Zhang, Y., Chen, M., Siemiatkowska, B., Toleco, M.R., Jing, Y., Strotmann, V., Zhang, J., Stahl, Y., and Fernie, A.R.** (2020). A highly efficient *Agrobacterium*-mediated method for transient gene expression and functional studies in multiple plant species. Plant Commun **1** (5): 100028. http://doi.org/10.1016/j.xplc.2020.100028.

**Zhang, Y., Ren, Q., Tang, X., Liu, S., Malzahn, A.A., Zhou, J., Wang, J., Yin, D., Pan, C., Yuan, M., Huang, L., Yang, H., Zhao, Y., Fang, Q., Zheng, X., Tian, L., Cheng, Y., Le, Y., McCoy, B., Franklin, L., Selengut, J.D., Mount, S.M., Que, Q., Zhang, Y., and Qi, Y.** (2021). Expanding the scope of plant genome engineering with Cas12a orthologs and highly multiplexable editing systems. Nat Commun **12** (1): 1944. http://doi.org/10.1038/s41467-021-22330-w.
